# Discovery of 6α-Thiazolylcarboxamidonaltrexamine Derivative (NTZ) as a Potent and Central Nervous System Penetrant Opioid Receptor Modulator with Drug-like Properties for Potential Treatment of Opioid Use Disorder

**DOI:** 10.1021/acsptsci.4c00593

**Published:** 2024-12-05

**Authors:** Boshi Huang, Hongguang Ma, Piyusha P. Pagare, Mengchu Li, Rolando E. Mendez, James C. Gillespie, Justin L. Poklis, Matthew S. Halquist, David L. Stevens, William L. Dewey, Dana E. Selley, Yan Zhang

**Affiliations:** † Department of Medicinal Chemistry, School of Pharmacy, Virginia Commonwealth University, 800 E Leigh Street, Richmond, Virginia23298, United States; ‡ Department of Pharmacology and Toxicology, School of Medicine, Virginia Commonwealth University, 410 North 12th Street, Richmond, Virginia23298, United States; # Department of Pharmaceutics, Virginia Commonwealth University, 410 North 12th Street, Richmond, Virginia23298, United States; & Institute for Drug and Alcohol Studies, 203 East Cary Street, Richmond, Virginia23298-0059, United States

**Keywords:** opioid use disorders, mu opioid receptor, antagonist, structure activity relationship, CNS permeability, withdrawal

## Abstract

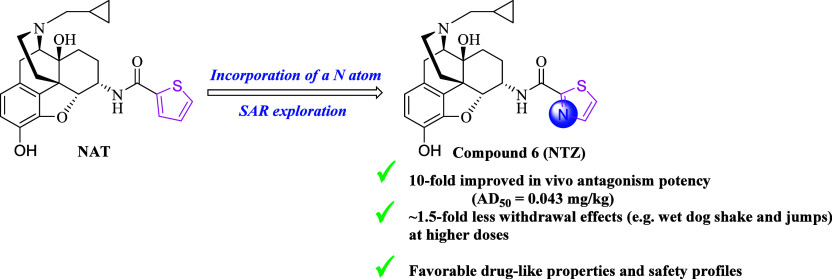

The development of highly potent and selective μ opioid receptor (MOR) modulators with favorable drug-like properties has always been a focus in the opioid domain. Our previous efforts led to the discovery of a lead compound designated as NAT, a potent centrally acting MOR modulator. However, the fact that NAT precipitated considerable withdrawal effects at higher doses largely impaired its further development. In the light of the concept of activity cliff and CNS multiparameter optimization algorithm, a nitrogen atom was incorporated into the thiophene ring of NAT, aiming to preserve desirable pharmacological activities and CNS permeability while alleviating withdrawal symptoms. Among all 16 new analogs, compound **6** (NTZ) exhibited improved opioid receptor selectivity, enhanced *in vivo* antagonistic effect, and overall fewer withdrawal symptoms compared to NAT. Further assessment of several key drug-like properties suggested a favorable ADMET profile of NTZ. Taken together, NTZ shows promise as a potential lead to treat opioid use disorder.

Opioid use disorder (OUD) is a problematic pattern of opioid misuse that causes clinically serious impairment or distress. OUD remains an enormous threat to public health worldwide with particularly about 6.1 million people in the U.S. suffering from this crisis.^1^ It was reported that opioids were involved in approximately 76% of drug overdose deaths in 2022 in the U.S.^2^ The number of opioid-involved overdose deaths has increased steadily over years with about 81,806 deaths reported in 2022.^1^ The misuse of opioids, including prescription pain killers and abuse of various synthetic opioids, has posed a tremendous burden on the economy and society, highlighting an urgent demand of efficacious and safe medications to treat OUD.

Currently, two main approaches to treat OUD, i.e. detoxification and maintenance therapy, include applications of opioid receptor full agonists,^3,4^ partial agonists,^5^ or antagonists.^6^

Buprenorphine and methadone (Figure 1), mu opioid receptor (MOR) partial and full agonists, respectively, are first-line opioid medicines for the treatment of OUD, demonstrating favorable efficacy for maintenance therapy.^7^ Although a low efficacy partial agonist like buprenorphine would be expected to precipitate lesser magnitude of withdrawal since it only partially activates the receptor population and therefore does not completely reverse the effect of an agonist, some undesirable effects including respiratory depression, a long period of precipitated withdrawal symptoms, and relapsing disorder of patients after being subjected to treatment of OUD have compromised the application of methadone and buprenorphine.^7,8^ Alternately, opioid antagonists naltrexone (NTX) and naloxone (NLX) (Figure 1) find application in either as overdose reversal agents or for medication assisted treatment (MAT).^9−11^ They have demonstrated good efficiency in the management of opioid use disorders, while possessing adverse effects such as dizziness, dysphoria, depression, and the most common one, withdrawal symptoms, including nausea/vomiting, diarrhea, anxiety and so on.^12,13^ Moreover, high doses of both drugs may further contribute to hepatotoxicity as well as cardiovascular and pulmonary complications.^12,14^ Collectively, these drugs have associated drawbacks in their applications, although they have demonstrated that targeting the MOR may provide effective treatment for OUD. Therefore, highly potent and selective MOR modulators with favorable drug-like properties and devoid of unwanted side effects are still extremely desirable.

**Figure 1:**
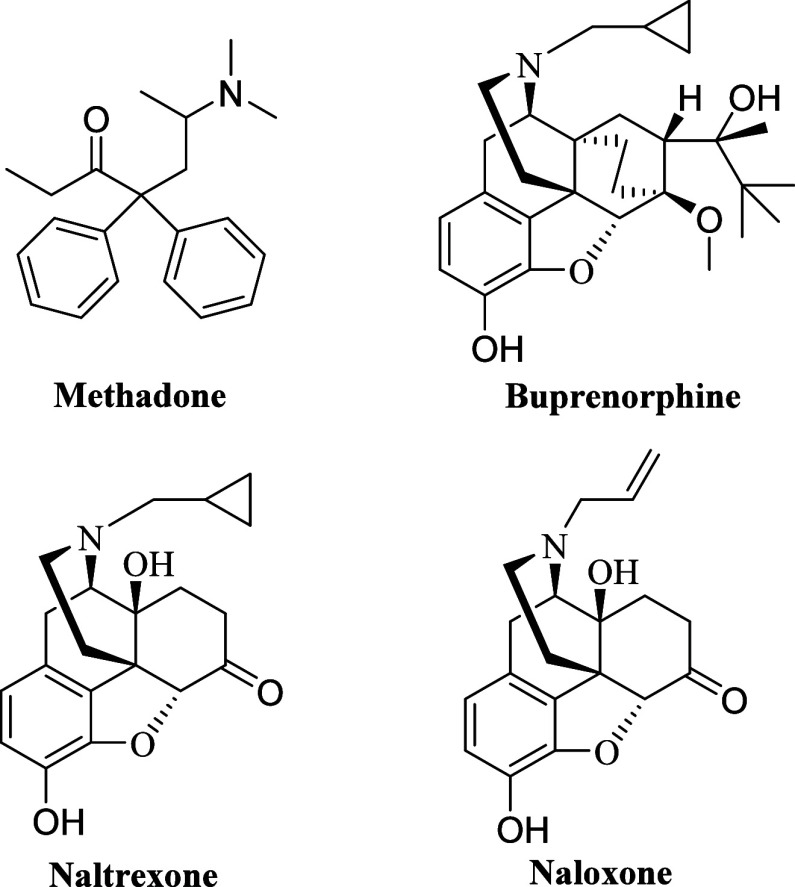
Chemical structures of methadone, buprenorphine, naltrexone and naloxone.

Our research has been dedicated to exploring epoxymorphinan analogues featuring diverse heterocyclic ring systems at the C6 position, guided by the “message-address” concept, with the goal of discovering new opioid receptor ligands that exhibit high potency and selectivity.^15−21^ Our efforts first lead to the discovery of NAP (Figure 2), which was endowed with subnanomolar binding affinity to the MOR and high MOR selectivity over the kappa opioid receptor (KOR) and the delta opioid receptor (DOR) *in vitro*. Further pharmacological characterizations of NAP revealed that it displayed moderate centrally acting potency with an AD_50_ value of 4.51 mg/kg to antagonize the antinociception elicited by morphine,^15^ while demonstrating a remarkable peripherally acting activity in a gastrointestinal (GI)-tract motility assay (ED_50_ = 0.0088 mg/kg),^22^ suggesting its significant peripheral selectivity.

**Figure 2:**
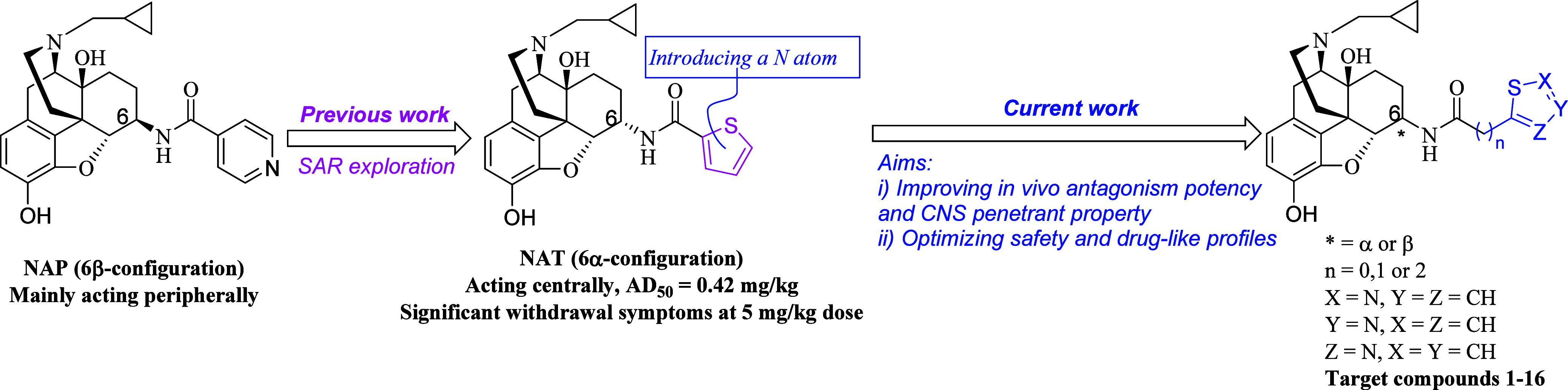
Previous and current medicinal chemistry approaches to discover new-generation MOR ligands.

We therefore conducted further structure–activity relationship (SAR) studies to dissociate NAP’s central nervous system (CNS)-favored properties from the peripheral nervous system (PNS)-favored properties in the past decade. Most recently, a potent MOR ligand possessing a thiophene ring at the *C6*-position of the epoxymorphinan skeleton, NAT (corresponding to compound **25** in our recently published work),^20^ was identified through comprehensive pharmacological characterizations (Figure 2). NAT showed high binding affinity to the MOR, moderate selectivity for the MOR over the KOR and the DOR, and low-efficacy partial agonism for the MOR *in vitro*. It was able to potently antagonize morphine-induced antinociception with an AD_50_ (dose producing a 50% anti-antinociceptive response) value of 0.42 mg/kg. However, although NAT displayed fewer withdrawal symptoms than NLX when tested at the same dose of 1 mg/kg,^20^ it exhibited significant withdrawal symptoms at higher doses, warranting further structural modifications to yield potent CNS-acting MOR modulators with improved overall safety profiles.

In medicinal chemistry practice, the concept of activity cliff (AC) has been proposed over decades to describe the phenomenon where subtle chemical modifications may lead to surprisingly significant differences in compounds’ potency.^23,24^ During early stages of compound optimization efforts, ACs are likely to be encountered through intensive SAR study, which has been perceived to facilitate identifying minor but critical chemical modifications that determine the potency of target compounds. In this context, it would be interesting to conduct a comprehensive exploration on the SAR of NAT analogs based on the concept of AC to examine the potential impact on the potency of NAT analogs.

On the other hand, the replacement of a *CH* group with a nitrogen atom in aromatic and heteroaromatic ring systems, also referred to as “the necessary nitrogen atom”,^25^ has shown significant influence on the physicochemical and pharmacological profiles in lead identification and optimization.^26−28^ Recently, a retrospective analysis of FDA-approved drugs has revealed the versatile role of nitrogen atom in multiparameter optimization (MPO) in drug discovery campaigns.^29,30^ Moreover, a number of paradigms have been reported to demonstrate the profound influence of inclusion of nitrogen atoms into structural alerts such as heterocycles on tackling concerns of their bioactivation and metabolic toxicity.^30^ Keeping in mind that we aim to discover centrally acting MOR ligands, introduction of a nitrogen atom would be further examined for its influence on the CNS permeability. Herein, we report our most recent progress on development of CNS-acting MOR modulators that preserve desirable pharmacological and CNS penetrant properties as well as enhanced safety profiles through applying the bioisosterism principle by incorporating a nitrogen atom into the thiophene ring of NAT.

## Results and Discussion

### Rational Drug Design

Our previous computational studies postulated that the pyridyl ring of NAP may be critical in MOR binding and selectivity through a π–π stacking interaction with Trp318^7.35^ in addition to a plausible hydrogen bonding with the positively charged Lys303^6.58^.^31,32^ Subsequently, replacement of the pyridyl ring of NAP with a thiophene ring yielded a potent CNS-acting MOR ligand NAT, indicating that the heteroaromatic ring in the *C6* side chain should be preserved in the lead identification efforts to generate potential CNS-targeted MOR modulators.^20^

The other important aim of our present work is to furnish MOR modulators permeable through the blood-brain barrier (BBB), which is a crucial physical barrier protecting the CNS from circulating toxins and pathogens in the bloodstream.^33,34^ A weighted scoring approach, CNS multiparameter optimization (MPO) algorithm, has been widely used in the assessment of the potential of small molecules penetrating the BBB.^35,36^ It is a well-defined and developed approach using *in silico* prediction and calculation derived from the physicochemical properties of chemical entities. Six critical physicochemical properties, including cLogP, cLogD, tPSA (topological polar surface area), MW (molecular weight), HBDs (number of hydrogen-bond donors), and p*K*_a_, are used in this algorithm, and each property is defined with values ranging from 0 to 1. Thus, the total score of one small molecule may be between 0 and 6, with an MPO desirability score ≥4 having been extensively applied to select hit compounds toward CNS drug discovery.^36^ Application of the CNS MPO algorithm may provide a rational and feasible approach for balancing multiple variables and could be therefore leveraged in drug design programs. In this context, we previously rationally designed and synthesized a series of epoxymorphinan derivatives carrying a pyrazolyl or imidazolyl ring, which possessed CNS MPO scores all lower than 4.0 and were shown to have the potential in the treatment of opioid induced constipation with peripheral selectivity.^37^ On the other hand, it has been found that NAT has a CNS MPO score of 3.8, which seems not in agreement with its CNS dominated characteristics as validated in prior *in vivo* study results.^20^ Therefore, the CNS MPO score calculation approach was applied in our current study in order to further test its applicability in epoxymorphinan derivatives.

Taking all these into account, we decided to introduce a nitrogen atom into the thiophene ring of NAT, i.e. the thiophene ring in NAT was replaced by the isothiazolyl or thiazolyl rings, in order to further tempering of the CNS-acting properties through somehow delicate changes on the MPO scores of the newly designed compounds. We reasoned that the aromatic nature of isothiazolyl and thiazolyl rings could possibly maintain the π–π stacking interaction with the key residue W318^7.35^. Meanwhile the incorporated nitrogen atom could potentially introduce hydrogen bonding interactions with residue K303^6.58^ which is unique to the MOR and absent in the KOR and DOR. These additional interactions could preserve or even enhance selectivity for the MOR over the KOR and the DOR. It should be noted that the position of the sulfur atom was kept unchanged corresponding to the amide linker in the newly designed compounds, so to focus more on the influence of the nitrogen atom on pharmacological activities. Additionally, our prior SAR endeavor has suggested that the role of the stereochemistry at *C6*-epoxymorphinan skeleton has not been conclusive. Hence the analogs of NAT with either α or β configuration were designed and pharmacologically evaluated.^15,17−21^ The carboxamido, acetamido, or n-propanamido group as the linker between the heteroaromatic ring (the “address” moiety) and the epoxymorphinan core structure (the “message” moiety) was applied to investigate the favorable physiochemical features of NAT analogs as CNS-acting agents. It was revealed that the newly designed compounds **1**–**16** (Figure 2) possessed calculated CNS MPO scores with a range from 3.3 to 3.5, all close to that score of NAT (3.8) while lower than 4, thus warranting further biological characterization (Table S5).

### Chemistry

**Scheme 1:**
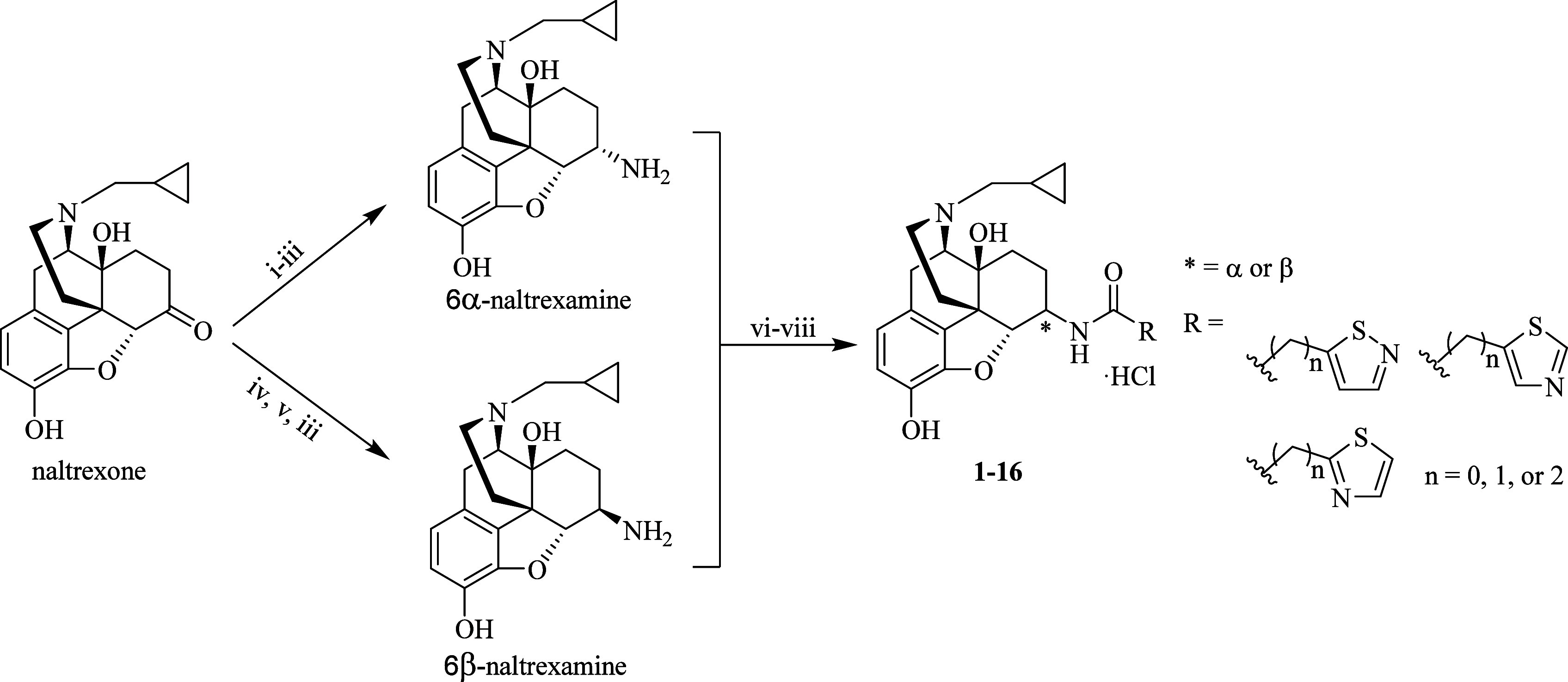
Syntheses of the Target Compounds **1-16**. (i) Benzylamine, *p*-TsOH, Benzene, Reflux. (ii) NaBH_4_, 4 Å MS, EtOH, r.t. (iii) H_2_, HCl, Pd/C, MeOH, r.t. (iv) Dibenzylamine, PhCOOH, *p*-TsOH, Toluene, Reflux. (v) NaCNBH_3_, 4 Å MS, EtOH, r.t. vi) RCOOH, EDCI, HOBt, TEA, 4 Å MS, DMF, r.t. vii) K_2_CO_3_, MeOH, r.t. viii) 1.25 M HCl/MeOH, 0 °C to r.t

The synthetic route of the newly designed compounds **1**–**16** was outlined in Scheme 1 following our established protocols: Initially, 6α- or 6β-naltrexamine (NTA) was synthesized through the reductive amination of naltrexone with either benzylamine or dibenzylamine, followed by debenzylation via catalytic hydrogenation.^15^ Then, 6α- or 6β-NTA was coupled with various commercially available isothiazolyl- or thiazolyl-bearing carboxylic acids via EDCI/HOBt coupling reaction. The disubstituted intermediates at the phenolic oxygen were hydrolyzed by treatment with potassium carbonate in methanol, yielding the 6-position monosubstituted free bases in good yields. These free bases were then converted to their hydrochloride salts using HCl in methanol, thoroughly characterized, prior to in vitro and in vivo biological evaluations.

### *In Vitro* Radioligand Binding and MOR [^35^S]GTPγS Functional Assays

To assess the binding affinity and selectivity profiles of the target compounds across the three opioid receptors in vitro, MOR, KOR and DOR, competitive radioligand binding assays were conducted as previously reported.^17^ The [^35^S]GTPγS functional assay was then performed to assess the agonist potency and efficacy for MOR activation of each target compound by determining its efficacy relative to the MOR full agonist DAMGO. The results are summarized in Tables 1 and 2.

**Table 1:**
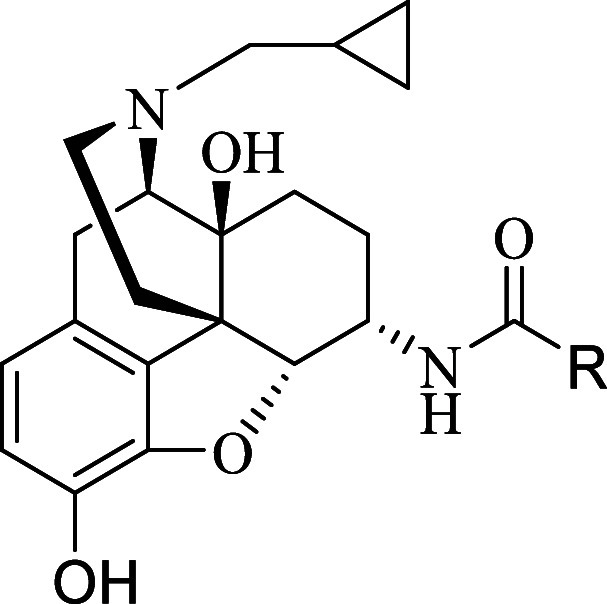

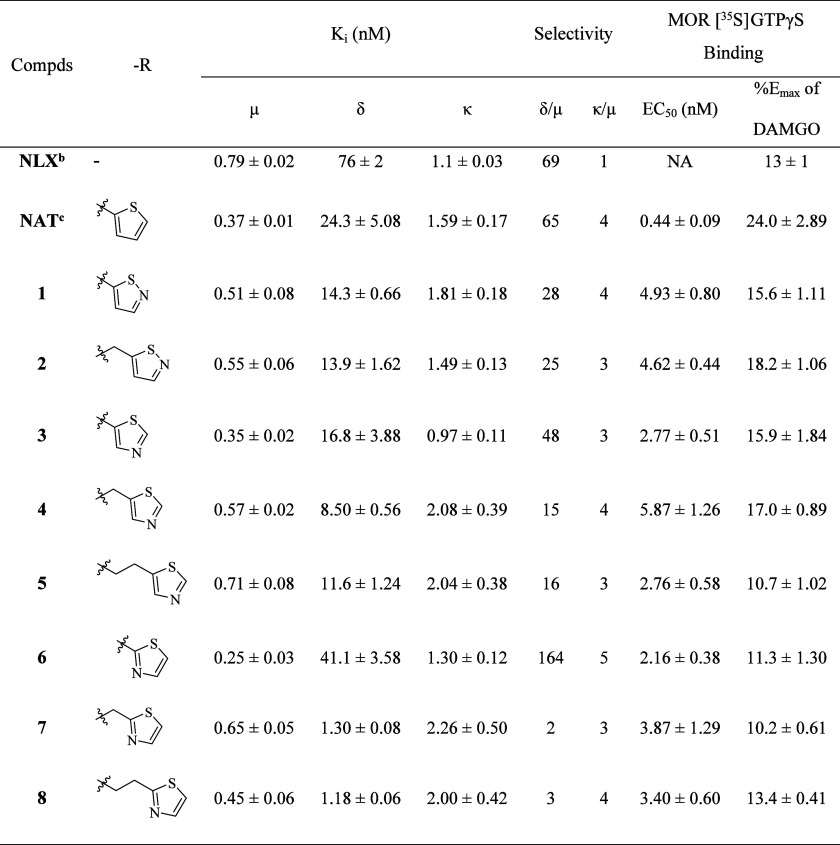
Binding Affinity, Selectivity, and MOR [^35^S]GTPγS Functional Assay Results of Compounds 1-8 (6α-Configuration)^a^

a Data reported as the mean ± SEM of at least three independent experiments.

b,c Data have been reported in references,^20,38^ and are provided here for comparative analysis.

**Table 2:**
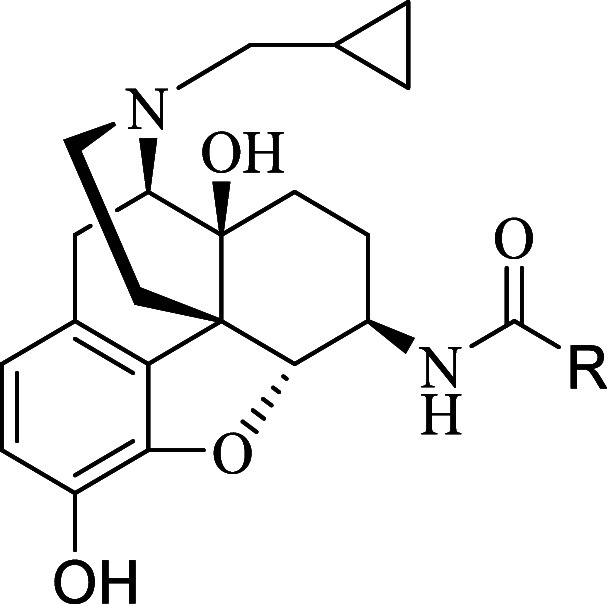

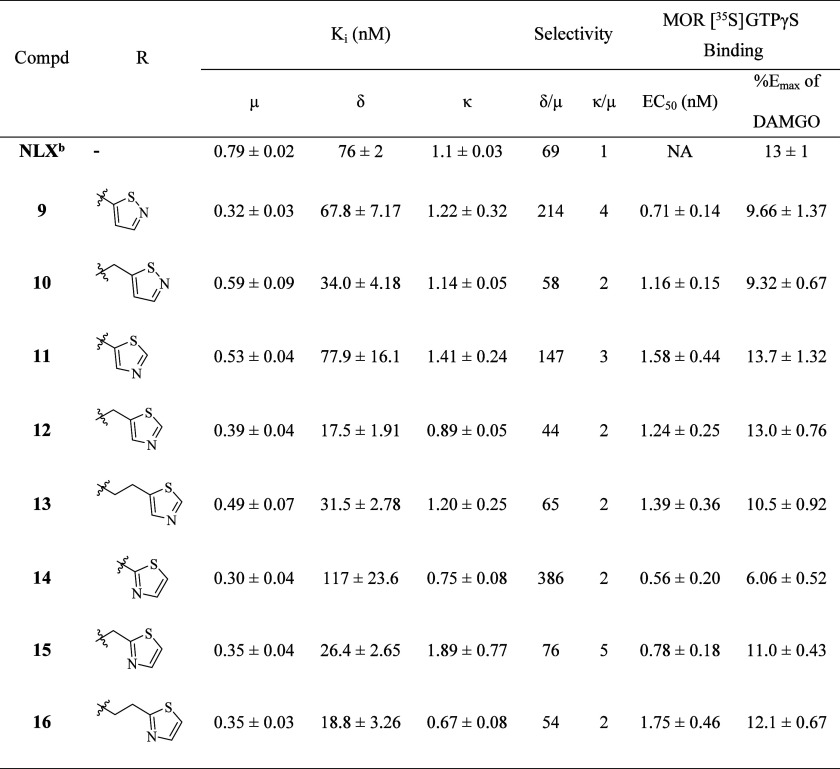
Binding Affinity, Selectivity, and MOR [^35^S]GTPγS Functional Assay Results of Compounds 9-16 (6β*-*Configuration)^a^

a Data reported as mean ± SEM of at least three independent experiments.

b Data have been reported in references,^20,38^ and are provided here for comparative analysis.

As seen in Table 1, all compounds **1**–**8** with a 6α-configuration exhibited subnanomolar binding affinity at the MOR and single digit nanomolar binding affinity at the KOR, which were comparable to those of NAT. Moreover, except for **7** and **8**, other compounds demonstrated much lower binding affinity to the DOR, thus preserving reasonable selectivity at the MOR over the DOR. More importantly, compounds with a carboxamido linker, e.g. compounds **3** and **6**, demonstrated higher δ/μ selectivity than their counterparts with longer linkers. It was worth noting that compound **6** displayed the highest δ/μ selectivity, which was about 3-fold higher than that of NAT. Lastly, no obvious trend was observed for the binding affinity for the MOR of those compounds with the nitrogen atom at different positions of the heterocyclic rings (**1** vs **3** vs **6** and **2** vs **4** vs **7**).

For the 6β-configuration analogs **9**–**16** (Table 2), it was observed that all of them retained high binding affinity for the MOR which was comparable to that of NAT. In addition, the similar KOR binding profile was also preserved for compounds **9**–**16** relevant to NAT. Interestingly, the binding affinities of **9**–**16** at the DOR were all lower than those of their 6α-counterparts **1**–**8**, thereby exhibiting higher δ/μ selectivity patterns. In particular, compounds **9, 11** and **14** all showed >100-fold selectivity of the MOR over the DOR, of which **14** possessed the highest selectivity feature. Again, the position of the nitrogen atom within the heterocyclic ring appeared to have minimal impact on receptor binding and selectivity profiles. Collectively, all newly prepared compounds incorporating a nitrogen atom exhibited comparable binding affinity at the MOR, retained selectivity for the MOR over the KOR, and displayed comparable or even higher selectivity for the MOR over the DOR, compared to the original hit NAT.

As shown in Tables 1 and 2, all compounds showed nanomolar to subnanomolar potency and considerably low efficacy with % *E*_max_ values ranging from 6.06 ± 0.52 to 18.2 ± 1.06 in the functional assays. Their relatively lower efficacy (% *E*_max_) profile than NAT indicated that they may act as MOR modulators with lower abuse liability, which warrants further evaluations. Additionally, it appeared that the stereochemical characteristic of *C6*-position of the core skeleton to some degree may have impacts on potency and efficacy. For instance, 6α-analogs having the same C6-configuration as NAT exhibited higher efficacies than their 6β-counterparts (except for **15**). On the other hand, 6β-analogs basically demonstrated enhanced potencies than their 6α*-*counterparts. Overall, the introduction of a nitrogen atom into the thiophene ring of NAT has expectantly yielded a series of analogs with retained strong binding affinity, selectivity, and reduced efficacies at the MOR.

### *In Vivo* Warm-Water Tail Immersion Assay

**Figure 3:**
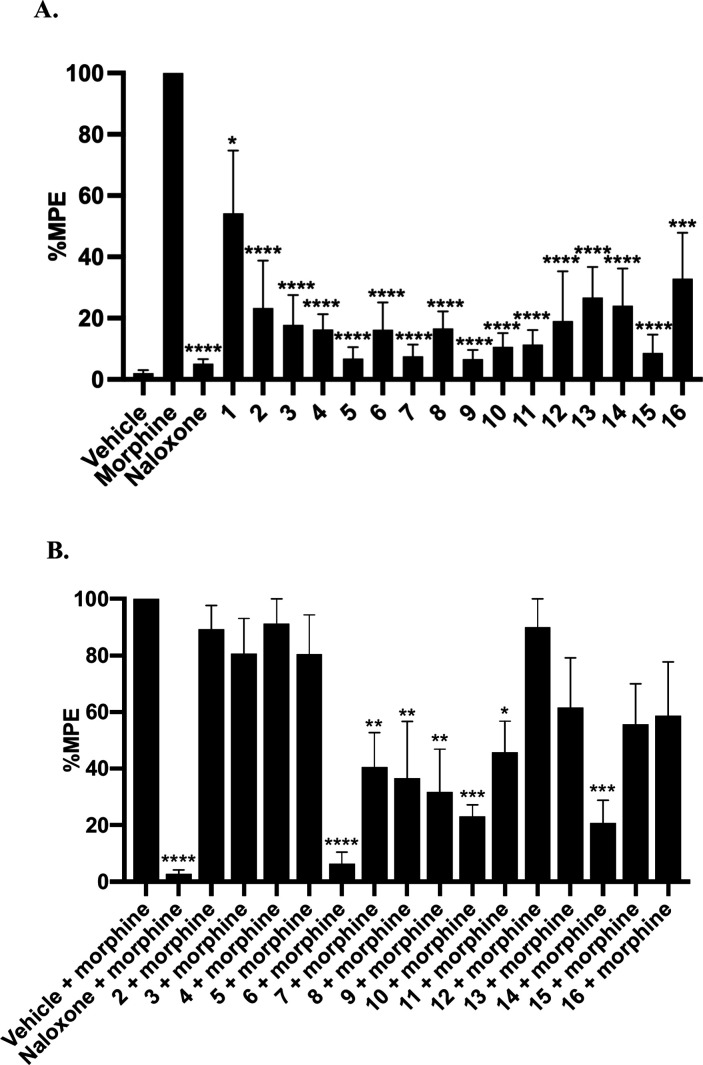
Warm-water tail immersion assay results of compounds as (A) agonists at a single dose of 10 mg/kg s.c. and, (B) antagonists at a single dose of 10 mg/kg s.c. in the presence of morphine (10 mg/kg). Saline and morphine were used as the negative and positive controls, respectively. (*n* = 6) Data are presented as mean values ± SD *P, **P, ***P, ****P represent <0.05, < 0.01, < 0.0005, < 0.0001 compared to 10 mg/kg morphine (s.c.) respectively. %MPE value of NAT was reported as 15.2 ± 8.8 in single dose agonism assay; %MPE value of NAT was reported as 2.1 ± 1.7 in single dose antagonism assay, with the %MPE value of NLX being 2.9 ± 1.3.^20^

The warm-water tail immersion pain model, which recorded the time span in which mice keep the tails in the warm water at different doses of tested agents, has been widely utilized in previous studies to assess their acute agonistic or antagonistic effects.^20,39,40^ All 16 compounds were therefore first subjected to the acute antinociception tail immersion test by adopting subcutaneous administration route. As shown in Figure 3A, all compounds except for **1**, exhibited significantly less antinociceptive effects with much lower maximum possible effects (MPE) compared to the morphine treated control at a single dose of 10 mg/kg. The antinociceptive responses for compound **1** showed considerable variability across subjects as reflected by the error bar. Despite this variability, the overall antinociceptive effect of compound **1** remained significantly lower than that of morphine (*P < 0.05). These observations well corresponded well to its in vitro very low efficacy in activating the MOR (Table 1, 2). The remaining 15 compounds were then tested for their capability of antagonizing antinociception effect elicited by morphine. As depicted in Figure 3B, compounds **6**, **7**, **8**, **9**, **10**, **11** and **14**, significantly antagonized morphine’s antinociception effects. Of interest, three of them, i.e. **6**, **9** and **14** possessed a carboxamido linker between the heterocyclic rings and the epoxymorphinan scaffold, which clearly highlighted the necessity of maintaining carboxamido as a linker in the forthcoming rational molecular design. Their ability to antagonize morphine’s antinociceptive effect was shown to be dose-dependent in the follow-up dose–response study with AD_50_ values ranging from 0.043 to 4.77 mg/kg (Table 3 and Figure S2). Compounds **6**, **9**, **10**, and **14** demonstrated in vitro efficacies with an Emax value range of 6–11% (Tables 1 and 2), indicating them acting as MOR antagonists. However, their in vivo potencies varied significantly. We postulate that this variability is largely attributable to differences in their pharmacokinetic properties, with compound **10**, in particular, displaying less favorable characteristics compared to the others. Of note, compound **6** containing a 2′- thiazolyl moiety acted as the most potent member in this series of NAT analogs in antagonizing morphine mediated antinociception effect. Its AD_50_ value was shown as 0.043 mg/kg, 10 times more potent compared to NAT. Owing to its superior potency in in vivo study, compound **6** stood out as the most active expoxymorphinan-based molecule among all MOR modulators that have ever been discovered by our group.

**Table 3:** AD_50_ Values of Compounds for Counteracting Morphine-Induced Antinociception

Compound	AD_50_ mg/kg (95% CL)
NAT^a^	0.42 (0.21–0.82)
NLX	0.05 (0.03–0.09)
**6**	0.043 (0.007–0.25)
**9**	4.77 (3.59–6.34)
**10**	13.12 (2.34–73.39)
**14**	0.23 (0.04–1.37)

a Data have been reported in reference^20^ and,^41^ and are provided here for comparative analysis.

### MOR Calcium Flux Assay

Activation of the MOR is reported to result in a variety of effects, such as inhibition of the voltage-gated calcium channels.^42^ The second-messenger assay measuring intracellular calcium levels has become an important approach for evaluating opioid receptor functions.^43^ To further characterize the three chosen potent modulators, compounds **6**, **9**, and **14**, a calcium flux assay was conducted using our standard procedure.^19^ As shown in Figure S1A, none of them showed any significant agonistic activity in increasing intracellular calcium concentration when compared to the full MOR agonist DAMGO. On the other hand, they were all shown to antagonize DAMGO-induced intracellular calcium increase in a dose-dependent manner (Figure S1B). In detail, all three compounds, **6**, **9** and **14** were more potent than the parent compound NAT (IC_50_ 42.11 ± 4.51 nM) with IC_50_ values of 5.64 ± 0.24 nM, 6.53 ± 2.28 nM, 12.37 ± 1.18 nM, respectively. Interestingly, these compounds behaved as very low efficacy partial agonists in the [^35^S]GTPγS assay (Table 1, 2). Although it is well accepted that binding of GTPγS in membrane preparations is probably the most sensitive method to distinguish partial agonists and neutral antagonists,^44−46^ the determination of agonist potencies and efficacies in artificial receptor or G-protein overexpression systems may vary significantly and the level of signaling cascade that is analyzed has an impact on the quantification of agonist properties.^46^ Nevertheless, all 3 compounds demonstrated very low efficacy in the GTPγS assay which can also be attributed to the difference in the DAMGO concentrations used between these studies. Overall, this profile observed for compounds **6**, **9** and **14** in both in vitro functional studies appeared to be in line with the results from the in vivo tail immersion studies.

### KOR and DOR [^35^S]GTPγS Functional Assays

To better understand the in vivo effects of compounds 6, 9, and 14 observed in the warm-water tail immersion assay, their potency and efficacy at the KOR and DOR were evaluated using the KOR and DOR [35S]GTPγS functional assays. U50488H, a full agonist for KOR, and SNC80, a full agonist for DOR, were used as control compounds. The results are shown in Table 4.

**Table 4:** KOR and DOR [^35^S]GTPγS Functional Assay Results of Compounds 6, 9, and 14 and NAT.^a^

	Selectivity		KOR [^35^S]GTPγS binding		DOR [^35^S]GTPγS binding	
Compds	κ/μ	δ/μ	EC_50_ (nM)	% *E*_max_ of U50488H	EC_50_ (nM)	% *E*_max_ of SNC80
**NAT**^b^	4	65	1.7 ± 0.24	50.6 ± 0.95	59.4 ± 6.71	48.6 ± 3.41
**6**	5	164	3.83 ± 0.62	23.5 ± 2.03	23.6 ± 4.29	46.5 ± 2.17
**9**	4	214	7.06 ± 0.18	49.9 ± 1.99	58.1 ± 10.3	44.4 ± 4.15
**14**	2	386	9.67 ± 1.81	39.8 ± 2.82	44.0 ± 6.82	25.9 ± 0.40

a Data reported as mean ± SEM of at least three independent experiments.

b Data have been reported in reference,^20^ and are provided here for comparative analysis.

According to the KOR [^35^S]GTPγS binding assay results, all three compounds demonstrated low to moderate efficacy with single-digit nanomolar potencies compared to those of NAT. Compound **6** possessed the most potent functional activity with the lowest efficacy among these three NAT analogs. Given that appropriate activation of KOR may be beneficial in the treatment of opioid addiction and opioid-induced pruritus,^47−50^ the KOR partial agonism of these compounds suggested their potential to treat OUD.

As for the DOR, all three newly synthesized compounds exhibited double-digit nanomolar potencies with low to moderate efficacy ranging from 25.9% to 46.5% in the [^35^S]GTPγS functional assays. Among them, compound **6** exhibited a slightly improved potency at the DOR but with a comparable efficacy compared to NAT. Meanwhile, compound **9** showed both comparable potency and efficacy at the DOR in comparison with NAT. In addition, compound **14** behaved as a low-efficacy DOR partial agonist with slightly elevated potency compared to NAT. Since all three compounds possessed high selectivity for the MOR over the DOR with none showing high potency and high efficacy at the DOR (Table 4), we reasoned that their likelihood of causing severe adverse effects such as convulsion might be low. Collectively, the antagonistic profiles of these NAT derivatives *in vitro* and *in vivo* could be largely due to their intrinsic antagonism properties against the MOR.

### *In Vivo* Opioid Withdrawal Studies

As aforementioned, naloxone and naltrexone, two MOR neutral antagonists may produce significant withdrawal symptoms when applied in clinical practice, which leads to patient compliance issues during OUD treatments. Considering their remarkable MOR antagonistic effects *in vivo*, compounds **6** and **14** were further selected to evaluate whether they would precipitate withdrawal effects. Somatic symptoms of opioid withdrawal, including wet-dog shakes, escape jumps, and paw tremors in morphine-pelleted mice, were recorded over a twenty-minute period after each subcutaneous administration with each compound.^20,21^ Naloxone was used as control and precipitated significant withdrawal symptoms at a dose of 1 mg/kg similar to our previous reports.^17,19,20^

**Figure 4:**
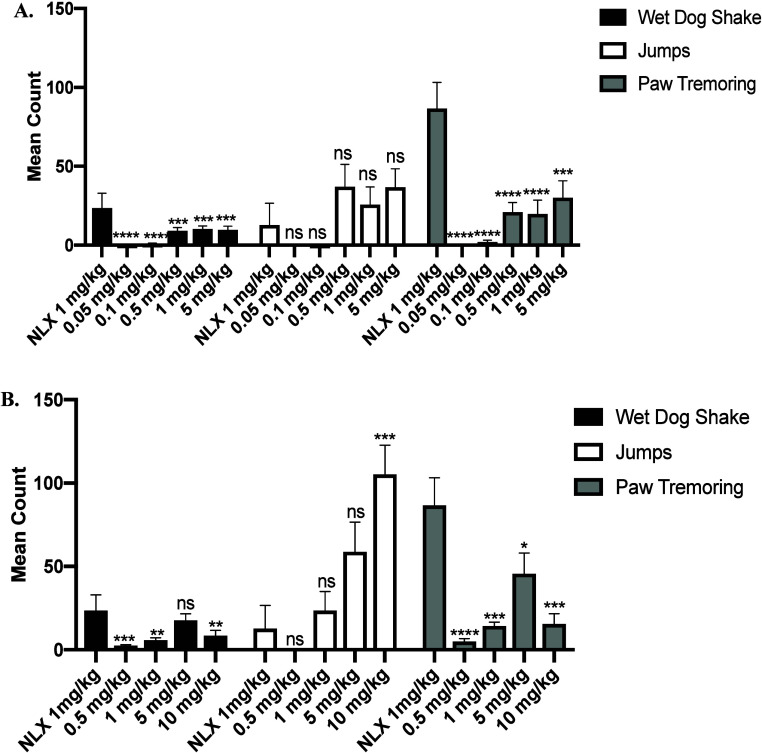
In vivo withdrawal assays of compounds (A) **6** and (B) **14** in morphine-pelleted mice (*n* = 6), including wet dog shakes, jumps and paw tremoring. All compounds were administered s.c. *P, **P, ***P, ****P represent <0.05, < 0.01, < 0.0005, < 0.0001 compared to 10 mg/kg morphine (s.c.) respectively, and ns= not significant compared to 1 mg/kg naloxone (NLX; s.c.).

As shown in Figure 4, compounds **6** and **14** both exhibited fewer wet dog shakes and paw tremors at all testing doses than 1 mg/kg NLX, even at the highest doses of 5 mg/kg and 10 mg/kg, respectively. The mean count values of NAT at 5 mg/kg dosage in wet dog shake, jumps and paw tremors were 14.5, 45.8 and 29.2, respectively, those of compound **6** were 9.8, 36.8 and 30.2, respectively and those of compound **14** were 17.7, 58.8 and 45.7, respectively.

Of note, both newly synthesized compounds exhibited fewer wet dog shakes and paw tremors compared to NLX, even at higher doses such as 5 mg/kg.^20^ Compounds **6** and **14** at the lower doses of 0.5 mg/kg and 1 mg/kg produced jumps similar to those seen with 1 mg/kg NLX. It is worth noting that a 1 mg/kg dose of compound **6** produced considerably less wet dog shakes, jumps and paw tremors in comparison to 1 mg/kg naltrexone (NTX) according to our prior *in vivo* study results.^51^ Lastly and most importantly, although compound **6** and NLX showed similar potency in the warm water tail immersion studies (Table 3), compound **6**, even at a much higher dose of 5 mg/kg, caused fewer withdrawal symptoms compared to NLX at 1 mg/kg. We propose that multiple mechanisms may account for the reduced withdrawal effects observed with compound **6**. One possible explanation is that low-efficacy agonists, like buprenorphine, are known to induce less dependence and withdrawal during chronic treatment compared to neutral antagonists. Buprenorphine, a mixed mu agonist-antagonist, is thought to cause milder withdrawal partly due to its slow dissociation from MOR.^52−54^ In vitro studies suggest compound **6** acts as a very low-efficacy mu agonist, while in vivo studies indicate it may function as a mixed agonist-antagonist. This unique profile of compound **6** may contribute to its reduced withdrawal potential. Although this hypothesis is based on current data, further detailed pharmacological characterization is required to fully understand the mechanisms involved. While compound **6**, as low-efficacy agonist or antagonist, is likely to cause some withdrawal as seen by the increased jumps, our current in vivo study indicates that compound **6** has an improved profile in withdrawal studies especially with respect to wet dog shakes and paw tremoring compared to the parent compound, NAT, suggesting its potential application in OUD treatment.

### BBB-Penetration Studies

Based on the overall remarkable *in vitro* and *in vivo* pharmacological activities, compound **6** was selected to examine its ability to penetrate the BBB, as our goal is to develop CNS-acting MOR modulators. The passive permeability of compound **6** was first assessed by adopting a Caco-2 cell model, which has been increasingly utilized to preliminarily assess the capability of drugs penetrating the BBB.^55^ It was revealed that compound **6** exhibited a *P*_app,A-B_ = 13.1 × 10^–6^ cm/s and a *P*_app,B-A_ = 33.2 × 10^–6^ cm/s, thus suggesting its high permeability. Subsequently, *in vivo* time-dependent BBB-penetration studies over a time frame of 30 min were further carried out. In detail, compound **6** was subcutaneously injected to mice at a dose of 10 mg/kg, followed by collection of the brain tissue and blood samples at 5, 10, and 30 min. After plasma was obtained from the blood samples, the homogenate samples of both plasma and brain were tested to give the corresponding concentration of compound **6** via LC–MS/MS method, and then the brain-to-plasma ratios were calculated (Table 5). Compound **6** was detected with the highest concentration in the plasma at 5 min after injection, which was similar to our previous report.^20^ Sample concentrations of compound **6** in brain at 5, 10, and 30 min after administration were 0.187, 0.235, and 0.264 μg/g, respectively, revealing that compound **6** quickly penetrated into the brain after s.c. administration and remained in the brain for a long time. Although a 33% decrease in the plasma levels was observed over 30 min, a 43% increase in the concentration of compound **6** in the brain was observed. This resulted in an overall increase the brain-to-plasma ratio of compound **6** over time suggesting its continuing BBB-penetration. Several studies have reported that a concentration of 0.14–0.21 ug/g within 10 min of administration of drugs like morphine, naloxone and nalfurafine showed excellent correlation to their high potency.^56−59^ Thus, combined together, these results demonstrated that compound **6** was a central nervous system-penetrant agent.

**Table 5:** Time Dependent BBB Penetration of Compound 6 in Mice (n = 3, Mean ± SD)

Time (min)	5	10	30
Brain (μg/g)	0.187 ± 0.07	0.235 ± 0.06	0.264 ± 0.05
Plasma (μg/mL)	1.823 ± 0.28	1.36 ± 0.21	1.22 ± 0.33
Brain-to-Plasma ratio	0.102	0.173	0.217

### Drug-like Properties Assessment

To further characterize compound **6** (NTZ) as a promising lead, some key ADMET assays were carried out accordingly. Plasma protein binding of compound **6** (NTZ) was preferably moderate across species (44% for human, 60% for Sprague–Dawley rat). The metabolic stability of compound **6** was also evaluated in human and Sprague–Dawley rat liver microsomes, showing that it exhibited reasonable metabolic stability profiles in both species (both half-lives are >60 min) (Table S1 and S2). The cytochrome P450 (CYP) inhibition assay suggested that compound **6** (NTZ) did not show significant inhibitory effects up to 10 μM against a battery of CYP isozymes including CYP1A2, CYP2A6, CYP2C9, and CYP3A4 while its inhibitory effect on CYP2C19 warrants further investigation (Table S3). Bacterial reverse mutation assay indicated that no mutagenic potential of compound **6** (NTZ) was observed up to 100 μM when treated with typical tester strains. Of note, medications that have the human ether-a-go-go related gene (hERG)-related cardiotoxicity may trigger the blockade of rapid delayed rectifier voltage-gated potassium channel (I_Kr_), resulting in QT interval prolongation with potential risks of cardiac arrest.^60^ Thus, hERG liability studies have been a standard evaluation of any drug candidate to decide if they carry desirable cardiac safety properties for further development independent of their potential application in chronic or acute conditions. In the automated patch-clamp assay,^61^ compound **6** (NTZ) possessed a reasonable hERG inhibitory activity with an IC_50_ value of 7.4 μM, compared to the positive control E-4031 (IC_50_ = 29 nM) (Table S4). Since it is widely accepted that a desired lead is expected to have micromolar level of inhibitory effect against hERG channel, we thus reasoned that the chance of compound **6** (NTZ) causing sever cardiotoxicity could be manageable. Lastly, lipophilic efficiency (LipE) has long been proposed as a key metric that combines drug affinity and lipophilic-driven binding in the assessment of drug-likeness in lead optimization toward drug candidates.^62^ It turned out that compound **6** had a LipE value of 7.77, which well fell into the reportedly optimal values of LipE ranging from 5 to 10.^63^ In summary, these metrics obtained from ADMET assays demonstrated favorable drug-likeness and safety profiles of compound **6** (NTZ).

### Molecular Docking Studies

To further comprehend the plausible binding mode of compound **6** (NTZ) at the MOR in the context of its function, molecular docking studies were carried out and compared to the parent compound, NAT. Both NAT and compound **6** (NTZ) were docked in the antagonist bound conformation of the MOR (PDB ID 4DKL)^31^ using GOLD2020.^64^ The binding site was defined to encompass all atoms within 10 Å of the γ-carbon of D147^3.32^ along with a distance constraint between the 17-*N* atom of the ligands’ epoxymorphinan structure and the carboxylate group of D147^3.32^. Clustering of the docking poses based on root-mean-square deviation (RMSD) resulted in a single high scoring cluster family for both compounds with similar docking scores (ChemPLP) of 74.25 and 74.59 for NAT and compound **6** (NTZ) respectively (Figure S3).

**Figure 5:**
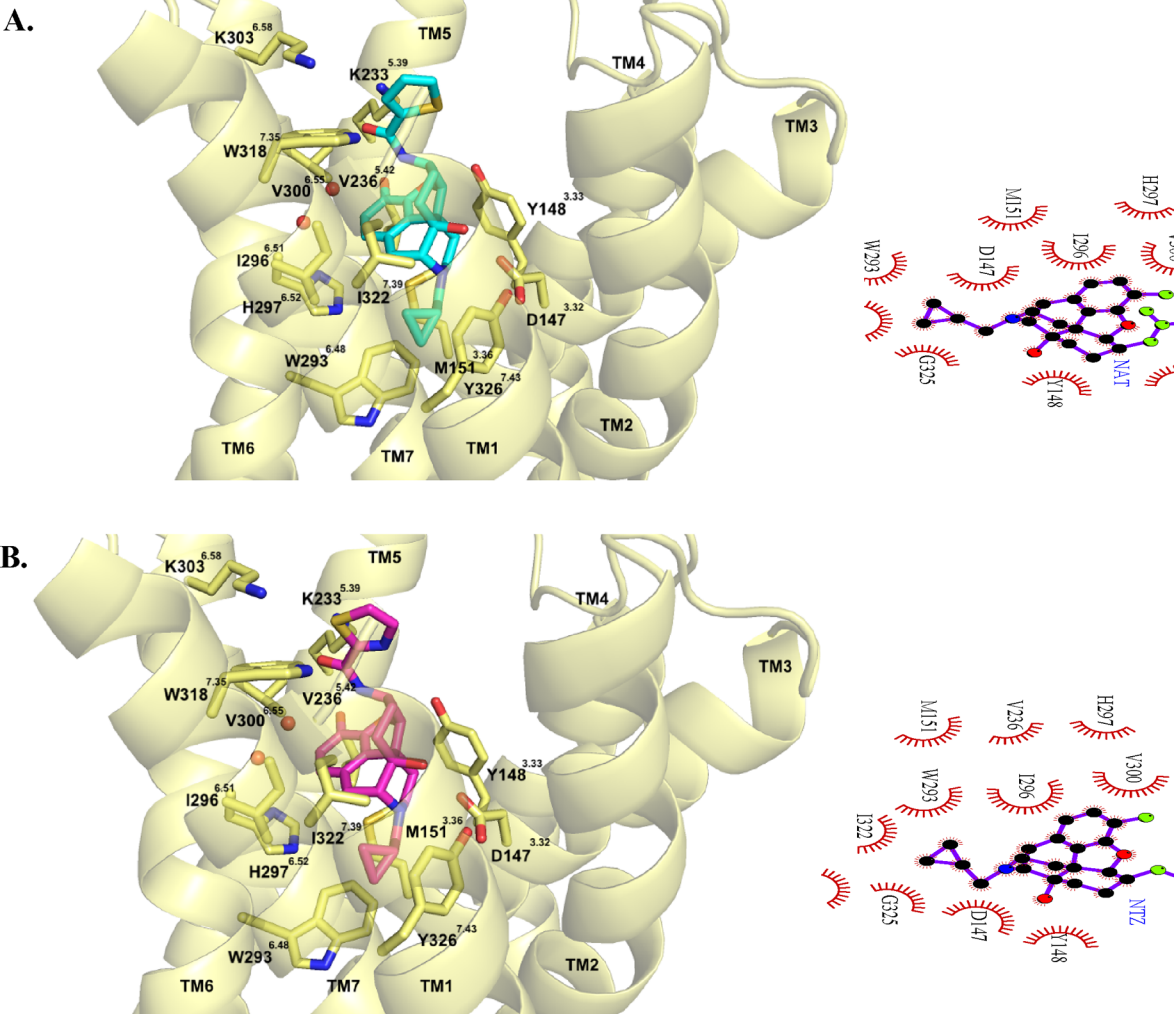
Binding modes of NAT (A) and compound **6** (NTZ) (B) at the MOR^inactive^ (PDB ID 4DKL). Protein is shown as cartoon model in light-yellow; NAT, NTZ, and important amino acid residues are shown as stick models. Carbon atoms: NAT (cyan); NTZ (magenta); key amino acid residues (yellow)). Oxygen atoms (red); nitrogen atoms (blue). Two-dimensional depictions of binding modes of NAT and NTZ, respectively, are also shown.

The docking results revealed that the epoxymorphinan moiety (message portion) of both NAT and compound 6 (NTZ) interacted with the MOR orthosteric binding site in the same manner as observed for other epoxymorphinan MOR ligands.^18,65^ The message portions formed ionic interactions with D147^3.32^, hydrogen bonding interactions with Y148^3.33^, water mediated hydrogen bonding interactions with H297^6.52^, and hydrophobic interactions with the residues M151^3.36^, W293^6.48^, I296^6.51^, H297^6.52^, I322^7.39^ and Y326^7.43^ (Figure 5). Meanwhile the thiophene and thiazole rings (address portions) of NAT and compound **6** (NTZ) respectively, showed similar displaced π stacking interactions with W318^7.35^ located in the allosteric binding site (Figure 5). In contrast to what we hypothesized, incorporation of the nitrogen atom to the thiophene ring of NAT did not result in additional hydrogen bonding interactions with K303^6.58^ for compound **6** (NTZ). Instead, it resulted in a flip in the orientation of the thiazole ring positioning the sulfur atom to now be involved in stabilizing the *S*–π interactions^66,67^ with W318^7.35^ (Figure 5B). These results provided explanation to the very similar MOR binding affinities (*K*_i_) and selectivities obtained in the radioligand binding studies.

## Conclusions

Developing highly potent and selective MOR modulators with favorable safety profiles and drug-like properties is highly desirable to address the dire opioid crisis. Our previous efforts have demonstrated that NAT, a thiophene ring containing epoxymorphinan derivative, acted as a potent CNS-acting MOR ligand both *in vivo* and *in vitro*. However, the further development of NAT was hampered by its significant withdrawal symptoms at higher doses. In light of the concept of activity cliff as well as our successful application of CNS MPO algorithm in discovering PNS-preferred MOR modulators, we decided to introduce a nitrogen atom into the thiophene ring of the original hit NAT to further explore SAR derived from NAT, with the hope to discover agents with maintained or enhanced pharmacological activities and BBB permeability while showing fewer withdrawal symptoms and improved safety profiles. Thus, a set of 16 target compounds was rationally designed, chemically synthesized and pharmacologically evaluated in this work. All the target compounds exhibited strong binding affinity at the MOR and good selectivity over the KOR and the DOR. Further pharmacological characterizations indicated that compound **6** (NTZ) exhibited comparable antagonism against DAMGO induced intracellular calcium flux and 10-fold higher potency of antagonistic effect to antagonize morphine’s antinociceptive activity as well as overall fewer withdrawal symptoms in comparison with the parent hit NAT. BBB penetration studies corroborated that compound **6** (NTZ) could penetrate BBB to exert its pharmacological effects as designed, indicating the potentially limited applicability of CNS MPO algorithm in drug design on CNS-acting epoxymorphinan derivatives which is in line with other studies showing sufficient BBB penetration with MPO scores <4. Lastly, compound **6** (NTZ) was demonstrated to possess a favorable ADMET profile by assessment of several fundamental drug-like properties. Collectively, our current study demonstrated the prominence of replacement of a −CH group with a nitrogen atom in heteroaromatic ring systems in search for structurally diverse and functionally effective modulators of opioid receptors. Further in-depth and extensive pharmacokinetics and pharmacodynamics studies, e.g. bioavailability and addiction potency, are under way to verify the lead compound status of NTZ. In summary, we have successfully identified and characterized compound **6** (NTZ) as a promising centrally efficacious MOR-targeted candidate that can potentially be employed in the development of novel treatments for OUD.

## Experimental Section

### Chemistry

All nonaqueous reactions were conducted under a dried nitrogen atmosphere. Solvents and reagents were obtained from either Combi-blocks, Sigma-Aldrich, or Enamine LLC, and were used as received. Analytical thin-layer chromatography (TLC) was performed on Analtech Uniplate F254 plates and flash column chromatography (FCC) used silica gel (230–400 mesh, Merck). ^1^H (400 MHz) and ^13^C (100 MHz) nuclear magnetic resonance (NMR) spectra were recorded on a Bruker Ultrashield 400 Plus spectrometer. Chemical shifts were expressed in δ units (ppm), using TMS as an internal standard (invisible in some NMR spectra as shown in the Supporting Information due to its extremely high volatility), and *J* values were reported in hertz (Hz). Mass spectra were obtained on an Applied BioSystems 3200 Q trap with a turbo V source for Turbolon Spray. Analytical reversed-phase high performance liquid chromatography (HPLC) was performed on a Waters Arc HPLC system using XBridge C_18_ 3.5 μm (4.6 × 50 mm) column. All analyses were conducted at ambient temperature with a flow rate of 1 mL/min. The mobile phase consisted of 0.1% formic acid in water (solvent A) and acetonitrile (solvent B). A gradient starting at 98% solvent A going to 2% in 10 min and equilibrating the column for 5 min was employed. The UV detector was set to 210 nm and the injection volume was 5 μL. The purities of final compounds were determined by calculating the percentage of the peak area corresponding to the analyzed compound, with retention times (Rt) reported in minutes. All newly synthesized compounds had a purity of ≥95%.

#### General Procedure for the Amide Coupling Reaction

A solution of carboxylic acid (2.1 equiv) in anhydrous DMF (1.5 mL) was added with *N*-(3- dimethylaminopropyl)-*N*′-ethylcarbodiimide hydrochloride (EDCI, 2.5 equiv), hydrobenzotriazole (HOBt, 2.5 equiv), triethylamine (TEA, 4 equiv) and 4 Å molecular sieves, on an ice–water bath. After 1 h, a solution of 6α- or 6β-naltrexamine (1 equiv) in anhydrous DMF (1.5 mL) was added dropwise. The resulting mixture was stirred at room temperature. Once TLC indicated the complete consumption of naltrexamine, the reaction mixture was diluted with methanol/dichloromethane and filtered through Celite. The filtrate was concentrated to dryness, dissolved in anhydrous methanol (5 mL), and K_2_CO_3_ (4 equiv) was added. The mixture was stirred overnight at room temperature and filtered again through Celite. After concentration, the residue was purified by column chromatography with methanol/dichloromethane (0.1% ammonia–water) as eluent to furnish the free base. Following structural confirmation via 1H NMR, the free base was converted into its hydrochloride salt, which was fully characterized using 1H NMR, 13C NMR, HRMS, and HPLC.

##### 17-Cyclopropylmethyl-3,14β-dihydroxy-4,5α-epoxy-6α-(5′-isothiazolylcarboxamido)-morphinan hydrochloride (1)

^1^H NMR (400 MHz, DMSO-*d*_6_) δ 9.25 (brs, 1H, exchangeable), 8.87 (brs, 1H, exchangeable), 8.72 (d, *J* = 7.6 Hz, 1H, exchangeable), 8.68 (d, *J* = 1.8 Hz, 1H), 8.13 (d, *J* = 1.8 Hz, 1H), 6.73 (d, *J* = 8.1 Hz, 1H), 6.59 (d, *J* = 8.1 Hz, 1H), 6.36 (brs, 1H, exchangeable), 4.77 (d, *J* = 3.8 Hz, 1H), 4.60 (m, 1H), 3.94 (d, *J* = 6.8 Hz, 1H), 3.26 (m, 2H), 3.08 (m, 2H), 2. 98 (m, 1H), 2.73 (m, 1H), 2.44 (m, 1H), 1.93 (m, 1H), 1.65 (dd, *J* = 13.0, 2.3 Hz, 1H), 1.54 (m, 1H), 1.46 (m, 1H), 1.20 (m, 1H), 1.10 (m, 1H), 0.70 (m, 1H), 0.62 (m, 1H), 0.51 (m, 1H), 0.41 (m, 1H). ^13^C NMR (100 MHz, DMSO-*d*_6_) δ 162.7, 159.1, 158.6, 146.1, 138.8, 128.5, 124.0, 122.1, 119.1, 118.3, 86.7, 69.3, 61.0, 57.0, 46.2, 45.2, 45.2, 30.2, 29.1, 23.4, 19.0, 5.6, 5.1, 2.5. ESI-HRMS calcd for C_24_H_28_N_3_O_4_S *m*/*z* [M + H]^+^ 454.1795, found 454.1798; calcd for C_24_H_27_N_3_NaO_4_S *m*/*z* [M + Na]^+^ 476.1614, found 476.1618. % Purity: 96.85. Rt: 5.2 min.

##### 17-Cyclopropylmethyl-3,14β-dihydroxy-4,5α-epoxy-6α-(5′-isothiazolylacetamido)-morphinan hydrochloride (2)

^1^H NMR (400 MHz, DMSO-*d*_6_) δ 8.87 (brs, 1H, exchangeable), 8.42 (d, *J* = 1.3 Hz, 1H), 8.33 (d, *J* = 7.8 Hz, 1H, exchangeable), 7.24 (s, 1H), 6.73 (d, *J* = 8.1 Hz, 1H), 6.56 (d, *J* = 8.1 Hz, 1H), 6.27 (brs, 1H, exchangeable), 4.62 (d, *J* = 3.8 Hz, 1H), 4.40 (m, 1H), 4.04 (s, 2H), 3.93 (d, *J* = 6.8 Hz, 1H), 3.31 (m, 2H), 3.04 (m, 2H), 2.96 (m, 1H), 2.70 (m, 1H), 2.45 (m, 1H), 1.91 (m, 1H), 1.62 (m, 1H), 1.41 (m, 2H), 1.08 (m, 1H), 0.99 (m, 1H), 0.68 (m, 1H), 0.60 (m, 1H), 0.49 (m, 1H), 0.40 (m, 1H). ^13^C NMR (100 MHz, DMSO-*d*_6_) δ 168.5, 161.8, 157.1, 146.5, 139.3, 129.2, 123.4, 122.5, 119.6, 118.8, 87.7, 69.8, 61.3, 57.4, 45.9, 45.6, 35.3, 30.6, 29.6, 23.9, 20.1, 8.8, 6.1, 5.6, 3.0. ESI-HRMS calcd for C_25_H_30_N_3_O_4_S *m*/*z* [M + H]^+^ 468.1952, found 468.1930. % Purity: 96.29. Rt: 5.1 min.

##### 17-Cyclopropylmethyl-3,14β-dihydroxy-4,5α-epoxy-6α-(5′-thiazolylcarboxamido)-morphinan hydrochloride (3)

^1^H NMR (400 MHz, DMSO-*d*_6_) δ 9.25 (s, 1H), 8.90 (brs, 1H, exchangeable), 8.69 (s, 1H), 8.53 (d, *J* = 7.6 Hz, 1H, exchangeable), 6.73 (d, *J* = 8.1 Hz, 1H), 6.58 (d, *J* = 8.1 Hz, 1H), 4.74 (d, *J* = 3.9 Hz, 1H), 4.58 (m, 1H), 3.96 (d, *J* = 6.7 Hz, 1H), 3.28 (m, 2H), 2.98 (m, 3H), 2.72 (m, 1H), 2.46 (m, 1H), 1.94 (m, 1H), 1.62 (m, 1H), 1.50 (m, 2H), 1.21 (m, 1H), 1.11 (m, 1H), 0.69 (m, 1H), 0.61 (m, 1H), 0.50 (m, 1H), 0.41 (m, 1H). ^13^C NMR (100 MHz, DMSO-*d*_6_) δ 159.4, 158.0, 146.1, 144.1, 138.8, 135.3, 128.6, 122.1, 119.1, 118.3, 86.9, 69.3, 60.9, 56.9, 46.1, 45.2 (2C), 30.2, 29.1, 23.4, 19.1, 5.6, 5.1, 2.5. ESI-HRMS calcd for C_24_H_28_N_3_O_4_S *m*/*z* [M + H]^+^ 454.1795, found 454.1773; calcd for C_24_H_27_N_3_NaO_4_S *m*/*z* [M + Na]^+^ 476.1614, found 476.1597. % Purity: 95.24. Rt: 5.0 min.

##### 17-Cyclopropylmethyl-3,14β-dihydroxy-4,5α-epoxy-6α-(5′-thiazolylacetamido)-morphinan hydrochloride (4)

^1^H NMR (400 MHz, DMSO-*d*_6_) δ 9.03 (s, 1H), 8.84 (s, 1H), 8.19 (d, *J* = 7.7 Hz, 1H), 7.76 (s, 1H), 6.73 (d, *J* = 8.0 Hz, 1H), 6.56 (d, *J* = 8.0 Hz, 1H), 4.59 (s, 1H), 4.39 (m, 1H), 3.90 (d, *J* = 6.2 Hz, 1H), 3.84 (s, 2H), 3.33 (m, 1H), 3.25 (m, 1H), 3.07 (m, 2H), 2.93 (m, 1H), 2.70 (m, 1H), 2.43 (m, 1H), 1.90 (m, 1H), 1.61 (m, 1H), 1.41 (m, 2H), 1.08 (m, 1H), 0.93 (m, 1H), 0.71 (m, 1H), 0.60 (m, 1H), 0.49 (m, 1H), 0.38 (m, 1H). ^13^C NMR (100 MHz, DMSO-*d*_6_) δ 168.1, 153.9, 145.9, 140.6, 138.8, 132.6, 128.7, 122.0, 119.1, 118.2, 87.2, 69.3, 61.0, 57.0, 56.0, 45.3, 45.19, 32.9, 30.1, 29.1, 23.4, 19.5, 5.6, 5.1, 2.5. ESI-HRMS calcd for C_25_H_30_N_3_O_4_S *m*/*z* [M + H]^+^ 468.1952, found: 468.1939. % Purity: 96.12. Rt: 4.8 min.

##### 17-Cyclopropylmethyl-3,14β-dihydroxy-4,5α-epoxy-6α-[(3′-(thiazolyl-5′′-yl)propan-amido]morphinan hydrochloride (5)

^1^H NMR (400 MHz, DMSO-*d*_6_) δ 8.95 (s, 1H), 8.82 (brs, 1H), 7.77 (d, *J* = 8.0 Hz, 1H), 7.69 (s, 1H), 6.71 (d, *J* = 8.1 Hz, 1H), 6.55 (d, *J* = 8.1 Hz, 1H), 4.57 (d, *J* = 3.8 Hz, 1H), 4.41 (m, 1H), 3.88 (d, *J* = 6.5 Hz, 1H), 3.32 (m, 1H), 3.25 (m, 1H), 3.09 (t, *J* = 7.4 Hz, 2H), 3.03 (m, 2H), 2.94 (m, 1H), 2.71 (m, 1H), 2.54 (d, *J* = 7.3 Hz, 2H), 2.44 (m, 1H), 1.85 (m, 1H), 1.60 (m, 1H), 1.38 (m, 2H), 1.04 (m, 1H), 0.93 (m, 1H), 0.69 (m, 1H), 0.61 (m, 1H), 0.48 (m, 1H), 0.40 (m, 1H). ^13^C NMR (100 MHz, DMSO-*d*_6_) δ 170.1, 152.8, 145.9, 140.1, 138.7, 138.3, 128.6, 122.0, 119.1, 118.2, 87.4, 69.3, 61.0, 57.0, 54.8, 45.1, 44.9, 36.4, 30.1, 29.1, 23.4, 22.1, 19.6, 5.6, 5.1, 2.5. ESI-HRMS calcd for C_26_H_32_N_3_O_4_S *m*/*z* [M + H]^+^ 482.2108, found: 482.2091. % Purity: 96.92. Rt: 4.9 min.

##### 17-Cyclopropylmethyl-3,14β-dihydroxy-4,5α-epoxy-6α-(2′-thiazolylcarboxamido)-morphinan hydrochloride (6)

^1^H NMR (400 MHz, DMSO-*d*_6_) δ 9.34 (s, 1H, exchangeable), 8.83 (brs, 1H, exchangeable), 8.10 (m, 2H, including an exchangeable proton), 8.05 (d, *J* = 3.1 Hz, 1H), 6.73 (d, *J* = 8.1 Hz, 1H), 6.60 (d, *J* = 8.1 Hz, 1H), 6.27 (s, 1H, exchangeable), 4.77 (d, *J* = 3.8 Hz, 1H), 4.60 (m, 1H), 3.89 (dd, *J* = 6.8 Hz, 0.44 Hz, 1H), 3.39 (m, 1H), 3.28 (m, 1H), 3.10 (m, 2H), 2.94 (m, 1H), 2.73 (m, 1H), 2.44 (m, 1H), 1.90 (m, 1H), 1.65 (m, 1H), 1.60 (m, 1H), 1.45 (m, 1H), 1.09 (t, *J* = 7.0 Hz, 1H), 1.05 (m, 1H), 0.70 (m, 1H), 0.61 (m, 1H), 0.49 (m, 1H), 0.40 (m, 1H). ^13^C NMR (100 MHz, DMSO-*d*_6_) δ 163.1, 158.3, 145.7, 143.9, 138.9, 128.6, 126.2, 122.0, 119.4, 118.3, 87.2, 69.2, 60.9, 57.0, 45.6, 45.3, 45.1, 30.1, 29.1, 23.4, 19.7, 5.6, 5.1, 2.5. ESI-HRMS calcd for C_24_H_28_N_3_O_4_S *m*/*z* [M + H]^+^ 454.1795, found 454.1796; calcd for C_24_H_27_N_3_NaO_4_S *m*/*z* [M + Na]^+^ 476.1614, found 476.1603. % Purity: 100. Rt: 5.3 min.

##### 17-Cyclopropylmethyl-3,14β-dihydroxy-4,5α-epoxy-6α-(2′-thiazolylacetamido)-morphinan hydrochloride (7)

^1^H NMR (400 MHz, DMSO-*d*_6_) δ 8.82 (s, 1H), 8.25 (d, *J* = 7.9 Hz, 1H), 7.73 (d, *J* = 3.1 Hz, 1H), 7.63 (d, *J* = 3.2 Hz, 1H), 6.72 (d, *J* = 8.0 Hz, 1H), 6.56 (d, *J* = 8.1 Hz, 1H), 6.26 (brs, 1H), 4.61 (d, *J* = 3.6 Hz, 1H), 4.44 (m, 1H), 4.02 (s, 2H), 3.90 (d, *J* = 6.7 Hz, 1H), 3.33 (m, 1H), 3.24 (m, 1H), 3.06 (m, 2H), 2.94 (m, 1H), 2.71 (m, 1H), 2.44 (m, 1H), 1.86 (m, 1H), 1.61 (m, 1H), 1.41 (m, 2H), 1.05 (m, 1H), 0.98 (m, 1H), 0.71 (m, 1H), 0.60 (m, 1H), 0.49 (m, 1H), 0.39 (m, 1H). ^13^C NMR (100 MHz, DMSO-*d*_6_) δ 167.1, 163.8, 145.9, 141.2, 138.8, 128.6, 122.0, 120.5, 119.1, 118.2, 87.2, 69.2, 61.0, 57.0, 45.4, 45.3, 45.1, 30.1, 29.1, 23.4, 19.5, 8.4, 5.6, 5.1, 2.5. ESI-HRMS calcd for C_25_H_30_N_3_O_4_S *m*/*z* [M + H]^+^ 468.1952, found: 468.1948. % Purity: 97.46. Rt: 5.0 min.

##### 17-Cyclopropylmethyl-3,14β-dihydroxy-4,5α-epoxy-6α-[(3′-(thiazolyl-2′′-yl)propan-amido]morphinan hydrochloride (8)

^1^H NMR (400 MHz, DMSO-*d*_6_) δ 8.84 (s, 1H), 7.83 (d, *J* = 8.0 Hz, 1H), 7.72 (d, *J* = 3.2 Hz, 1H), 7.60 (d, *J* = 3.2 Hz, 1H), 6.71 (d, *J* = 8.0 Hz, 1H), 6.55 (d, *J* = 8.0 Hz, 1H), 4.57 (d, *J* = 3.4 Hz, 1H), 4.40 (m, 1H), 3.90 (d, *J* = 6.4 Hz, 1H), 3.29 (m, 2H), 3.23 (t, *J* = 7.0 Hz, 2H), 3.05 (m, 2H), 2.94 (m, 1H), 2.72 (m, 1H), 2.66 (t, *J* = 7.2 Hz, 2H), 2.42 (m, 1H), 1.84 (m, 1H), 1.60 (m, 1H), 1.40 (m, 2H), 1.06 (m, 1H), 0.94 (m, 1H), 0.70 (m, 1H), 0.61 (m, 1H), 0.47 (m, 1H), 0.38 (m, 1H). ^13^C NMR (100 MHz, DMSO-*d*_6_) δ 170.0, 169.81, 145.9, 141.2, 138.8, 128.7, 122.1, 119.7, 119.1, 118.2, 87.4, 69.3, 61.0, 57.0, 54.9, 45.1, 44.9, 34.5, 30.6, 30.1, 29.1, 28.1, 23.4, 5.6, 5.1, 2.5. ESI-HRMS calcd for C_26_H_32_N_3_O_4_S *m*/*z* [M + H]^+^ 482.2108, found: 482.2107. % Purity: 98.51. Rt: 4.9 min.

##### 17-Cyclopropylmethyl-3,14β-dihydroxy-4,5α-epoxy-6β-(5′-isothiazolylcarboxamido)-morphinan hydrochloride (9)

^1^H NMR (400 MHz, DMSO-*d*_6_) δ 9.36 (brs, 1H, exchangeable), 9.26 (d, *J* = 8.1 Hz, 1H, exchangeable), 8.90 (brs, 1H, exchangeable), 8.68 (d, *J* = 1.8 Hz, 1H), 8.05 (d, *J* = 1.8 Hz, 1H), 6.74 (d, *J* = 8.1 Hz, 1H), 6.66 (d, *J* = 8.1 Hz, 1H), 6.29 (brs, 1H, exchangeable), 4.82 (d, *J* = 7.8 Hz, 1H), 3.91 (d, *J* = 5.5 Hz, 1H), 3.62 (m, 1H), 3.32 (m, 2H), 3.10 (m, 2H), 2.89 (m, 1H), 2.43 (m, 2H), 1.91 (m, 1H), 1.81 (m, 1H), 1.61 (m, 1H), 1.47 (d, *J* = 9.1 Hz, 1H), 1.40 (m, 1H), 1.11 (m, 1H), 0.68 (m, 1H), 0.61 (m, 1H), 0.51 (m, 1H), 0.42 (m, 1H). ^13^C NMR (100 MHz, DMSO-*d*_6_) δ 163.0, 159.1, 158.5, 141.9, 141.3, 129.5, 123.4, 120.6, 119.4, 117.9, 89.4, 69.6, 61.6, 56.7, 51.2, 46.4, 45.6, 29.2, 27.2, 23.5, 23.0, 5.7, 5.1, 2.6. ESI-HRMS calcd for C_24_H_28_N_3_O_4_S *m*/*z* [M + H]^+^ 454.1795, found 454.1742. % Purity: 97.84. Rt: 5.2 min.

##### 17-Cyclopropylmethyl-3,14β-dihydroxy-4,5α-epoxy-6β-(5′-isothiazolylacetamido)-morphinan hydrochloride (10)

^1^H NMR (400 MHz, DMSO-*d*_6_) δ 8.87 (brs, 1H, exchangeable), 8.71 (d, *J* = 7.8 Hz, 1H, exchangeable), 8.42 (d, *J* = 1.6 Hz, 1H), 7.24 (d, *J* = 1.3 Hz, 1H), 6.73 (d, *J* = 8.1 Hz, 1H), 6.64 (d, *J* = 8.2 Hz, 1H), 6.30 (brs, 1H, exchangeable), 4.60 (d, *J* = 7.8 Hz, 1H), 3.97 (s, 2H), 3.87 (m, 2H), 3.30 (m, 2H), 3.07 (m, 2H), 2.81 (m, 1H), 2.41 (m, 2H), 1.80 (m, 2H), 1.53 (m, 1H), 1.43 (d, *J* = 9.0 Hz, 1H), 1.32 (m, 1H), 1.05 (m, 1H), 0.68 (m, 1H), 0.60 (m, 1H), 0.51 (m, 1H), 0.41 (m, 1H). ^13^C NMR (100 MHz, DMSO-*d*_6_) δ 168.6, 161.5, 157.1, 142.5, 141.8, 130.0, 123.5, 121.1, 119.8, 118.4, 90.1, 70.1, 65.3, 57.1, 51.6, 46.9, 46.1, 35.6, 29.7, 27.7, 24.0, 23.4, 6.2, 5.6, 3.1. ESI-HRMS calcd for C_25_H_30_N_3_O_4_S *m*/*z* [M + H]^+^ 468.1952, found 468.1935. % Purity: 99.29. Rt: 4.9 min.

##### 17-Cyclopropylmethyl-3,14β-dihydroxy-4,5α-epoxy-6β-(5′-thiazolylcarboxamido)-morphinan hydrochloride (11)

^1^H NMR (400 MHz, DMSO-*d*_6_) δ 9.25 (s, 1H), 9.03 (d, *J* = 8.1 Hz, 1H, exchangeable), 8.90 (brs, 1H, exchangeable), 8.58 (s, 1H), 6.74 (d, *J* = 8.1 Hz, 1H), 6.66 (d, *J* = 8.2 Hz, 1H), 6.26 (brs, 1H, exchangeable), 4.80 (d, *J* = 7.8 Hz, 1H), 3.90 (d, *J* = 5.0 Hz, 1H), 3.63 (m, 1H), 3.33 (m, 2H), 3.10 (m, 2H), 2.88 (m, 1H), 2.42 (m, 2H), 1.91 (m, 1H), 1.80 (m, 1H), 1.58 (m, 1H), 1.47 (d, *J* = 9.0 Hz, 1H), 1.40 (m, 1H), 1.08 (m, 1H), 0.69 (m, 1H), 0.61 (m, 1H), 0.51 (m, 1H), 0.42 (m, 1H). ^13^C NMR (100 MHz, DMSO-*d*_6_) δ 159.3, 158.1, 143.5, 142.0, 141.3, 135.7, 129.5, 120.6, 119.3, 117.9, 89.6, 69.6, 61.5, 56.6, 51.2, 46.4, 45.6, 29.3, 27.3, 23.7, 23.0, 5.7, 5.1, 2.6. ESI-HRMS calcd for C_24_H_28_N_3_O_4_S *m*/*z* [M + H]^+^ 454.1795, found 454.1802; calcd for C_24_H_27_N_3_NaO_4_S *m*/*z* [M + Na]^+^ 476.1614, found 476.1620. % Purity: 98.74. Rt: 4.8 min.

##### 17-Cyclopropylmethyl-3,14β-dihydroxy-4,5α-epoxy-6β-(5′-thiazolylacetamido)-morphinan hydrochloride (12)

^1^H NMR (400 MHz, DMSO-*d*_6_) δ 9.00 (s, 1H), 8.82 (s, 1H), 8.53 (d, *J* = 7.6 Hz, 1H), 7.74 (s, 1H), 6.71 (d, *J* = 7.9 Hz, 1H), 6.64 (d, *J* = 8.0 Hz, 1H), 6.17 (brs, 1H), 4.57 (d, *J* = 7.6 Hz, 1H), 3.80 (m, 1H), 3.76 (s, 2H), 3.31 (m, 2H), 3.06 (m, 2H), 2.81 (m, 1H), 2.41 (m, 2H), 1.70 (m, 2H), 1.52 (m, 1H), 1.43 (m, 1H), 1.34 (m, 1H), 1.07 (m, 2H), 0.62 (m, 1H), 0.53 (m, 1H), 0.50 (m, 1H), 0.38 (m, 1H). ^13^C NMR (100 MHz, DMSO-*d*_6_) δ 168.2, 153.9, 142.0, 141.2, 141.0, 132.2, 129.5, 120.5, 117.9, 89.7, 69.6, 61.6, 56.6, 54.8, 51.0, 46.4, 45.5, 33.3, 29.2, 27.2, 23.4, 22.9, 5.6, 5.1, 2.5. ESI-HRMS calcd for C_25_H_30_N_3_O_4_S *m*/*z* [M + H]^+^ 468.1952, found: 468.1948. % Purity: 98.12. Rt: 5.2 min.

##### 17-Cyclopropylmethyl-3,14β-dihydroxy-4,5α-epoxy-6β-[(3′-(thiazolyl-5′′-yl)propan-amido]morphinan hydrochloride (13)

^1^H NMR (400 MHz, DMSO-*d*_6_) δ 8.94 (s, 1H), 8.81 (s, 1H), 8.21 (d, *J* = 7.8 Hz, 1H), 7.67 (s, 1H), 6.72 (d, *J* = 8.1 Hz, 1H), 6.64 (d, *J* = 8.2 Hz, 1H), 6.17 (brs, 1H), 4.52 (d, *J* = 7.8 Hz, 1H), 3.83 (d, *J* = 5.2 Hz, 1H), 3.40 (m, 1H), 3.31 (m, 2H), 3.07 (m, 4H), 2.83 (m, 1H), 2.41 (m, 4H), 1.68 (m, 2H), 1.50 (m, 1H), 1.41 (m, 1H), 1.32 (m, 1H), 1.08 (m, 1H), 0.68 (m, 1H), 0.59 (m, 1H), 0.50 (m, 1H), 0.40 (m, 1H). ^13^C NMR (100 MHz, DMSO-*d*_6_) δ 170.2, 152.8, 142.0, 141.2, 140.1, 138.1, 129.6, 120.5, 119.2, 117.9, 89.7, 69.6, 61.6, 56.6, 50.7, 46.4, 45.5, 36.9, 32.4, 29.2, 27.2, 22.9, 22.0, 5.6, 5.1, 2.5. ESI-HRMS calcd for C_26_H_32_N_3_O_4_S *m*/*z* [M + H]^+^ 482.2108, found: 482.2097. % Purity: 96.02. Rt: 4.8 min.

##### 17-Cyclopropylmethyl-3,14β-dihydroxy-4,5α-epoxy-6β-(2′-thiazolylcarboxamido)-morphinan hydrochloride (14)

^1^H NMR (400 MHz, DMSO-*d*_6_) δ 9.08 (d, *J* = 8.6 Hz, 1H), 8.90 (brs, 1H, exchangeable), 8.07 (d, *J* = 3.1 Hz, 1H), 8.05 (d, *J* = 3.1 Hz, 1H), 6.73 (d, *J* = 8.1 Hz, 1H), 6.65 (d, *J* = 8.2 Hz, 1H), 6.25 (brs, 1H, exchangeable), 4.98 (d, *J* = 7.8 Hz, 1H), 3.89 (d, *J* = 5.2 Hz, 1H), 3.67 (m, 1H), 3.30 (m, 2H), 3.09 (m, 2H), 2.88 (m, 1H), 2.42 (m, 2H), 2.01 (m, 1H), 1.81 (m, 1H), 1.52 (m, 1H), 1.41 (m, 2H), 1.11 (m, 1H), 0.68 (m, 1H), 0.59 (m, 1H), 0.51 (m, 1H), 0.41 (m, 1H). ^13^C NMR (100 MHz, DMSO-*d*_6_) δ 163.7, 158.8, 143.8, 142.1, 141.3, 129.6, 125.8, 120.6, 119.2, 117.8, 89.4, 69.6, 61.5, 56.6, 51.1, 46.4, 45.6, 29.4, 27.2, 23.5, 23.0, 5.7, 5.1, 2.6. ESI-HRMS calcd for C_24_H_28_N_3_O_4_S *m*/*z* [M + H]^+^ 454.1795, found 454.1782. % Purity: 98.25. Rt: 5.2 min.

##### 17-Cyclopropylmethyl-3,14β-dihydroxy-4,5α-epoxy-6β-(2′-thiazolylacetamido)-morphinan hydrochloride (15)

^1^H NMR (400 MHz, DMSO-*d*_6_) δ 8.82 (s, 1H), 8.61 (d, *J* = 7.8 Hz, 1H), 7.73 (d, *J* = 3.3 Hz, 1H), 7.63 (d, *J* = 3.2 Hz, 1H), 6.71 (d, *J* = 8.0 Hz, 1H), 6.64 (d, *J* = 8.2 Hz, 1H), 4.58 (d, *J* = 7.8 Hz, 1H), 3.94 (s, 2H), 3.84 (d, *J* = 5.5 Hz, 1H), 3.31 (m, 3H), 3.04 (m, 2H), 2.87 (m, 1H), 2.41 (m, 2H), 1.73 (m, 2H), 1.55 (m, 1H), 1.44 (m, 1H), 1.32 (m, 1H), 1.10 (m, 1H), 0.68 (m, 1H), 0.60 (m, 1H), 0.51 (m, 1H), 0.41 (m, 1H). ^13^C NMR (100 MHz, DMSO-*d*_6_) δ 167.2, 163.6, 142.0, 141.3, 129.5, 120.6, 120.5, 119.3, 119.3, 117.9, 89.6, 69.6, 61.6, 56.6, 54.8, 51.1, 46.4, 45.5, 29.2, 27.2, 23.4, 22.9, 5.6, 5.1, 2.5. ESI-HRMS calcd for C_25_H_30_N_3_O_4_S *m*/*z* [M + H]^+^ 468.1952, found: 468.1935. % Purity: 97.01. Rt: 5.0 min.

##### 17-Cyclopropylmethyl-3,14β-dihydroxy-4,5α-epoxy-6β-[(3′-(thiazolyl-2′′-yl)propan-amido]morphinan hydrochloride (16)

^1^H NMR (400 MHz, DMSO-*d*_6_) δ 8.83 (s, 1H), 8.26 (d, *J* = 7.5 Hz, 1H), 7.72 (s, 1H), 7.59 (s, 1H), 6.72 (d, *J* = 8.1 Hz, 1H), 6.63 (d, *J* = 8.0 Hz, 1H), 4.54 (d, *J* = 7.7 Hz, 1H), 3.84 (s, 1H), 3.41 (m, 1H), 3.31 (m, 2H), 3.22 (m, 2H), 3.05 (m, 2H), 2.85 (m, 1H), 2.59 (t, *J* = 7.2 Hz, 2H), 2.41 (m, 2H), 1.72 (m, 2H), 1.50 (m, 1H), 1.41 (m, 1H), 1.32 (m, 1H), 1.05 (m, 1H), 0.69 (m, 1H), 0.58 (m, 1H), 0.50 (m, 1H), 0.40 (m, 1H). ^13^C NMR (100 MHz, DMSO-*d*_6_) δ 170.1, 169.5, 142.1, 141.3, 141.2, 129.6, 120.5, 119.6, 119.2, 117.8, 89.8, 69.6, 61.6, 56.6, 50.7, 46.4, 45.5, 34.8, 29.2, 28.1, 27.2, 23.6, 22.9, 5.7, 5.1, 2.6. ESI-HRMS calcd for C_26_H_32_N_3_O_4_S *m*/*z* [M + H]^+^ 482.2108, found: 482.2122. % Purity: 97.25. Rt: 4.9 min.

### Biological Evaluations

*Drugs.* Morphine (morphine sulfate pentahydrate) was obtained from Mallinckrodt (St. Louis, MO) or supplied by the National Institute on Drug Abuse (NIDA). The free base of naltrexone was supplied by the NIDA Drug Supply Program. All drugs and test compounds were dissolved in sterile-filtered distilled/deionized water. Other reagents and radioligands were sourced from Sigma-Aldrich or Thermo Fisher.

*Animals.* Male Swiss Webster mice (25–35 g, 6–8 weeks old, Harlan Laboratories, Indianapolis, IN) were housed in a temperature-controlled (20–22 °C) AAALAC-accredited facility with ad libitum access to food and water. The mice were kept on a 12-h light/12-h dark cycle (0600–1800 lights on) throughout the experiment and were tested during the light segment. Upon arrival at the vivarium, the mice were housed in groups of four per cage. After 1 week of habituation, the mice were moved to individual cages and given at least 24 h to acclimate. Mice were then randomly assigned to different treatment groups before the studies began. Experimenters were blinded to the treatment groups during the experiment and data analysis. No adverse events were observed during the experiment, and no mice were excluded from the analysis. Protocols and procedures (Animal Welfare Assurance Number D16–00180) were approved by the Institutional Animal Care and Use Committee (IACUC) at the Virginia Commonwealth University Medical Center and followed the recommendations of the International Association for the Study of Pain (IASP).

#### In Vitro Competitive Radioligand Binding Assay

The competition binding assay was performed using monoclonal mouse opioid receptors expressed in CHO cell lines (monoclonal human δ opioid receptor was used for the DOR assay). In this assay, 30 μg of membrane protein was incubated with the appropriate radioligand and varying concentrations of test compounds in TME buffer (50 mM Tris, 3 mM MgCl2, and 0.2 mM EGTA, pH 7.4) for 1.5 h at 30 °C. For radioligands, 1.3 nM [3H]naloxone (for mMOR-CHO; *K*_d_ = 1.45 nM), 0.25 nM [3H]diprenorphine (for mKOR-CHO; *K*_d_ = 0.27 nM) or 1 nM [3H]diprenorphine (for hDOR-CHO; *K*_d_ = 1.45 nM) were used.

The bound radioligand was separated using a Brandel harvester. Specific binding to the MOR, KOR, and DOR was calculated by subtracting the binding observed in the presence of 5 μM naltrexone, U50488, and SNC80, respectively, from the binding in their absence. Relative affinity values (IC50) were determined by fitting displacement binding inhibition data to a nonlinear regression model using GraphPad Prism 8.0 (GraphPad Software, San Diego, CA). The % inhibition was calculated using the formula: % inhibition = 100% - [(binding with the tested compound - nonspecific binding)/specific binding] × 100%. The IC_50_ values were converted to *K*_i_ values using the Cheng-Prusoff equation: *K*_i_ = IC_50_/ [1 + ([L*]/*K*_D_)], where [L*] is the concentration of the radioligand and *K*_D_ is the *K*_D_ of the radioligand.^68^

#### In Vitro [^35^S]GTPγS Functional Assay

The [^35^S]GTPγS functional assay was used to assess the efficacy of compounds at the MOR, KOR, and DOR. In this assay, 10 μg of MOR-CHO/KOR-CHO/DOR-CHO membrane protein was incubated in 500 μL of TME with 100 mM NaCl, 20 μM GDP, 0.1 nM [^35^S]GTPγS, and varying concentrations of the compound for 1.5 h in a 30 °C water bath. Protein concentration was adjusted using the Bradford protein assay. Nonspecific binding was measured with 20 μM unlabeled GTPγS, and 3 μM DAMGO/U50488H/SNC80 were used as full agonists for the respective receptors. After incubation, bound radioactivity was separated by filtration through GF/B glass fiber filters and measured by liquid scintillation counting. All assays were performed in duplicate and repeated at least three times. Net stimulated [35S]GTPγS binding was calculated as agonist-stimulated minus basal binding. Percent stimulation was calculated as (net-stimulated binding by ligand/net-stimulated binding by 3 μM DAMGO/U50488H/SNC80) × 100%.

#### Warm-Water Tail Immersion Assay

The antinociceptive effect of newly synthesized compounds was evaluated using the warm-water tail immersion assay. Swiss Webster mice (6 male mice per group, 25–35 g, 6–8 weeks old) were used, with the water bath maintained at 56 ± 0.1 °C. Baseline latency (control) was measured before compound administration, with only mice showing a baseline latency of 2–4 s included. In the agonism study, compounds were injected subcutaneously (s.c.) and the tail immersion test was performed 20 min later, with a 10 s maximum cutoff to prevent tissue damage. Antinociception was calculated as % MPE = [(test–control latency)/(10–control latency)] × 100. For antagonist effects, compounds were injected 5 min before morphine, and the test was conducted 20 min after morphine administration. AD50 values were calculated using least-squares regression and the Bliss method for 95% confidence intervals.

#### MOR Calcium Flux Assay

Ligands were initially tested at various concentrations (0.3 nM to 3 μM) for potential agonist activity in MOR-CHO cells. These cells were transfected with G_qi5_ pcDNA1 using Lipofectamine 2000 (Invitrogen) following the manufacturer’s protocol. After a 4-h incubation at 37 °C with 5% CO_2_, the cells were trypsinized, transferred to a clear black 96-well plate (Greiner Bio-one) at a density of 3 × 10^6^ cells per well, and incubated until confluency. Forty-eight hours post-transfection, the growth media was removed, and cells were incubated for 45 min with 50 μL of fluo-4 AM loading buffer [24 μL of 2 mM fluo-4 AM (Invitrogen), 12 μL of 250 mM probenecid in 6 mL assay buffer (HBSS-HEPES–Ca-Mg-probenecid)]. Afterward, the loading buffer was discarded, and the cells were incubated for an additional 15 min with 20 μL of each compound at varying concentrations and 60 μL assay buffer. Calcium concentrations were measured for 90 s following the addition of 20 μL of the agonist DAMGO using a microplate reader (FlexStation3, Molecular Devices). Peak values were obtained using SoftMaxPro software (Molecular Devices), and IC_50_ values were calculated by generating nonlinear regression curves using GraphPad Prism. Each dose was tested in triplicates, and the experiment was repeated at least four times to calculate standard errors.

#### In Vivo Opioid Withdrawal Studies

Swiss Webster mice (six males per group, weighing 25–35 g and aged 6–8 weeks) were used. Following a previously established protocol,^20^ a 75 mg morphine pellet was implanted subcutaneously into the back of each mouse, and they were allowed to recover in their home cages. Prior to testing, the mice were habituated for 30 min in an open-topped, clear Plexiglas observation chamber (26 × 26 × 26 cm^3^) with lines marking the floor into quadrants. Drugs and test compounds were administered subcutaneously, and withdrawal was precipitated 72 h postimplantation using naloxone (1 mg/kg, s.c.) or varying doses of test compounds. Withdrawal symptoms began within 3 min of antagonist administration. Escape jumps, paw tremors, and wet dog shakes were recorded by counting their occurrences over a 20 min period for each mouse. Results are expressed as the mean ± SEM.

#### Caco-2 Permeability Studies

Caco-2 cells (HTB-37) were cultured in T75 flasks using DMEM supplemented with 10% FBS, 1% glutamine, penicillin, and streptomycin at 37 °C and 5% CO2. Cells were passaged at 80–90% confluency and seeded into Millipore 96-well plates (25,000 cells/well) to form monolayers, with media changed every 2–3 days for 21 days until 100% confluency. Permeability studies involved adding 10 μM compound 6 to the apical (A) or basolateral (B) side and measuring permeation across the monolayer. Lucifer yellow dye (100 μM) was used in the A-side buffer as a control. Incubation lasted 1 h, and permeability was assessed using ranitidine/colchicine (low permeability) and labetalol/propranolol (high permeability) as controls. Samples were collected, quenched with methanol, centrifuged, and analyzed by HPLC-MS/MS to determine peak area ratios.

Data was expressed as
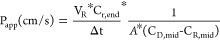
here V_R_ is the volume of the receiver chamber. C_R,end_ is the concentration of the test compound in the receiver chamber at the final time point, Δt denotes the incubation time, and A is the surface area of the cell monolayer. C_D,mid_ is the average midpoint concentration of the test compound on the donor side, calculated as the mean of the donor concentrations at 0 min and at the end time point. Similarly, C_R,mid_ is the midpoint concentration of the test compound on the receiver side, equal to half of the receiver concentration at the final time point. Test compound concentrations were determined based on their peak areas.

#### In Vivo BBB-Penetration Studies

Swiss Webster mice (three mice each time point) were administered compound **6** (10 mg/kg, s.c.) or vehicle. At 5-, 10-, and 30 min time points post administration, the mice were euthanized by decapitation, and blood and brain samples were collected. Blood samples were centrifuged at 15,000 g for 10 min at 4 °C to isolate plasma. Brains were washed twice with saline before harvesting and stored in saline until homogenization. Both brain and plasma samples were kept at −80 °C until further analysis.

#### LCMS/MS Analysis

Compound 6 was identified and quantified in mouse plasma and brain using a modified version of a previously established method, with naloxone-*d*_*5*_ as the internal standard.^20^ In brief, compound **6** was extracted from both blood and brain using naloxone-*d*_5_ as the internal standard (ISTD) by a liquid/liquid extraction. Before extraction, brain tissue samples were homogenized at a ratio of one-part tissue to three-parts water. Seven-point calibration curves (10–1000 ng/mL or ng/g) were prepared for in plasma, and included drug free control, negative control (lacking internal standard, ISTD) for plasma and brain, and quality control samples in plasma and brain (30, 300, and 750 ng/mL or ng/g). Naltrexone-*d*_5_, used as the internal standard, was added at a concentration of 10 ng/mL to either 100 μL of blood or 400 μL for brain homogenate to each calibrator, control, or sample, excluding the negative control. To these samples 0.5 mL of saturated carbonate/bicarbonate buffer (1:1, pH 9.5) and 2.0 mL of chloroform: isopropanol (8:2) were added. The mixtures were vortexed, centrifuged and the top aqueous layer was removed. The organic layer was transferred to a clean test tube and evaporated to dryness under nitrogen. The residues were reconstituted in 80:20 methanol: water and transferred to autosampler vials for analysis. Chromatographic separation of compound **6** and naltrexone-*d*_5_ was performed using a Shimadzu Nexera X2 liquid chromatography system with a Zorbax XDB-C18 4.6 × 75 mm, 3.5-μm column (Agilent Technologies, Santa Clara, CA). The mobile phase consisted of water with 1 g/L ammonium formate and 0.1% formic acid (Phase A) and methanol (Phase B). The flow rate was maintained at 1 mL/min. The gradient started at 20% B, increased to 80% within 1 min, and was held for 1.5 min before returning to the initial conditions. Detection was carried out using a Sciex 6500 QTRAP system with an IonDrive Turbo V source for TurbolonSpray (Sciex, Ontario, Canada). The curtain gas flow rate set to 30 mL/min while the ion source gases 1 and 2 were set to 60 mL/min. The source temperature was maintained at 650 °C and the ionspray voltage was set at 5500 V. The declustering potential was 58 eV. The following quantification and qualifying transition ions were monitored in positive multiple reaction monitoring (MRM) mode with collisions energies in parentheses: compound **6** 455> 437 (26), 455 > 268 & (41); naloxone-*d*_5_ 333> 212 (45), &333 > 315 (25). Retention times of compound **6** and naloxone-*d*_5_ were 1.81 and 1.69 min, respectively. The total run time was 4 min. Compound **6** concentration was determined by linear regression plot based on peak area ratios of the calibrators.

#### ADMET Assessment

The ADMET assessment including the hERG inhibitory activity, plasma protein binding, *in vitro* metabolic stability profile, CYP inhibitory effect and mutagenic evaluation were conducted by Eurofins Panlabs, Inc.^21,61,69−71^

#### hERG Inhibitory Activity Assay

Inhibition of the hERG potassium channel was assessed using CHO-K1 (Chinese Hamster Ovary) cells stably expressing hERG cDNA. Borosilicate glass micropipettes with a tip resistance of 3–5 MΩ were prepared for the experiments. On the day of the assay, a dish of cells was removed from the incubator, washed twice with extracellular solution at room temperature, and applied to automated patch-clamp sites. After achieving a whole-cell configuration, the membrane potential was held at −80 mV. A 50 ms pulse to −40 mV was applied to measure leak current, which was subtracted from the tail current in real time. The cells were then depolarized to +20 mV for 2 s, followed by a 1-s pulse to −40 mV to elicit the hERG tail current. This sequence was repeated every 5 s to track the current amplitude. The assay was performed at room temperature, with extracellular solution applied first to stabilize the cell for 5 min. Test compounds were then applied sequentially from lower to higher concentrations on the same cell. Peak tail currents were recorded and plotted against sweep number. The control current amplitude was calculated by averaging five peak tail current measurements at steady state before compound application. The remaining current amplitude after compound inhibition was determined by averaging four to five peak tail current measurements at steady state after compound application.

Percent inhibition was calculated according to the following equation:

% inhibition = [1-(hERG tail amplitude after compound application)/(hERG tail amplitude before compound application)] × 100%

#### Statistical Analysis

Significance was assessed using one-way ANOVA followed by the posthoc Dunnett test, performed with GraphPad Prism software (GraphPad Software, San Diego, CA.

#### Molecular Modeling Study

NAT and NTZ (Compound **6**) were drawn using SybylX2.1, assigned Gasteiger-Huckel charges and energy minimized with the Tripos Force Field. The X-ray crystal structure of the antagonist bound MOR (PDB ID: 4DKL)^31^ was obtained from the Protein Data Bank (PDB) and prepared for docking by adding hydrogen atoms, removing water molecules, and deleting the ligand bound within the binding pocket. HOH718 and HOH719 in the crystal structure were retained since these water molecules are known to be involved in water mediated hydrogen bonding with the 3-OH of the epoxymorphinan skeleton. GOLD 2020,^64^ a genetic algorithm docking program was used to dock the ligands and The binding site was defined to encompass all atoms within 10 Å of the γ-carbon atom of D147, with an added distance constraint between the 17-N atom of the ligands’ epoxymorphinan framework and the carboxylate group of D147. The molecules were docked into the protein with a total of 100 iterations. To optimize the structural models for the ligand-protein complexes, docking was followed by energy minimization under Tripos Force Field in SybylX2.1. CHEMPLP score, which has been optimized for modeling steric complementarity between ligand and protein along with distance and angle dependent hydrogen bonding, was used to obtain plausible docking poses. Figures were generated using PyMOL Molecular Graphics System, version 3.0.4 while the 2D images were obtained using LigPlot+.^72^

## Supporting Information Available

Molecular formula strings (CSV)
NAT_MOR inactive complex (PDB)
NTZ_MOR inactive complex (PDB)
Spectral data of target compounds (^1^H NMR, ^13^C NMR, HRMS); HPLC chromatograms of final compounds; calcium flux assay of compounds 6, 9, and 14 in Gα_qi5_-transfected mMOR-CHO cells (Figure S1); dose–response study of compounds 6, 9, 10, and 14 to antagonize morphine-mediated antinociception (Figure S2); clustering of the 50 docking solutions of NAT and NTZ in the inactive conformation of MOR (Figure S3); *in vitro* metabolic stability study result of compound 6 (NTZ) in human liver microsomes (Table S1); *in vitro* metabolic stability study result of compound 6 (NTZ) in Sprague–Dawley rat liver microsomes (Table S2); cytochrome P450 inhibition study results of compound 6 (NTZ) (Table S3); hERG toxicity study result of compound 6 (NTZ) (Table S4); and *in silico* physicochemical properties prediction of target compounds and NAT and calculation of CNS MPO scores (Table S5) (PDF)

## Supplementary Material

pt4c00593_si_001.csv

pt4c00593_si_002.pdb

pt4c00593_si_003.pdb

## Abbreviations

AC: Activity cliff
BBB: Blood-brain barrier
CHO: Chinese hamster ovary
CNS: Central nervous system
DAMGO: [d-Ala2-MePhe4-Gly(ol)5]enkephalin
DOR: δ opioid receptor
EDCI: 1-ethyl-3-(3-dimethylaminopropyl)carbodiimide
GI: Gastrointestinal
HBD: Hydrogen bond donor
hERG: Human ether-a-go-go related gene
HOBt: Hydroxybenzotriazole
HPLC: High-performance liquid chromatography
KOR: κ opioid receptor
MOR: μ opioid receptor
mp: Melting points
MPE: Maximal possible effect
MPO: Multiparameter optimization
MW: Molecular weight
NIDA: National Institute of Drug Abuse
NLX: Naloxone
NMR: Nuclear magnetic resonance
PNS: Peripheral nervous system
SAR: Structure–activity relationship
s.c.: Subcutaneously
SEM: Standard error of mean
TEA: Triethylamine
TLC: Thin-layer chromatography
TPSA: Topological polar surface area
