# Supplementary material for: Discovery of 6α-Thiazolylcarboxamidonaltrexamine Derivative (NTZ) as a Potent and Central Nervous System Penetrant Opioid Receptor Modulator with Drug-like Properties for Potential Treatment of Opioid Use Disorder
Source: ACS Pharmacol Transl Sci. 2024 Dec 5;7(12):4165–82. doi: 10.1021/acsptsci.4c00593 (PMC11651181; doi:10.1021/acsptsci.4c00593)
Supplement: Supplementary file 4 — pt4c00593_si_004.pdf [file pt4c00593_si_004.pdf]

Supporting Information

Discovery of 6α-Thiazolylcarboxamidonaltrexamine Derivative (NTZ) as a Potent and Central Nervous System Penetrant Opioid Receptor Modulator with Drug-like Properties for Potential Treatment of Opioid Use Disorder

Boshi Huang,[†%] Hongguang Ma,[†%] Piyusha P. Pagare,[†] Mengchu Li,[†] Rolando E. Mendez,[‡] James C. Gillespie,[‡] Justin L. Poklis,[‡] Matthew S. Halquist, [#] David L. Stevens,[‡] William L. Dewey,[‡] Dana E. Selley,[‡] Yan Zhang[†,‡,&*]

[†] *Department of Medicinal Chemistry, School of Pharmacy, Virginia Commonwealth University, 800 E Leigh Street, Richmond, VA 23298, United States*

[‡] *Department of Pharmacology and Toxicology, Virginia Commonwealth University, 410 North 12th Street, Richmond, Virginia 23298, United States*

[#] *Department of Pharmaceutics, Virginia Commonwealth University, 410 North 12th Street, Richmond, Virginia 23298, United States*

[&] *Institute for Drug and Alcohol Studies, 203 East Cary Street, Richmond, Virginia 23298-0059*

[%] These authors contribute equally.

[*] Corresponding author: Tel: +1 (804) 828-0021. E-mail address: yzhang2@vcu.edu (Y. Zhang).

Table of contents

1. Spectral data of target compounds (^{1}H NMR, ^{13}C NMR, HRMS). (*S3 to S26*)
2. HPLC chromatograms of final compounds. (*S27 to S35*)
3. **Figure S1.** Calcium flux assay of compounds **6**, **9** and **14** in Gα_{qi5}-transfected mMOR-CHO cells. (*S36*)
4. **Figure S2.** Dose-response study of compounds **6**, **9**, **10**, and **14** to antagonize morphine mediated antinociception. (*S37*)
5. **Figure S3.** Clustering of the 50 docking solutions of NAT **(A)** and NTZ **(B)** in the inactive conformation of MOR. (*S38*)
6. **Table S1.** In vitro metabolic stability study result of compound **6** (NTZ) in human liver microsomes. (*S39*)
7. **Table S2.** In vitro metabolic stability study result of compound **6** (NTZ) in Sprague-Dawley rat liver microsomes. (*S39*)
8. **Table S3.** Cytochrome P450 inhibition study results of compound **6** (NTZ) (*S40*)
9. **Table S4.** hERG toxicity study result of compound **6** (NTZ) (*S40*)
10. **Table S5.** In-silico physicochemical properties prediction of target compounds and NAT and calculation of CNS MPO scores. (*S41*)

1. Spectral data for target compounds (^{1}H NMR, ^{13}C NMR, HRMS, and HPLC graphs)

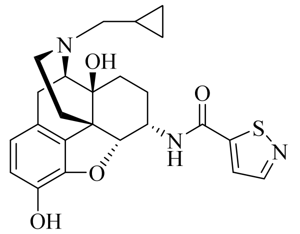

1

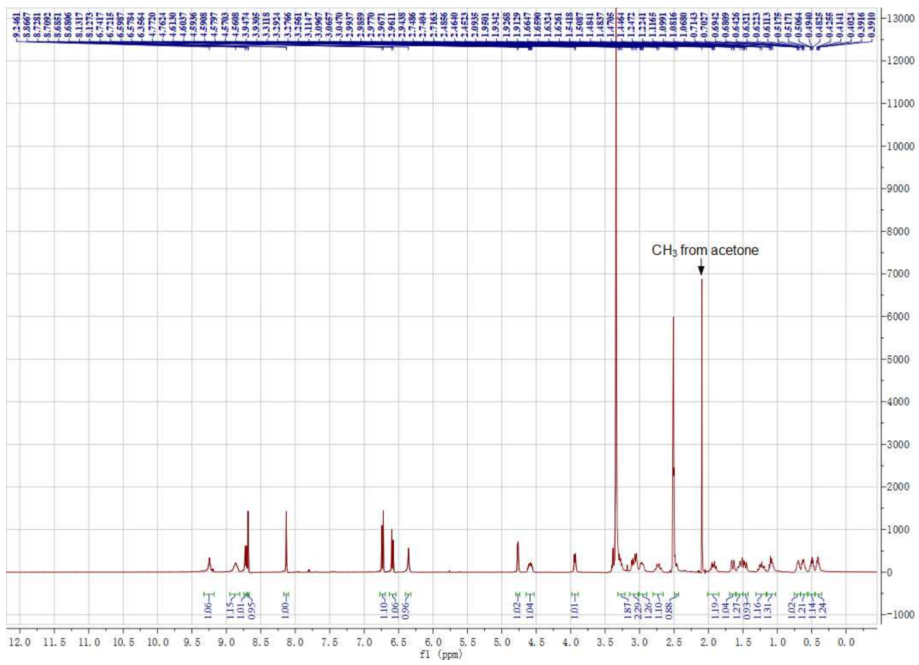

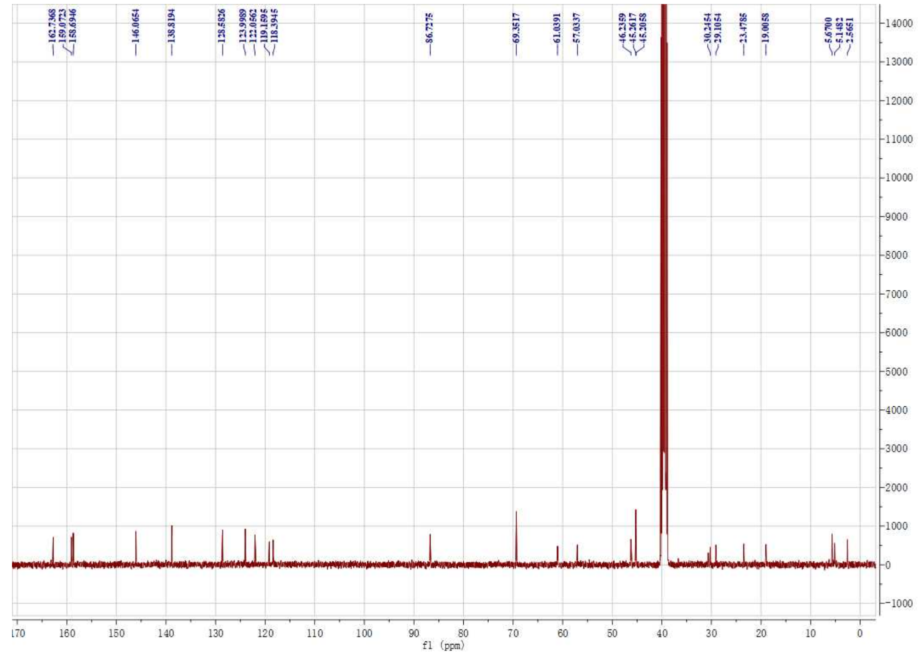

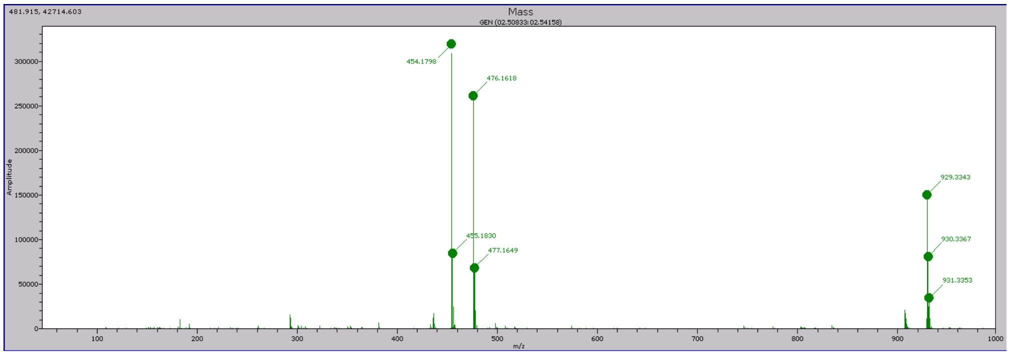
Mass
454.1798
476.1618
455.1830
477.1649
929.3343
930.3367
931.3353
Amplitude
m/z

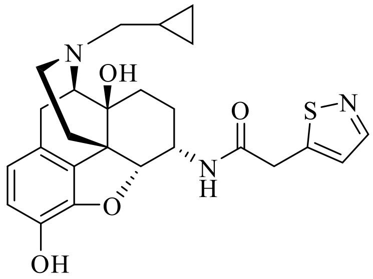
N
OH
O
S
N
N
H
O
OH

2

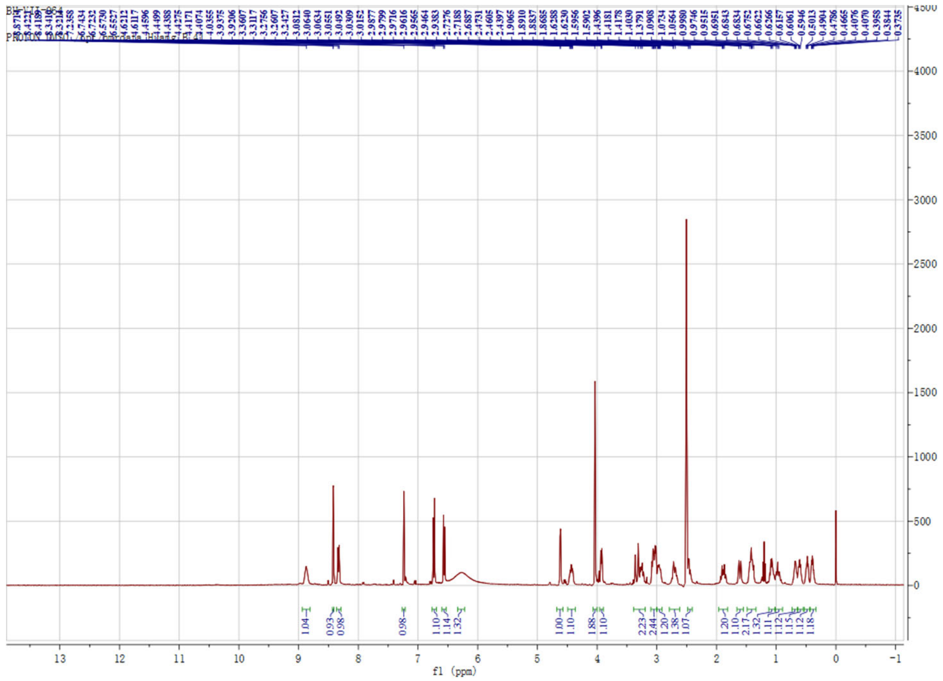
f1 (ppm)

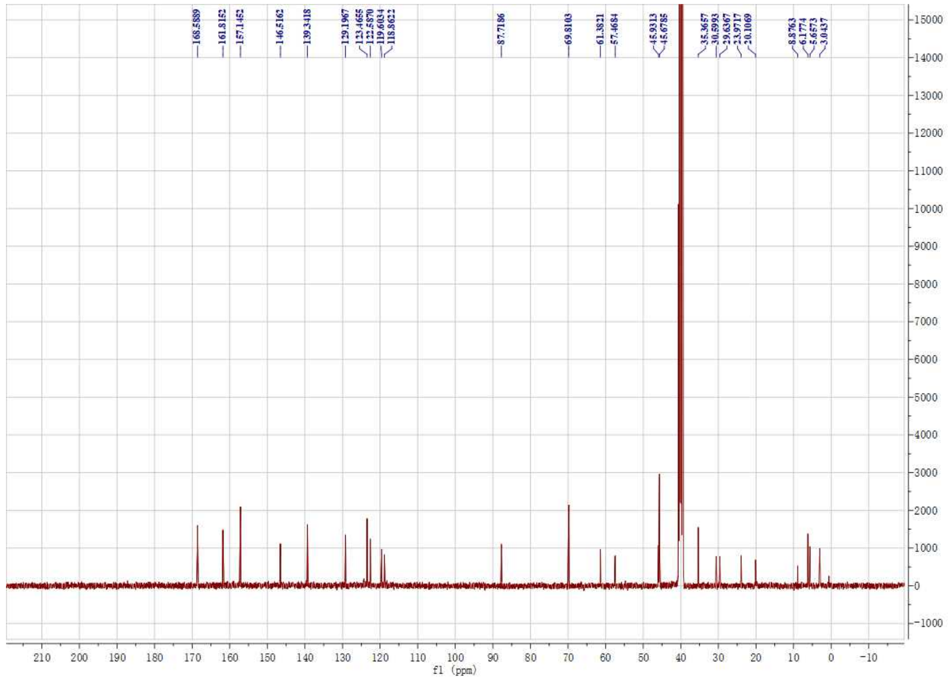
168.5889
161.8152
157.1452
146.5162
139.3418
129.1967
123.4655
122.5870
119.6034
118.8622
87.7186
69.8103
61.3821
57.4684
45.9313
45.6785
35.3657
30.5993
29.6367
23.9717
20.1069
8.8763
6.1774
5.6573
3.0437
f1 (ppm)

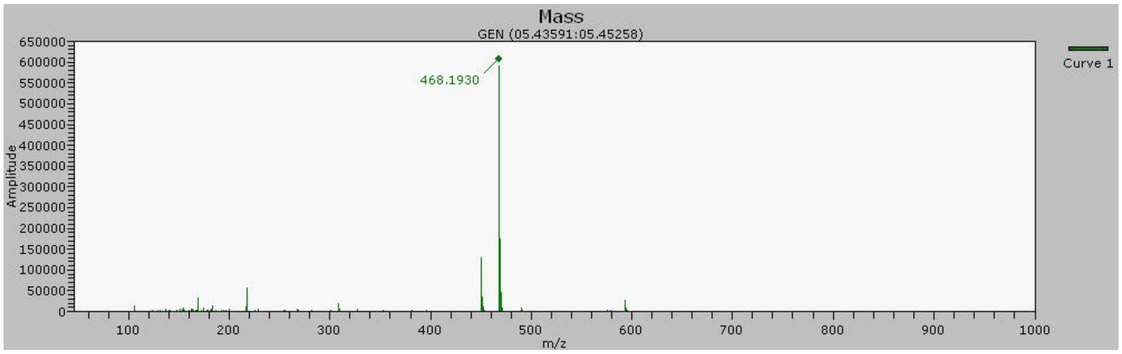
Mass
GEN (05.43591:05.45258)
468.1930
Amplitude
m/z
Curve 1

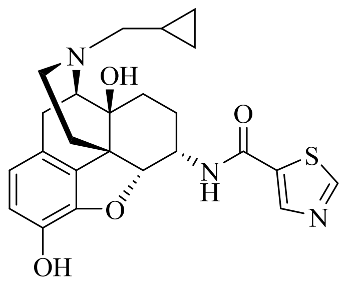

3

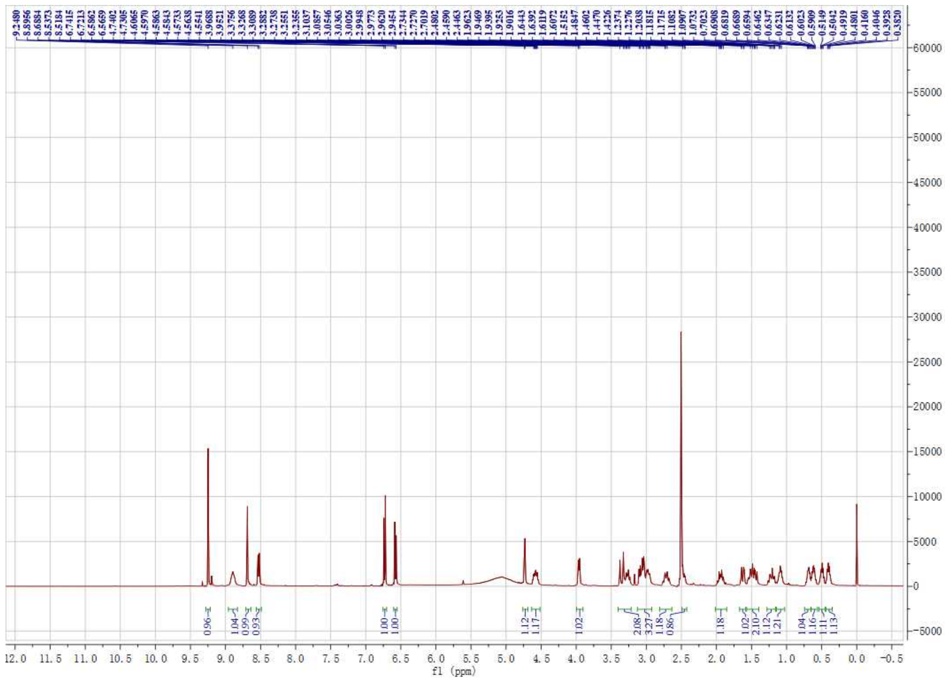

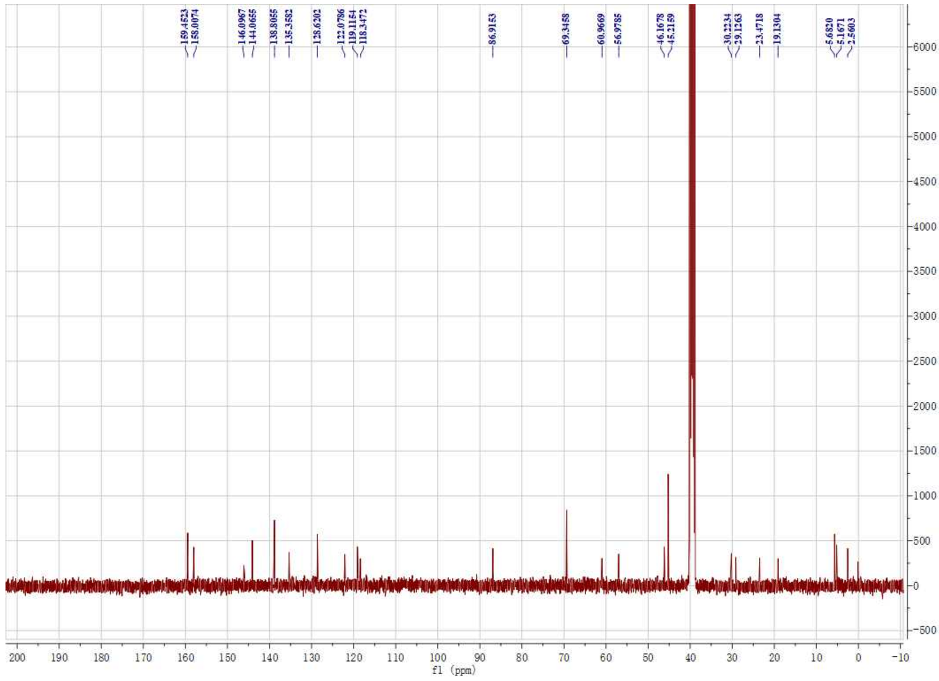

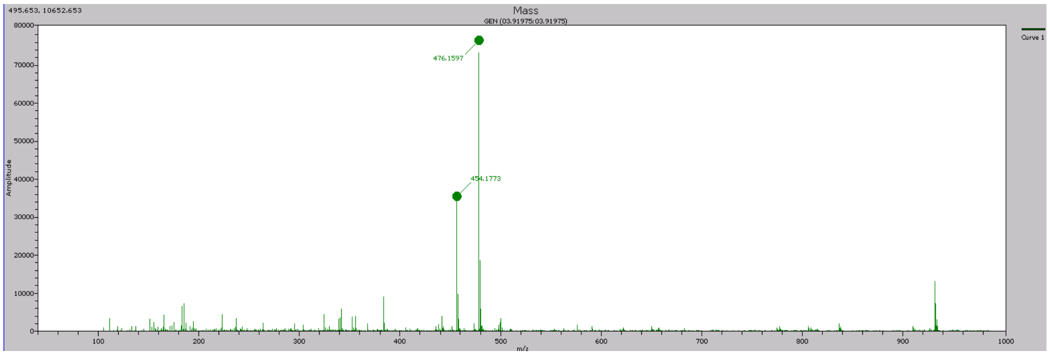
Mass
476.1597
454.1773
Amplitude
m/z

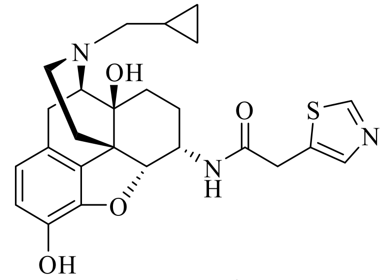
N
OH
O
S
N
N
H
O
OH

4

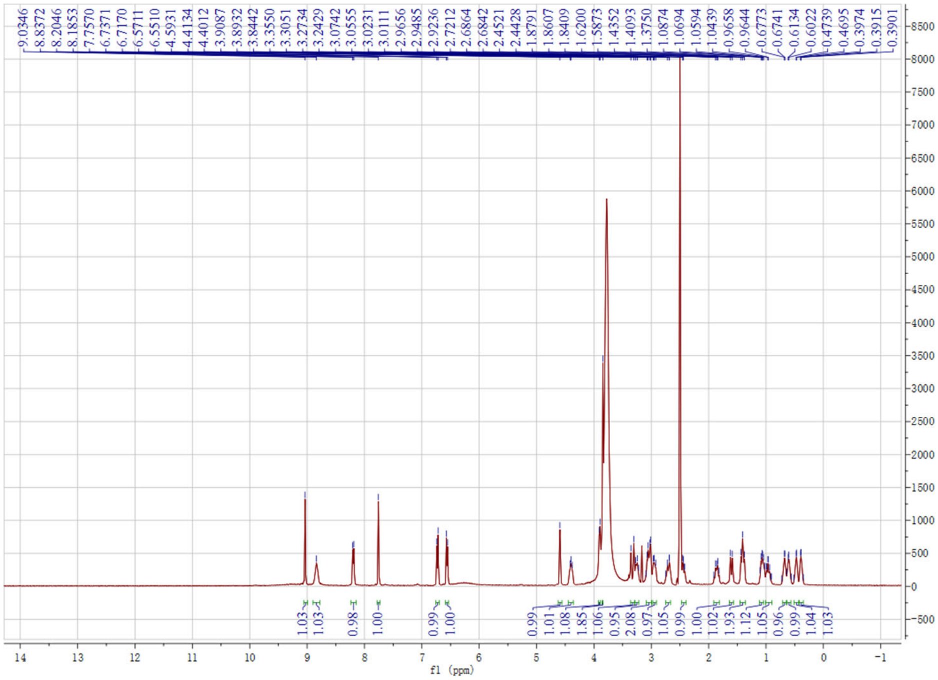
9.0346
8.8372
8.2046
8.1853
7.7570
6.7371
6.7170
6.5711
6.5510
4.5931
4.4134
4.4012
3.9087
3.8932
3.8442
3.3550
3.3051
3.2734
3.2429
3.0742
3.0555
3.0231
3.0111
2.9656
2.9485
2.9236
2.7212
2.6864
2.6842
2.4521
2.4428
1.8791
1.8607
1.8409
1.6200
1.5873
1.4352
1.4093
1.3750
1.0874
1.0694
1.0594
1.0439
0.9658
0.9644
0.6773
0.6741
0.6134
0.6022
0.4739
0.4695
0.3974
0.3915
0.3901
1.03
1.03
0.98
1.00
0.99
1.00
0.99
1.01
1.08
1.85
1.06
0.95
2.08
0.97
1.05
0.99
1.00
1.02
1.93
1.12
1.05
0.96
0.99
1.04
1.03
f1 (ppm)

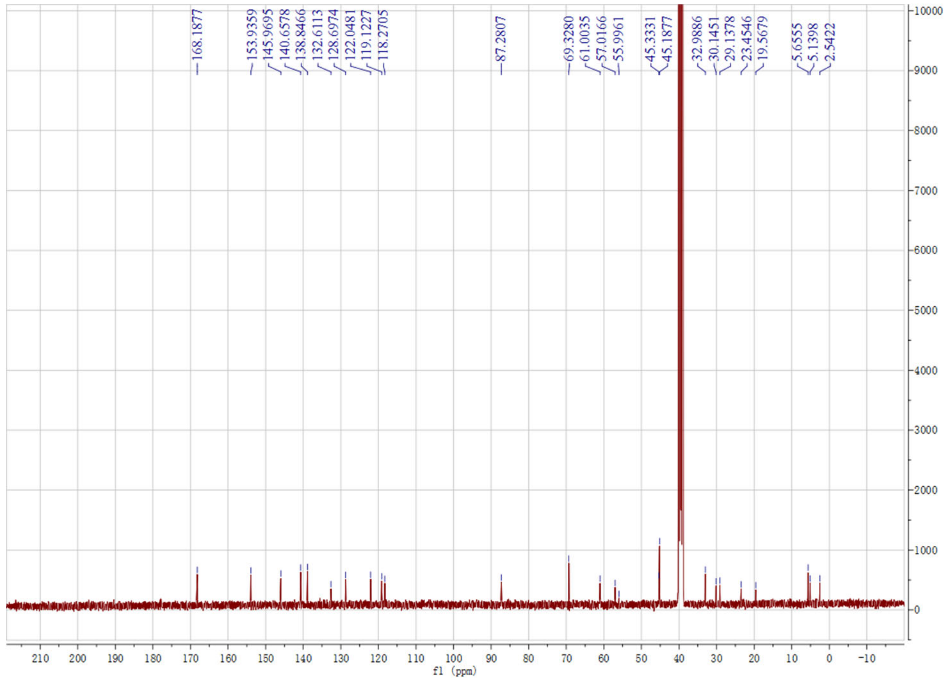
168.1877
153.9359
145.9695
140.6578
138.8466
132.6113
128.6974
122.0481
119.1227
118.2705
87.2807
69.3280
61.0035
57.0166
55.9961
45.3331
45.1877
32.9886
30.1451
29.1378
23.4546
19.5679
5.6555
5.1398
2.5422
f1 (ppm)

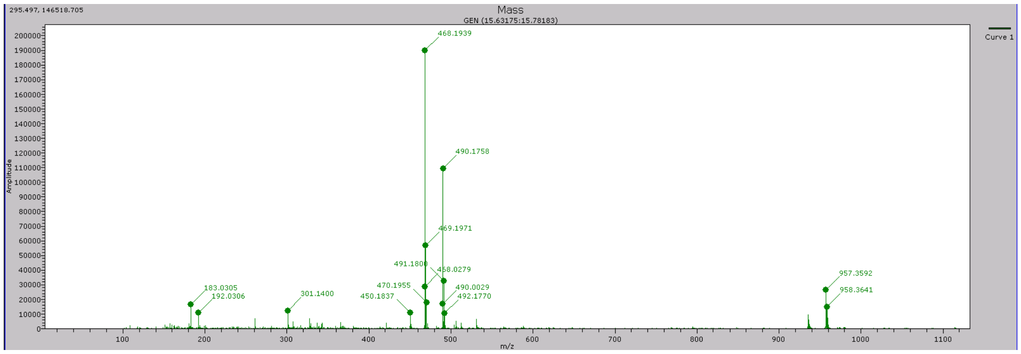
295.497, 146510.705
Mass
GEN (15.63175:15.78183)
Curve 1
Amplitude
m/z
468.1939
490.1758
469.1971
491.1800
468.0279
470.1955
450.1837
490.0029
492.1770
183.0305
192.0306
301.1400
957.3592
958.3641

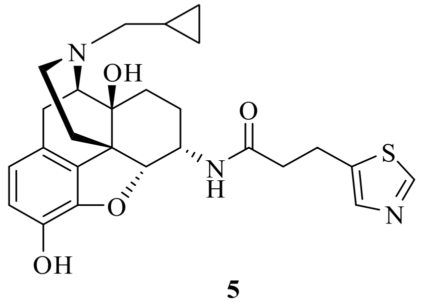

5

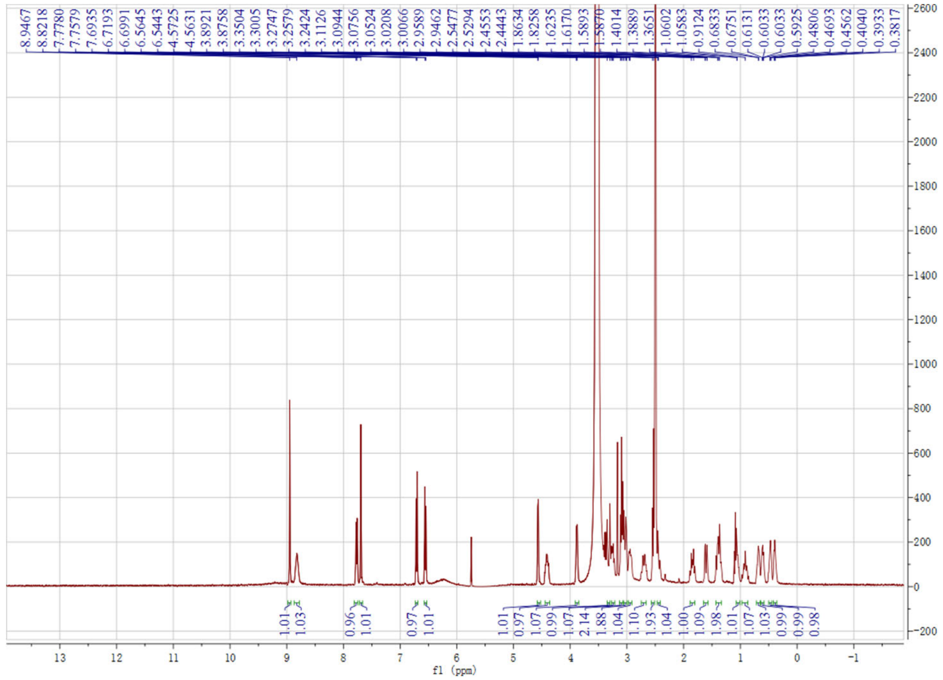

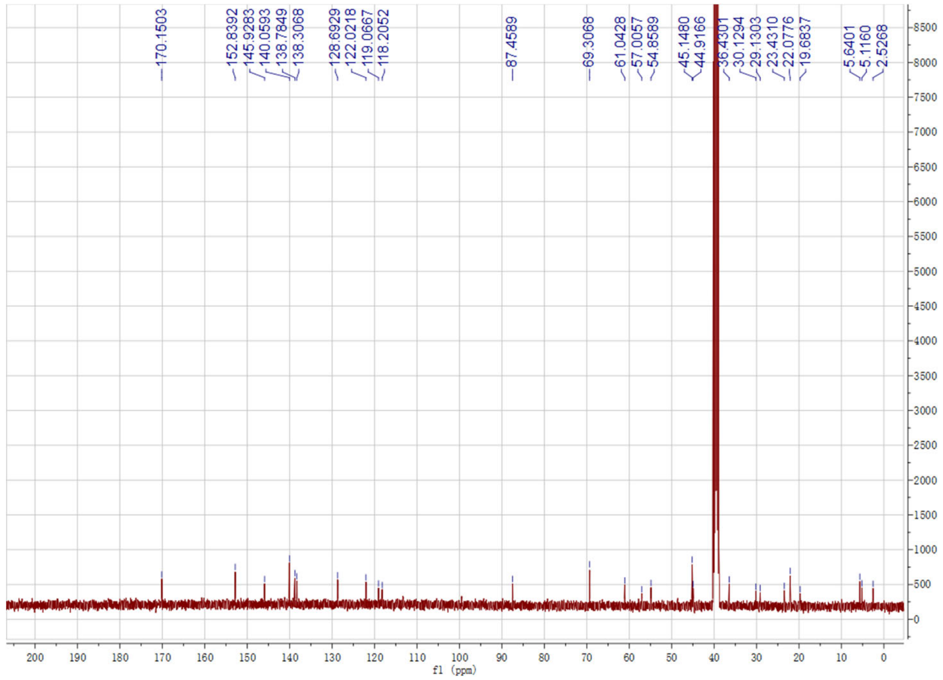

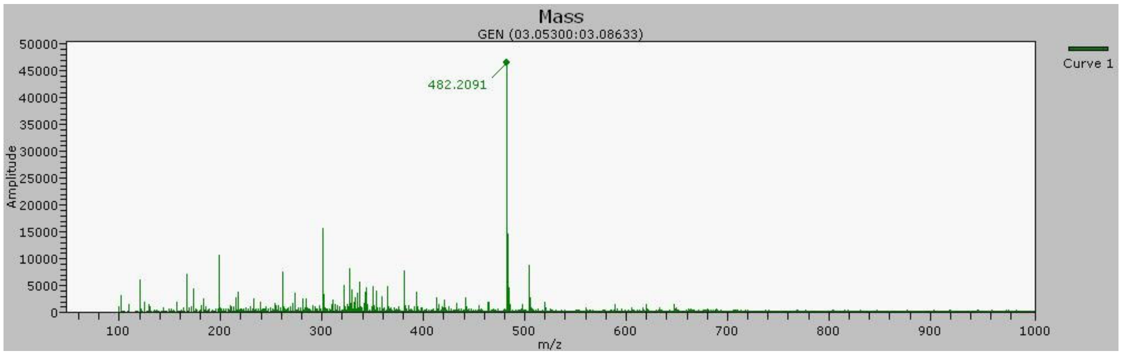
Mass
GEN (03.05300:03.08633)
482.2091
Curve 1
Amplitude
m/z

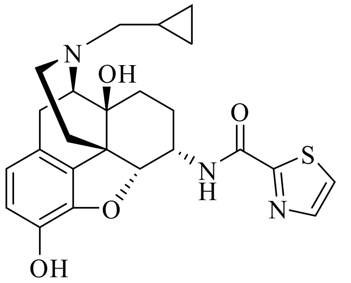

6

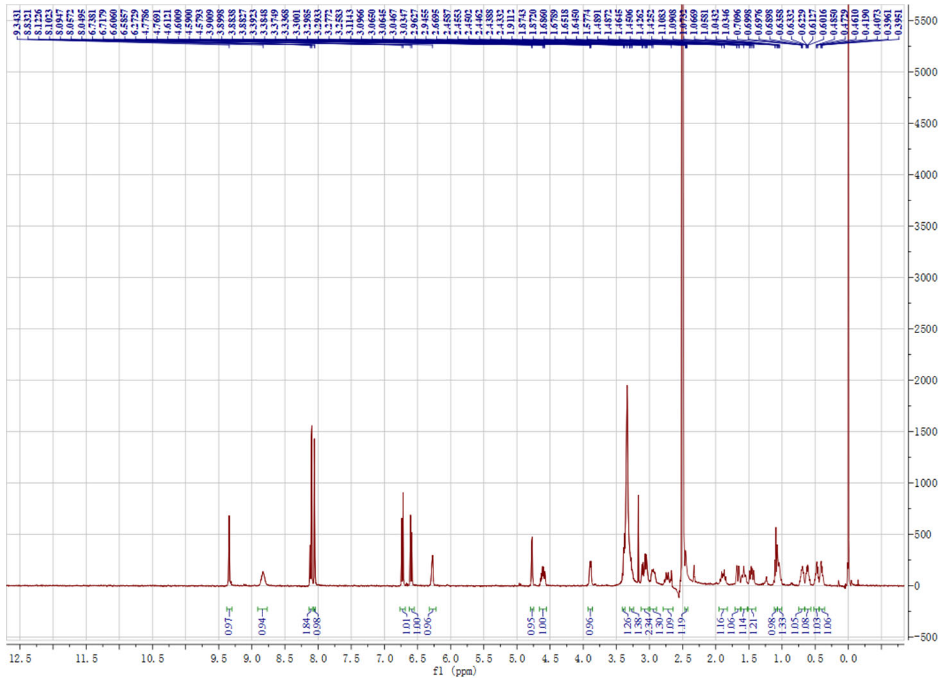
f1 (ppm)

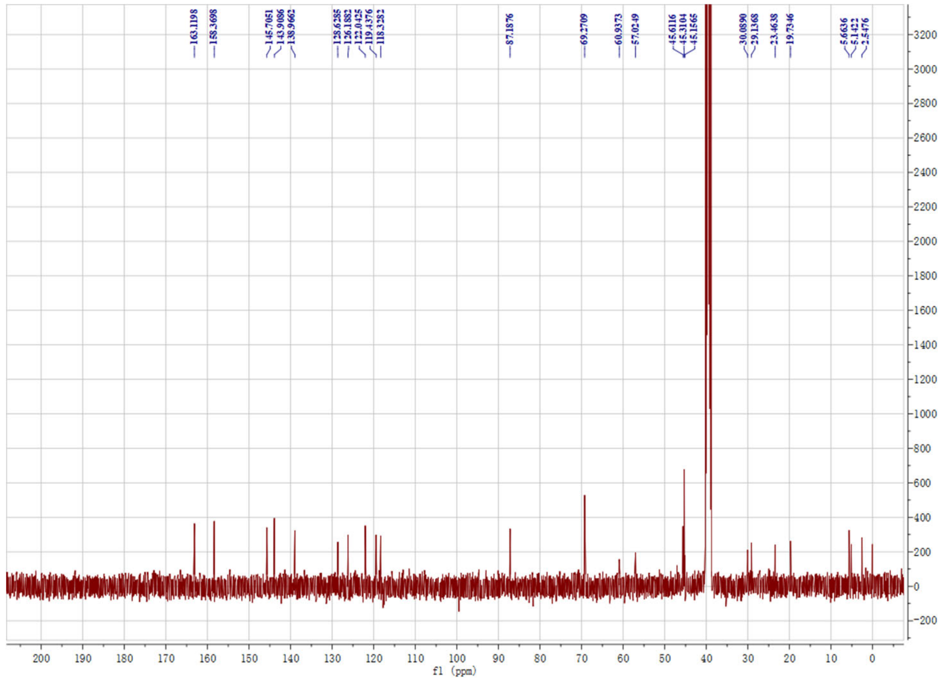
163.1198
158.3698
145.7061
143.9086
138.9662
128.6285
126.1882
122.0425
119.4376
118.3282
87.1876
69.2709
60.9373
57.0249
45.6116
45.3104
45.1565
30.0890
29.1368
23.4638
19.7346
5.6636
5.1422
2.5476
f1 (ppm)

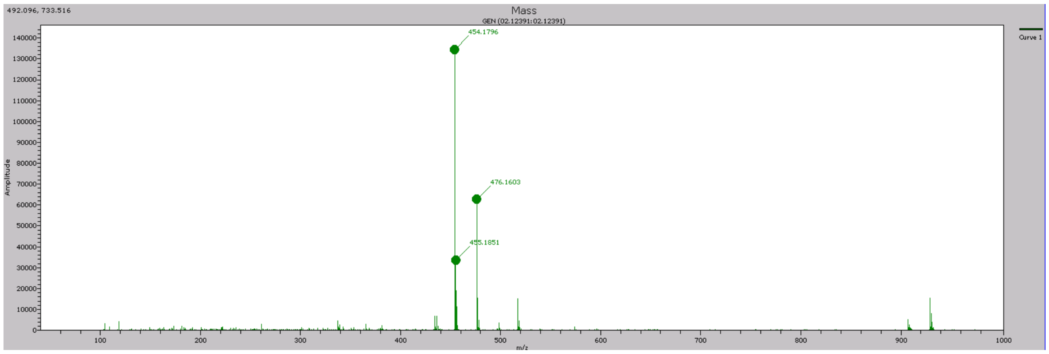
492.096, 733.516
Mass
GEN (02.12391:02.12391)
454.1796
476.1603
455.1851
Amplitude
m/z
Curve 1

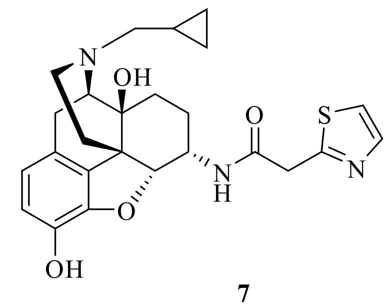

7

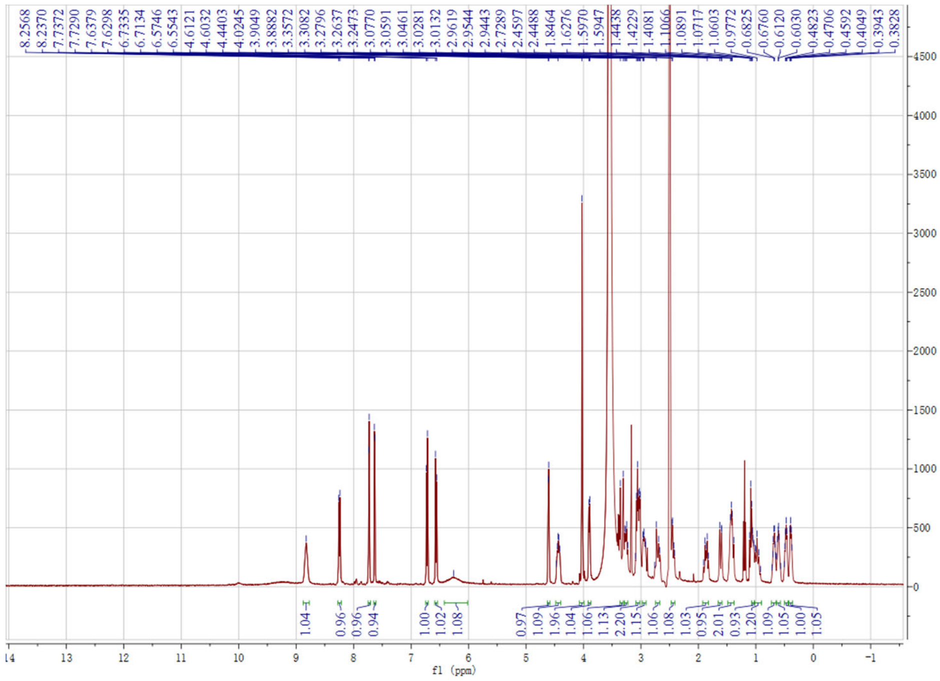

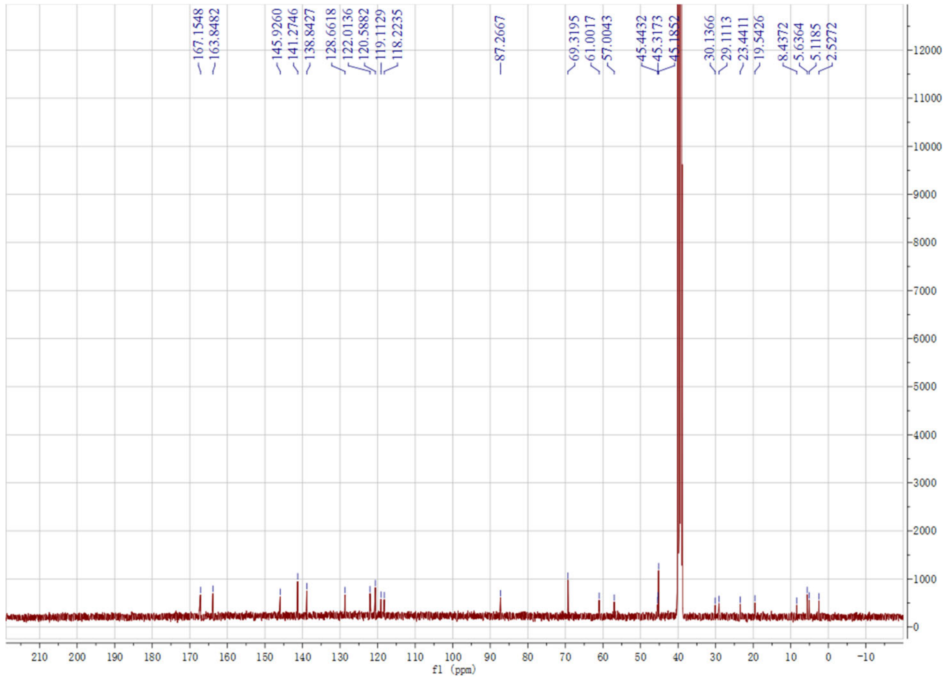

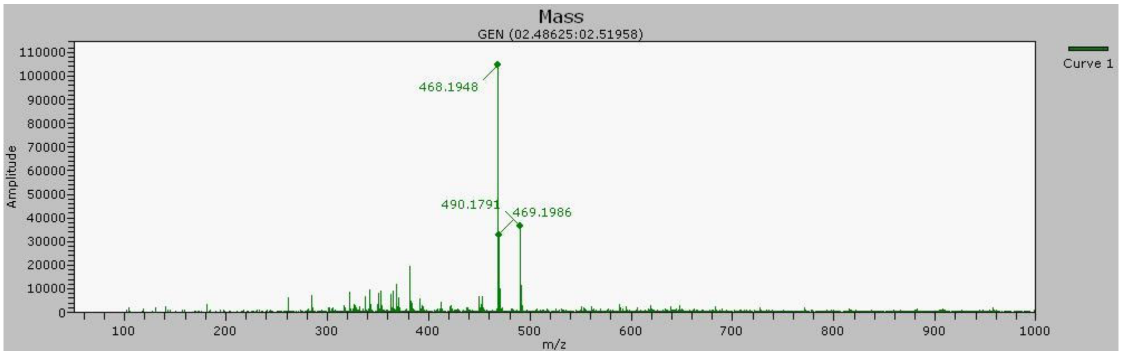

Mass
GEN (02.48625:02.51958)
Curve 1
Amplitude
m/z
468.1948
490.1791
469.1986

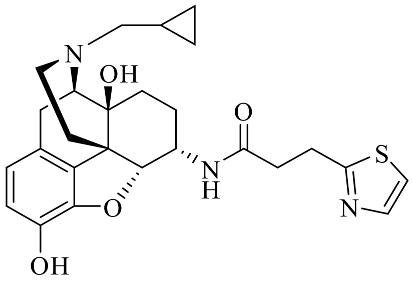

N
OH
O
S
N
H
O
OH

8

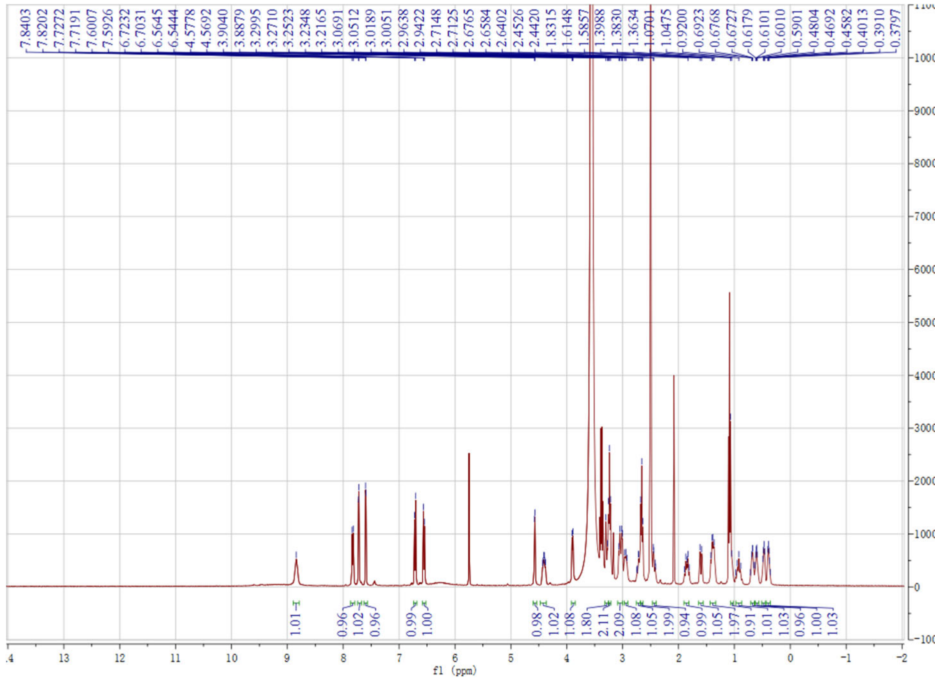

7.8403
7.8202
7.7272
7.7191
7.6007
7.5926
6.7232
6.7031
6.5645
6.5444
4.5778
4.5692
3.9040
3.8879
3.2995
3.2710
3.2523
3.2348
3.2165
3.0691
3.0512
3.0189
3.0051
2.9638
2.9422
2.7148
2.7125
2.6765
2.6584
2.6402
2.4526
2.4420
1.8315
1.6148
1.5857
1.3988
1.3830
1.3634
1.0701
1.0475
0.9200
0.6923
0.6768
0.6727
0.6179
0.6101
0.6010
0.5901
0.4804
0.4692
0.4582
0.4013
0.3910
0.3797
f1 (ppm)

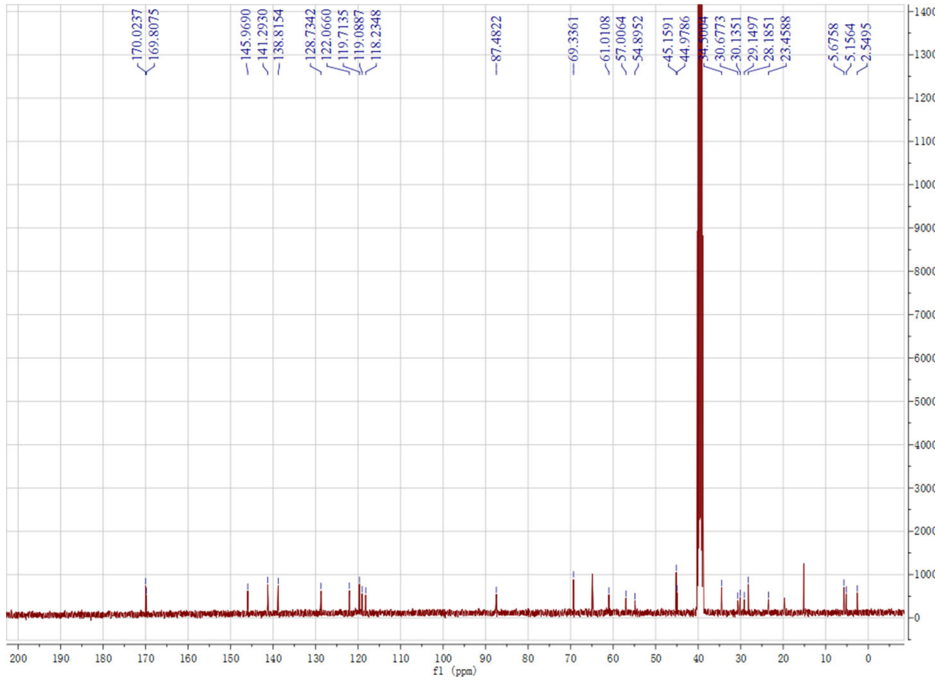
170.0237
169.8075
145.9690
141.2930
138.8154
128.7342
122.0660
119.7135
119.0887
118.2348
87.4822
69.3361
61.0108
57.0064
54.8952
45.1591
44.9786
30.6773
30.1351
29.1497
28.1851
23.4588
5.6758
5.1564
2.5495
f1 (ppm)

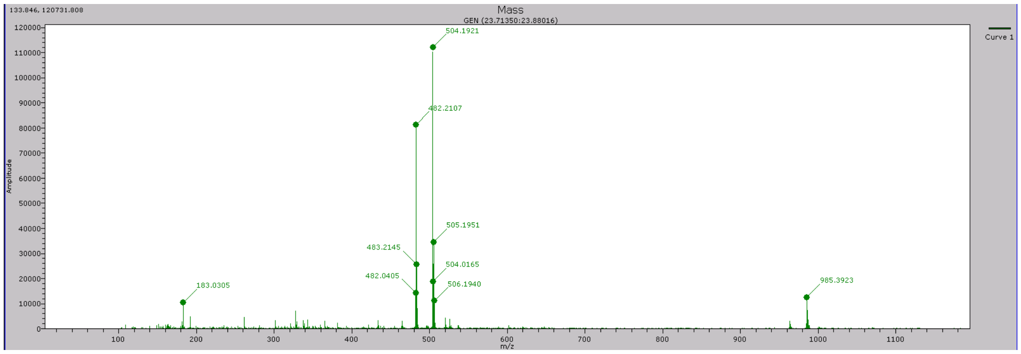
Mass
Amplitude
m/z
Curve 1
504.1921
482.2107
505.1951
483.2145
504.0165
482.0405
506.1940
183.0305
985.3923

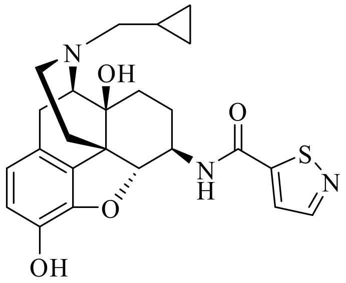

9

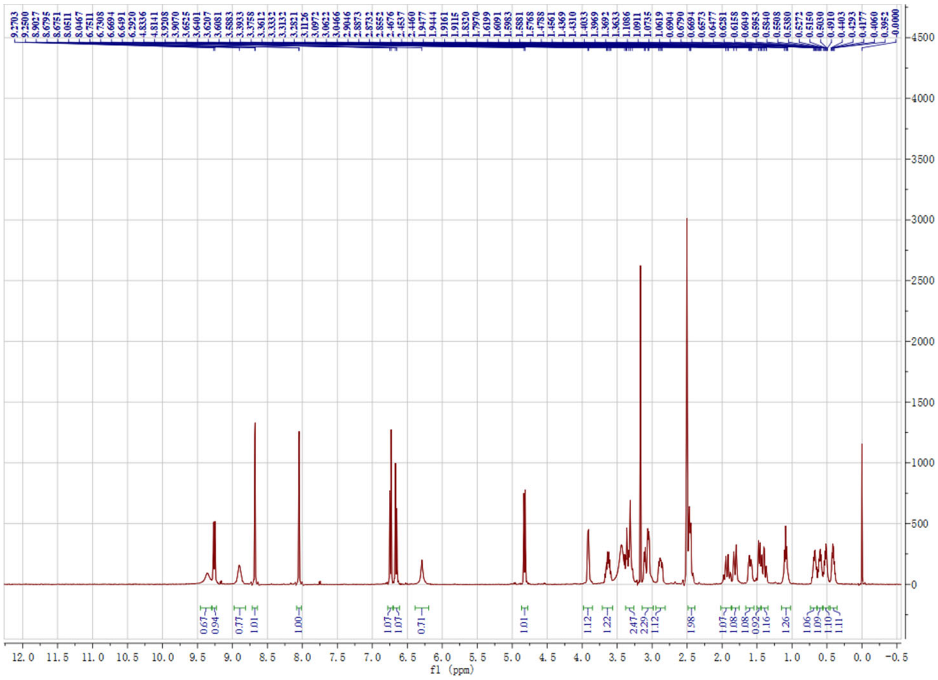

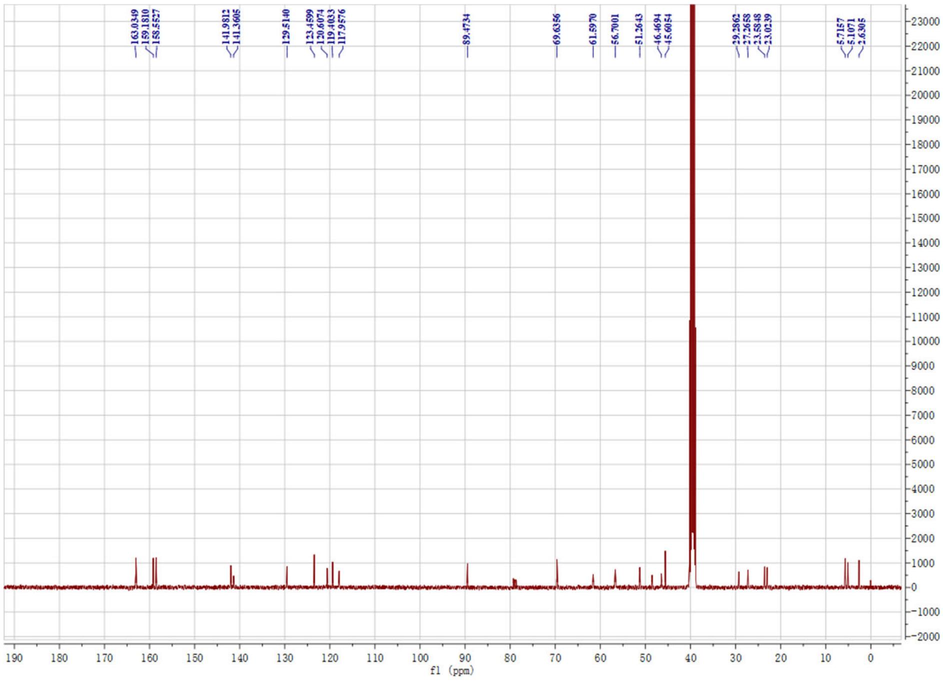

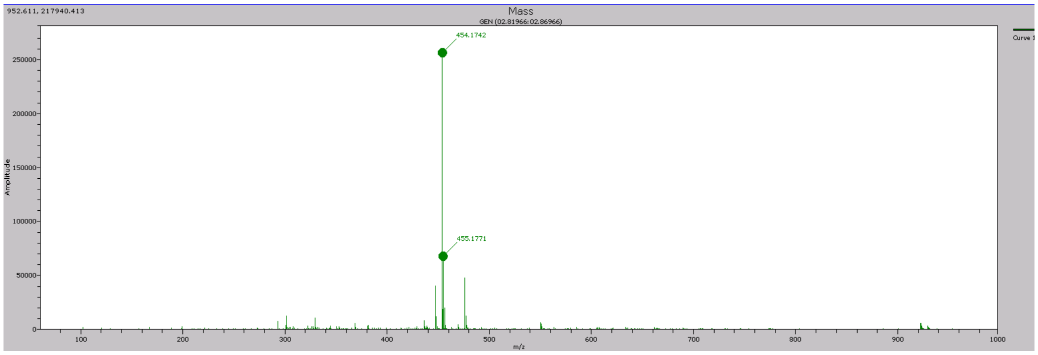
952.611, 217940.413
Mass
GEN (02.81966:02.86966)
454.1742
455.1771
Amplitude
m/z
Curve 1

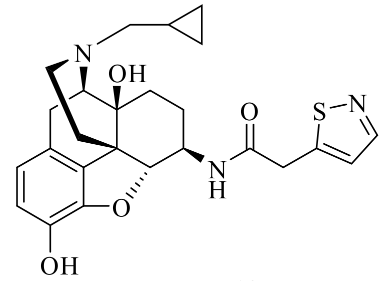
N
OH
O
S
N
N
H
O
OH

10

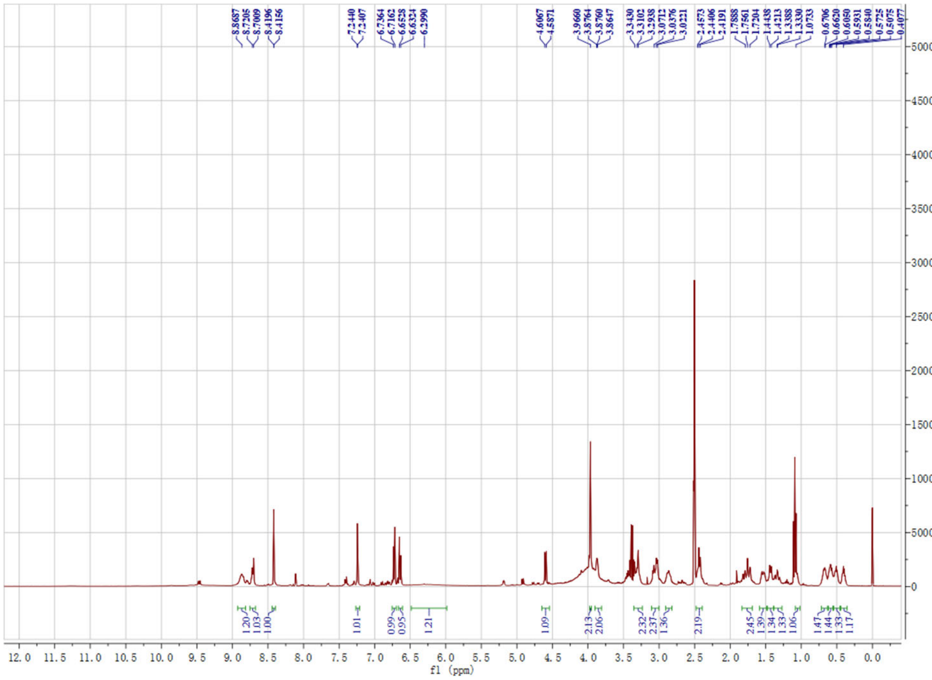
f1 (ppm)

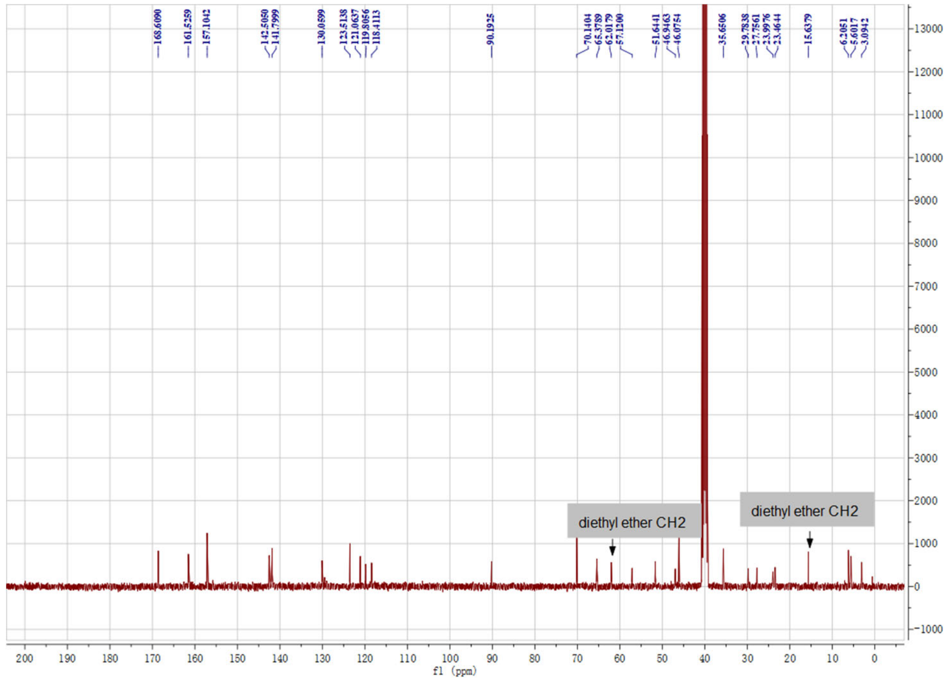
168.6090
161.5259
157.1042
142.5050
141.7999
130.0599
123.5138
121.0637
119.8056
118.4113
90.1925
70.1404
65.3789
62.0179
57.1200
51.6441
46.9463
46.0754
35.6506
29.7838
27.7561
23.9976
23.4644
15.6379
6.2051
5.6017
3.0942
diethyl ether CH2
diethyl ether CH2
13000
12000
11000
10000
9000
8000
7000
6000
5000
4000
3000
2000
1000
0
-1000
200 190 180 170 160 150 140 130 120 110 100 90 80 70 60 50 40 30 20 10 0
f1 (ppm)

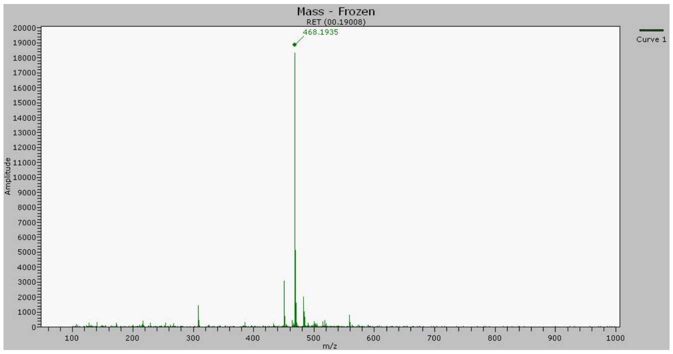
Mass - Frozen
RET (00.19008)
468.1935
Curve 1
Amplitude
20000
19000
18000
17000
16000
15000
14000
13000
12000
11000
10000
9000
8000
7000
6000
5000
4000
3000
2000
1000
0
100 200 300 400 500 600 700 800 900 1000
m/z

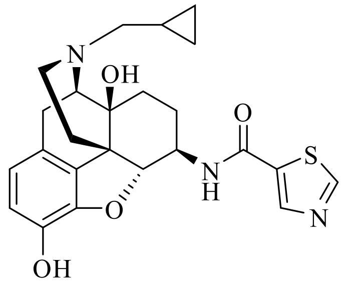

11

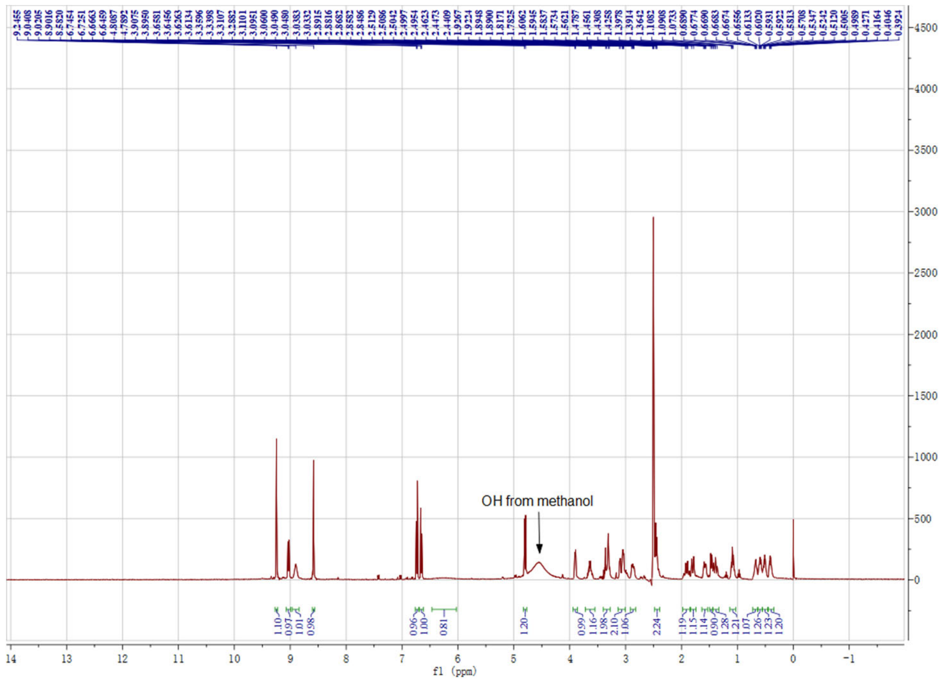

OH from methanol
f1 (ppm)

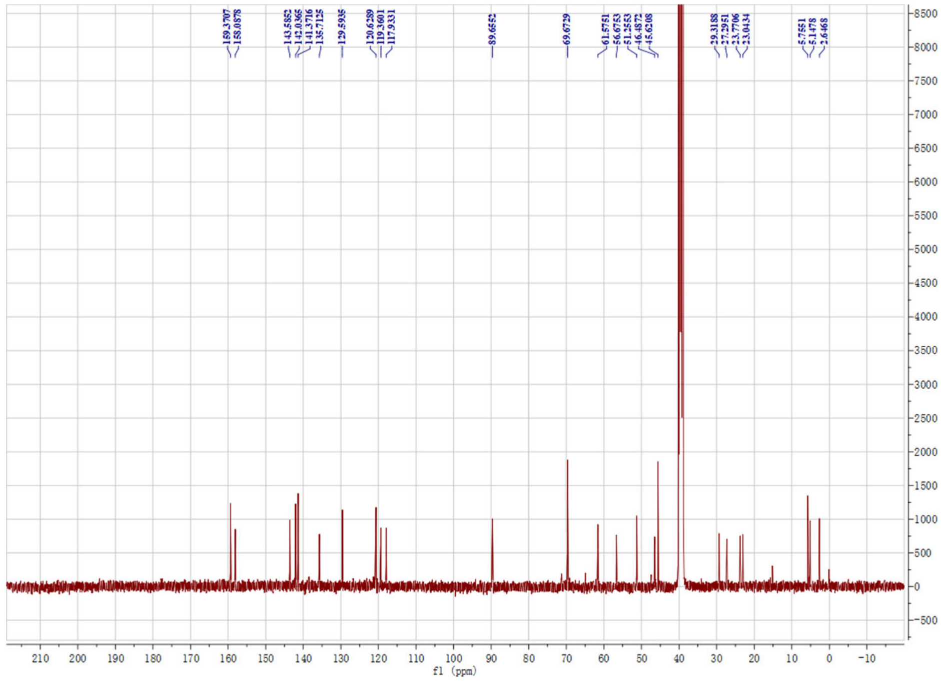

159.3707
158.0878
143.5852
142.0365
141.3716
135.7125
129.5935
120.6289
119.3601
117.9331
89.6552
69.6729
61.5761
56.6753
51.2553
46.4872
45.6208
29.3188
27.2951
23.7706
23.0434
5.7551
5.1478
2.6468
f1 (ppm)

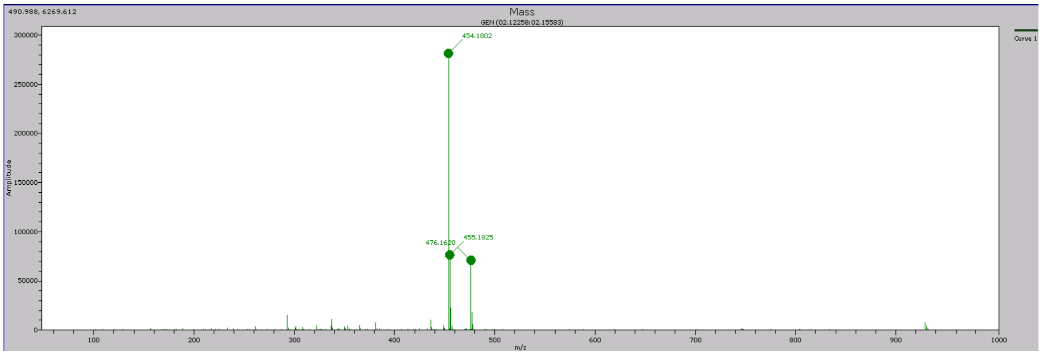
Mass
454.1802
476.1620
455.1825
Amplitude
m/z
Curve 1

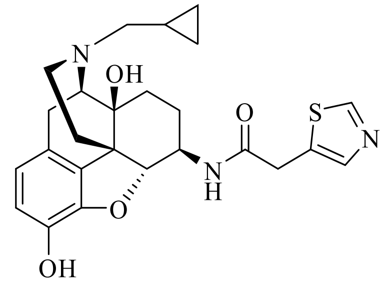
N
OH
O
S
N
N
H
O
OH

12

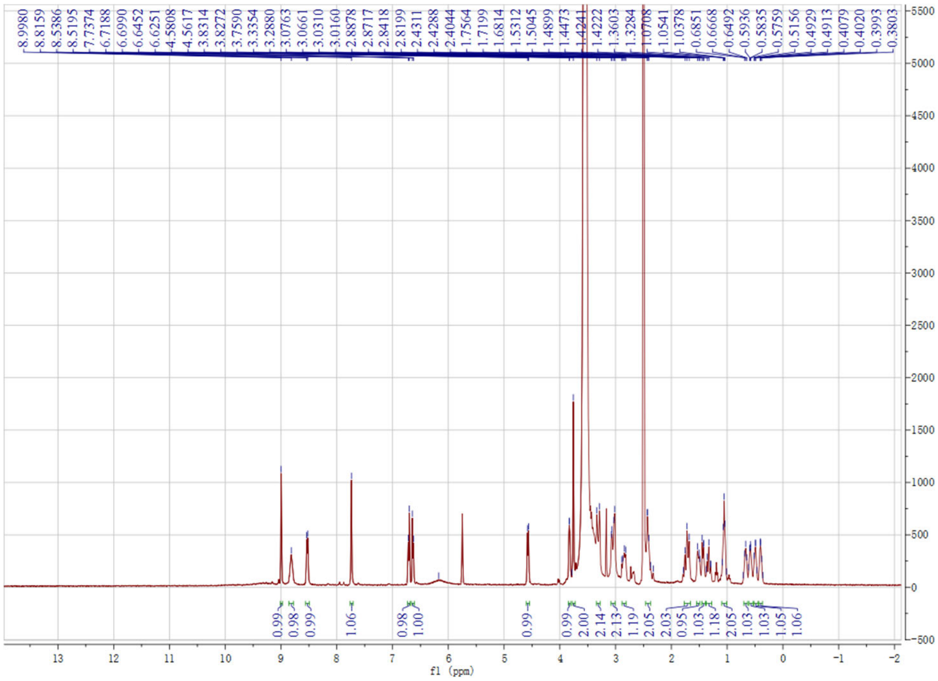
8.9980
8.8159
8.5386
8.5195
7.7374
6.7188
6.6990
6.6452
6.6251
4.5808
4.5617
3.8314
3.8272
3.7590
3.3354
3.2880
3.0763
3.0661
3.0310
3.0160
2.8878
2.8717
2.8418
2.8199
2.4311
2.4288
2.4044
1.7564
1.7199
1.6814
1.5312
1.5045
1.4899
1.4473
1.4241
1.4222
1.3603
1.3284
1.0708
1.0541
1.0378
0.6851
0.6668
0.6492
0.5936
0.5835
0.5759
0.5156
0.4929
0.4913
0.4079
0.4020
0.3993
0.3803
0.99
0.98
0.99
1.06
0.98
1.00
0.99
0.99
2.00
2.14
2.13
1.19
2.05
2.03
0.95
1.03
1.18
2.05
1.03
1.03
1.05
1.06
f1 (ppm)

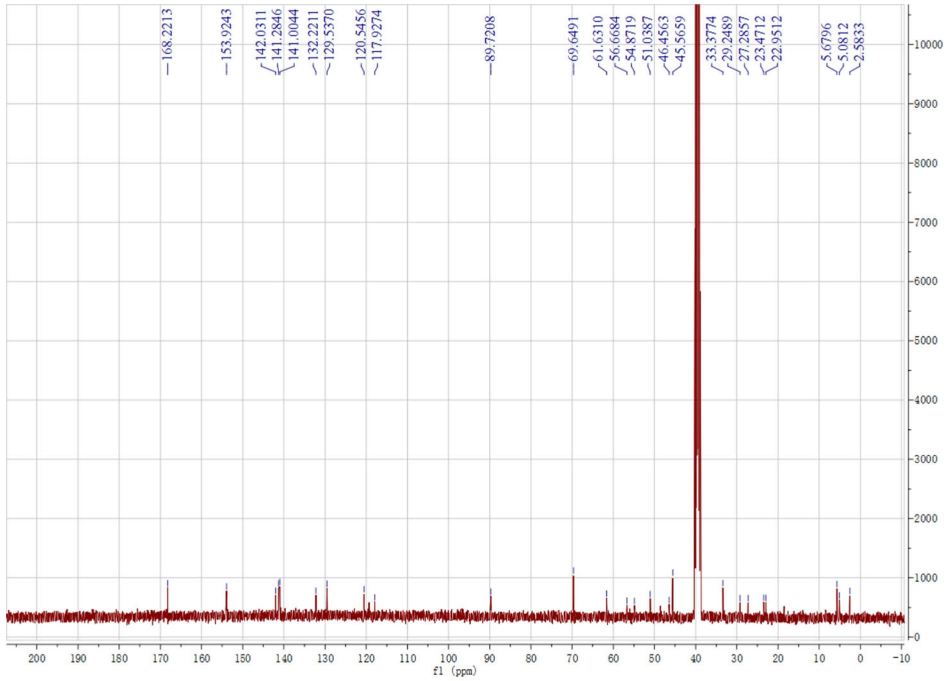

168.2213
153.9243
142.0311
141.2846
141.0044
132.2211
129.5370
120.5456
117.9274
89.7208
69.6491
61.6310
56.6684
54.8719
51.0387
46.4563
45.5659
33.3774
29.2489
27.2857
23.4712
22.9512
5.6796
5.0812
2.5833
f1 (ppm)

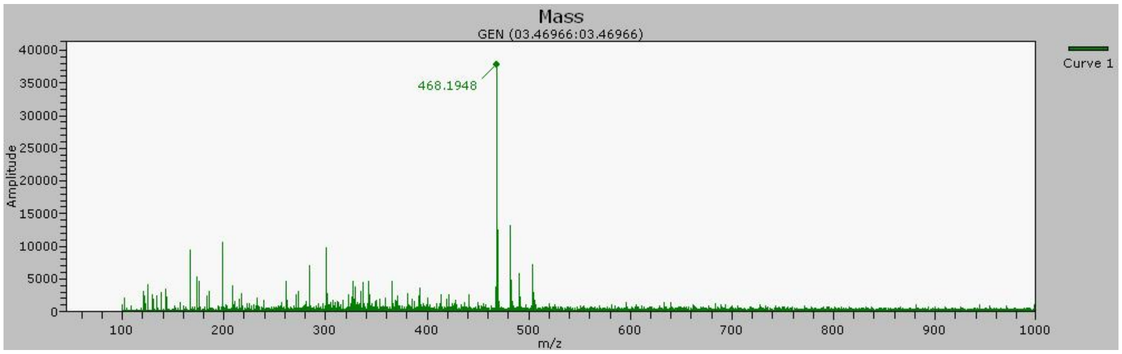

Mass
GEN (03.46966:03.46966)
468.1948
Curve 1
Amplitude
m/z

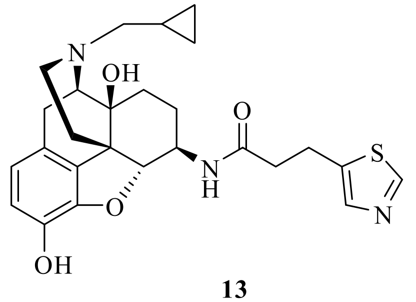
N
OH
O
N
H
S
N
O
OH

13

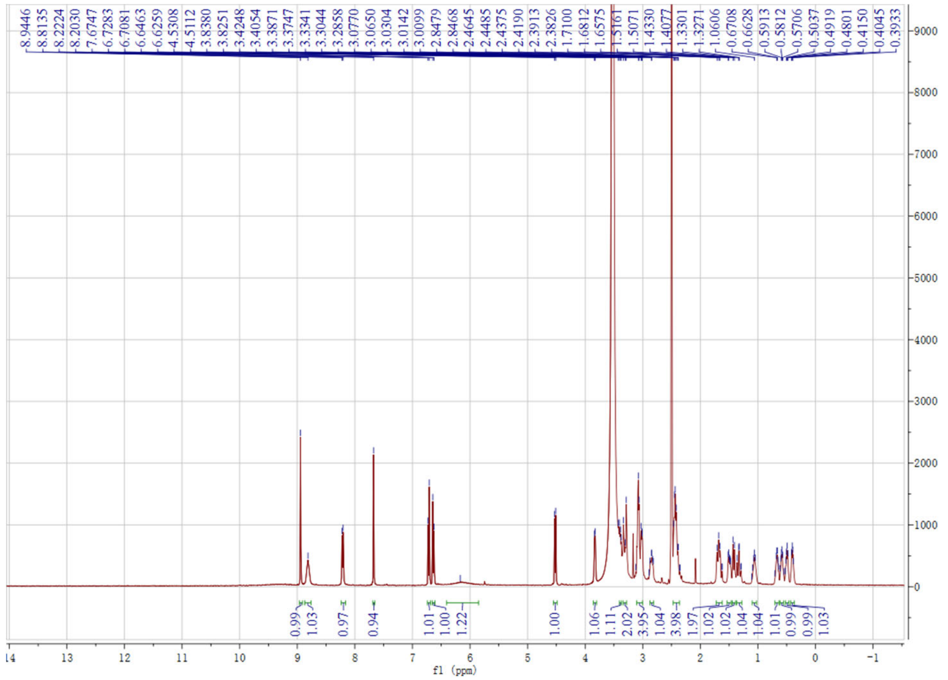
8.9446
8.8135
8.2224
8.2030
7.6747
6.7283
6.7081
6.6463
6.6259
4.5308
4.5112
3.8380
3.8251
3.4248
3.4054
3.3871
3.3747
3.3341
3.3044
3.2858
3.0770
3.0650
3.0304
3.0142
3.0099
2.8479
2.8468
2.4645
2.4485
2.4375
2.4190
2.3913
2.3826
1.7100
1.6812
1.6575
1.5161
1.5071
1.4330
1.4077
1.3301
1.3271
1.0606
0.6708
0.6628
0.5913
0.5812
0.5706
0.5037
0.4919
0.4801
0.4150
0.4045
0.3933
0.99
1.03
0.97
0.94
1.01
1.00
1.22
1.00
1.06
1.11
2.02
3.95
1.04
3.98
1.97
1.02
1.02
1.04
1.04
1.01
0.99
0.99
1.03
f1 (ppm)

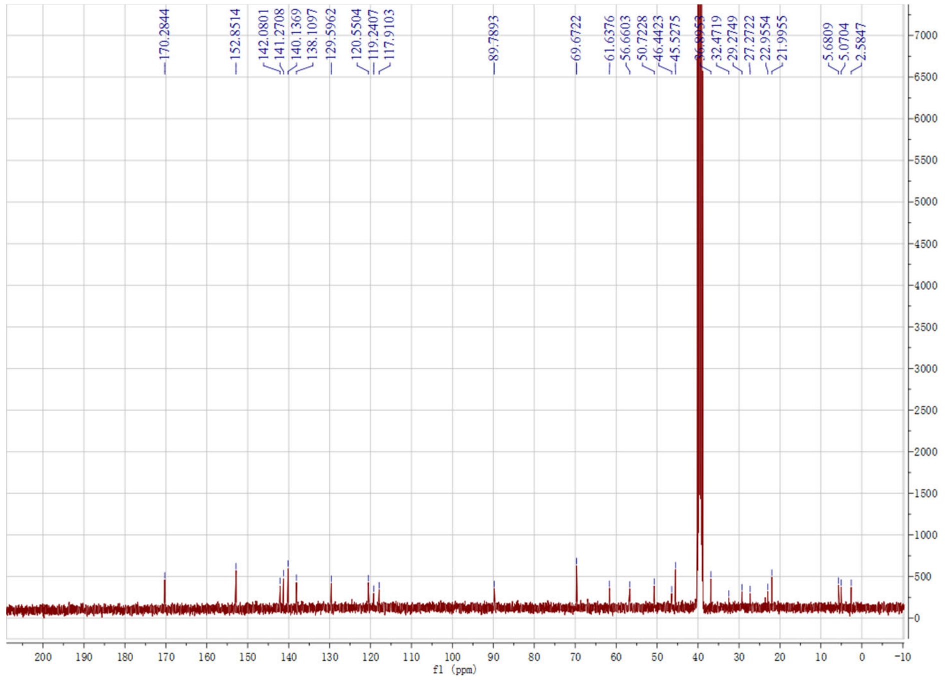
170.2844
152.8514
142.0801
141.2708
140.1369
138.1097
129.5962
120.5504
119.2407
117.9103
89.7893
69.6722
61.6376
56.6603
50.7228
46.4423
45.5275
36.8953
32.4719
29.2749
27.2722
22.9554
21.9955
5.6809
5.0704
2.5847
f1 (ppm)

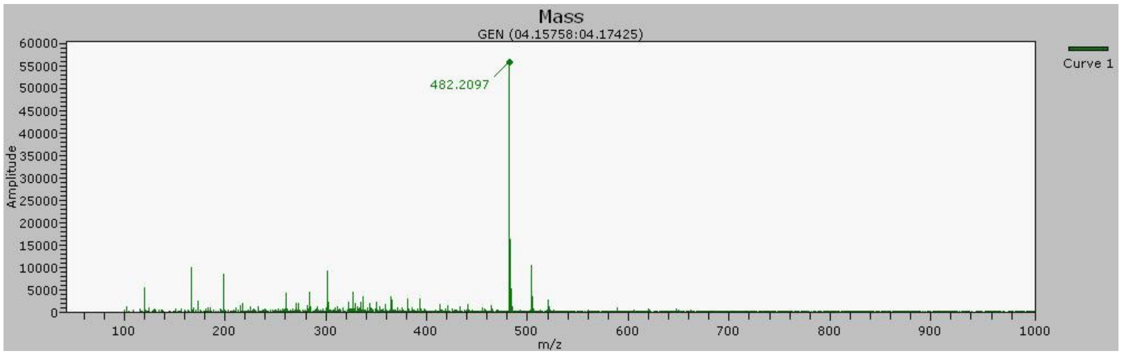
Mass
GEN (04.15758:04.17425)
482.2097
Curve 1
Amplitude
m/z

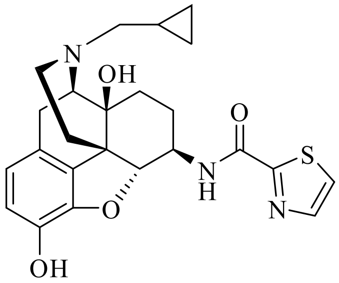

14

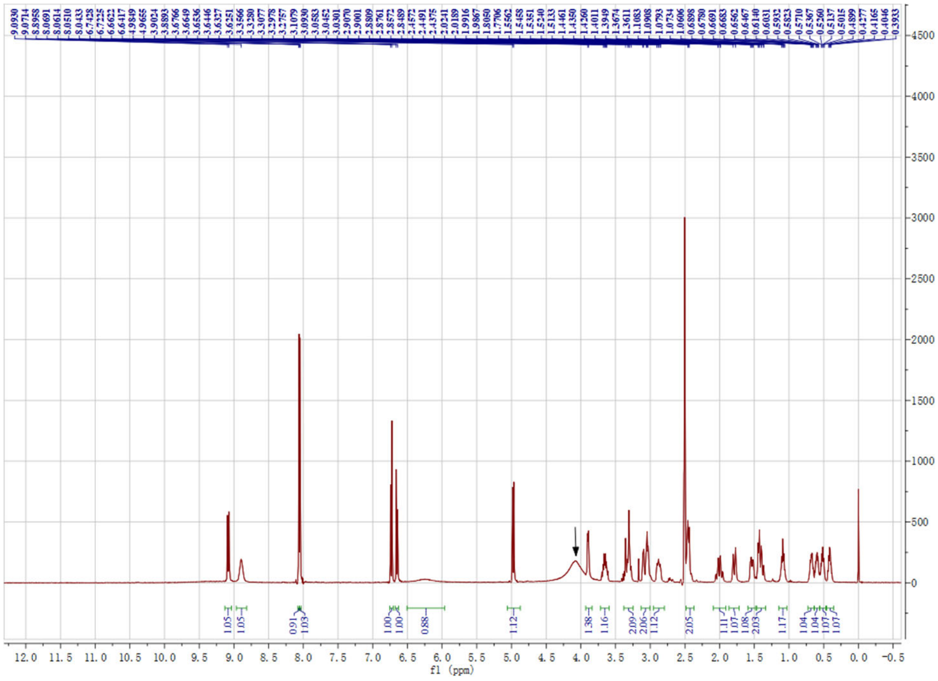
f1 (ppm)

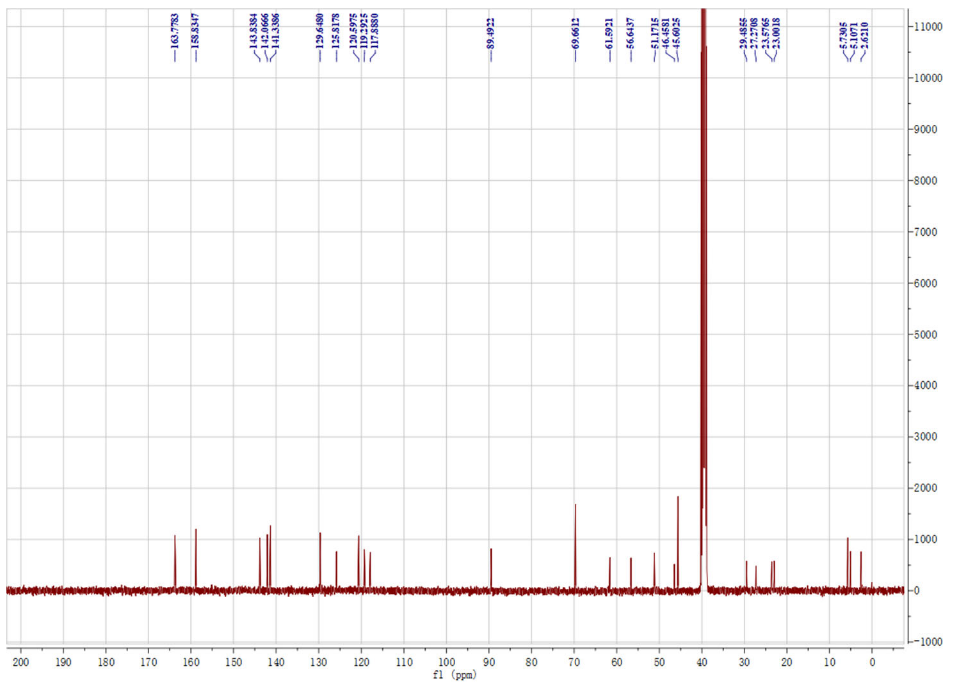
163.7783
158.8347
143.8384
142.0666
141.3386
129.6480
125.8178
120.5975
119.2925
117.8880
89.4922
69.6612
61.5921
56.6437
51.1715
46.4581
45.6025
29.4855
27.2708
23.5765
23.0018
5.7305
5.1071
2.6510
f1 (ppm)

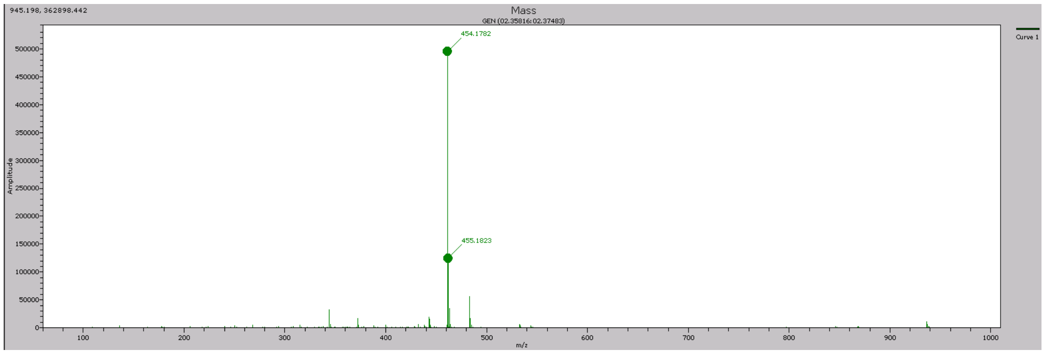
Mass
454.1782
455.1823
Amplitude
m/z
Curve 1

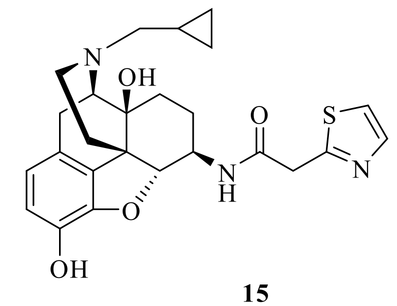

15

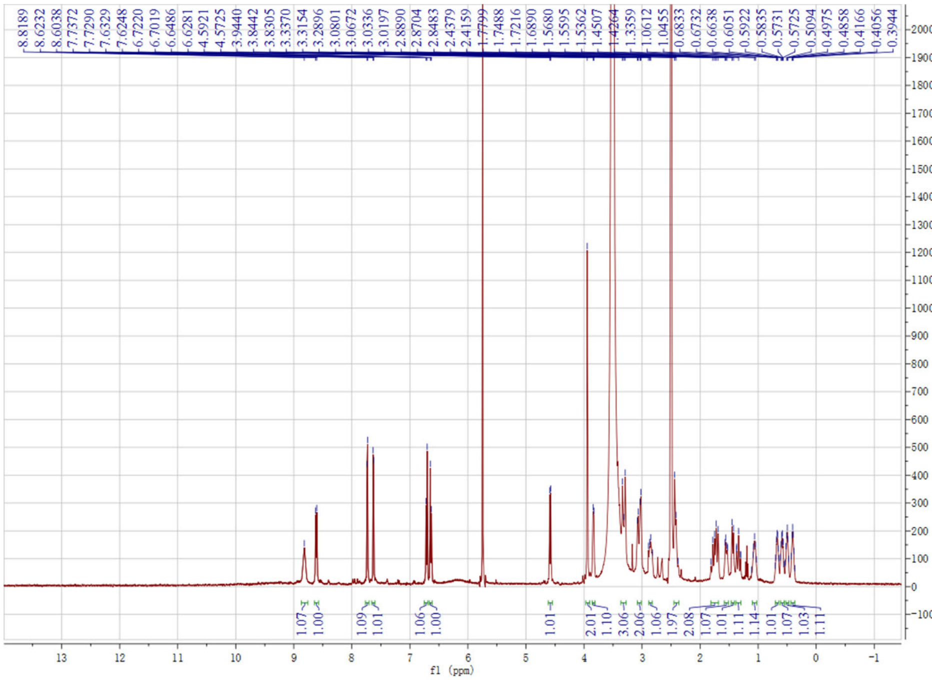
8.8189
8.6232
8.6038
7.7372
7.7290
7.6329
7.6248
6.7220
6.7019
6.6486
6.6281
4.5921
4.5725
3.9440
3.8442
3.8305
3.3370
3.3154
3.2896
3.0801
3.0672
3.0336
3.0197
2.8890
2.8704
2.8483
2.4379
2.4159
1.7799
1.7488
1.7216
1.6890
1.5680
1.5595
1.5362
1.4507
1.4264
1.3359
1.0612
1.0455
0.6833
0.6732
0.6638
0.6051
0.5922
0.5835
0.5731
0.5725
0.5094
0.4975
0.4858
0.4166
0.4056
0.3944
1.07
1.00
1.09
1.01
1.06
1.00
1.01
2.01
1.10
3.06
2.06
1.06
1.97
2.08
1.07
1.01
1.11
1.14
1.01
1.07
1.03
1.11
f1 (ppm)

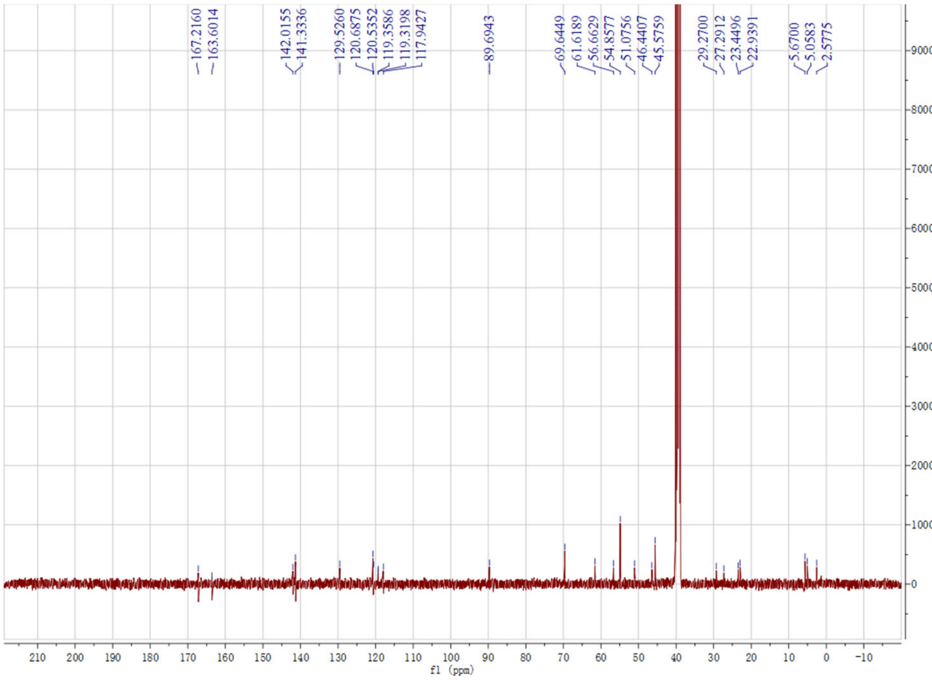
167.2160
163.6014
142.0155
141.3336
129.5260
120.6875
120.5352
119.3586
119.3198
117.9427
89.6943
69.6449
61.6189
56.6629
54.8577
51.0756
46.4407
45.5759
29.2700
27.2912
23.4496
22.9391
5.6700
5.0583
2.5775
f1 (ppm)

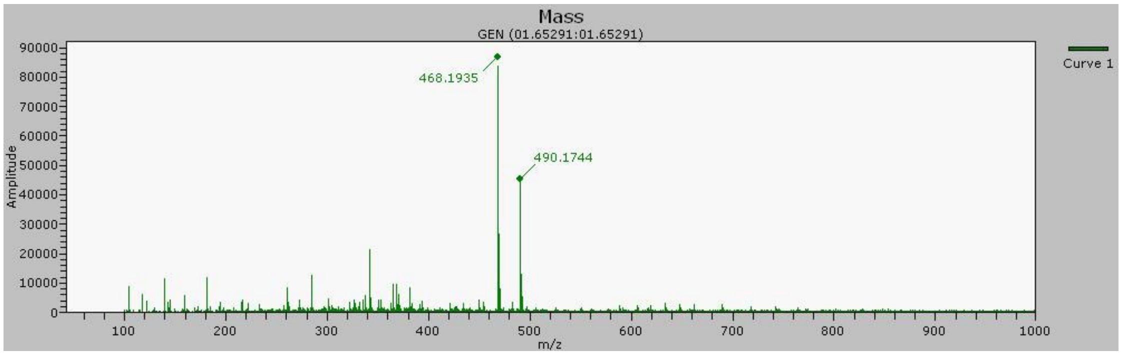
Mass
GEN (01.65291:01.65291)
468.1935
490.1744
Curve 1
Amplitude
m/z

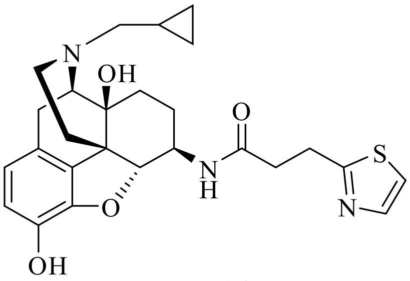
N
OH
O
N
H
S
N
O
OH

16

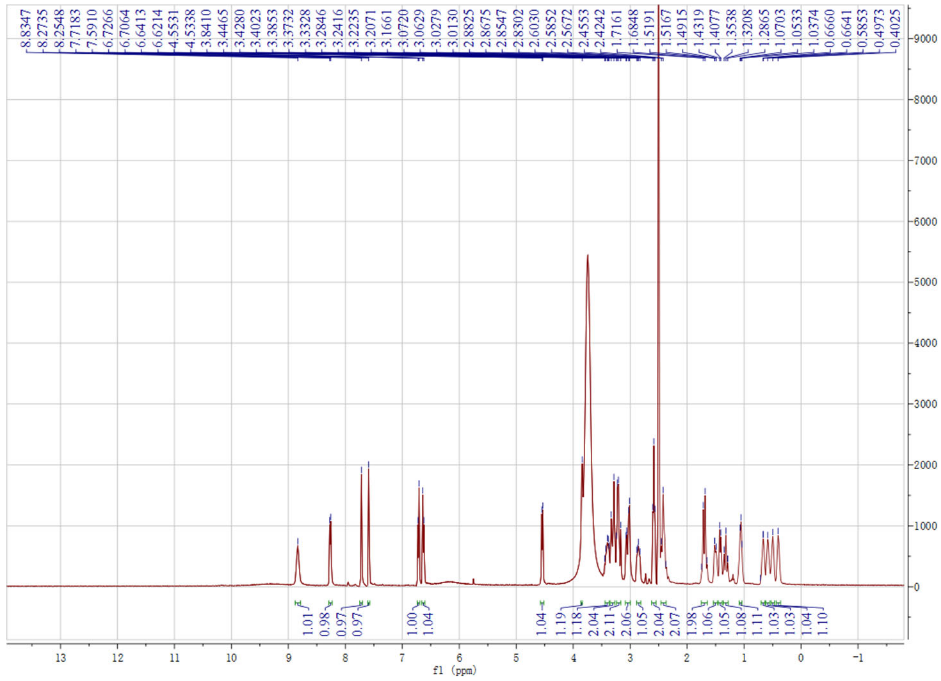
8.8347
8.2735
8.2548
7.7183
7.5910
6.7266
6.7064
6.6413
6.6214
4.5531
4.5338
3.8410
3.4465
3.4280
3.4023
3.3853
3.3732
3.3328
3.2846
3.2416
3.2235
3.2071
3.1661
3.0720
3.0629
3.0279
3.0130
2.8825
2.8675
2.8547
2.8302
2.6030
2.5852
2.5672
2.4553
2.4242
1.7161
1.6848
1.5191
1.5167
1.4915
1.4319
1.4077
1.3538
1.3208
1.2865
1.0703
1.0533
1.0374
0.6660
0.6641
0.5853
0.4973
0.4025
1.01
0.98
0.97
0.97
1.00
1.04
1.04
1.19
1.18
2.04
2.11
2.06
1.05
2.04
2.07
1.98
1.06
1.05
1.08
1.11
1.03
1.03
1.04
1.10
f1 (ppm)

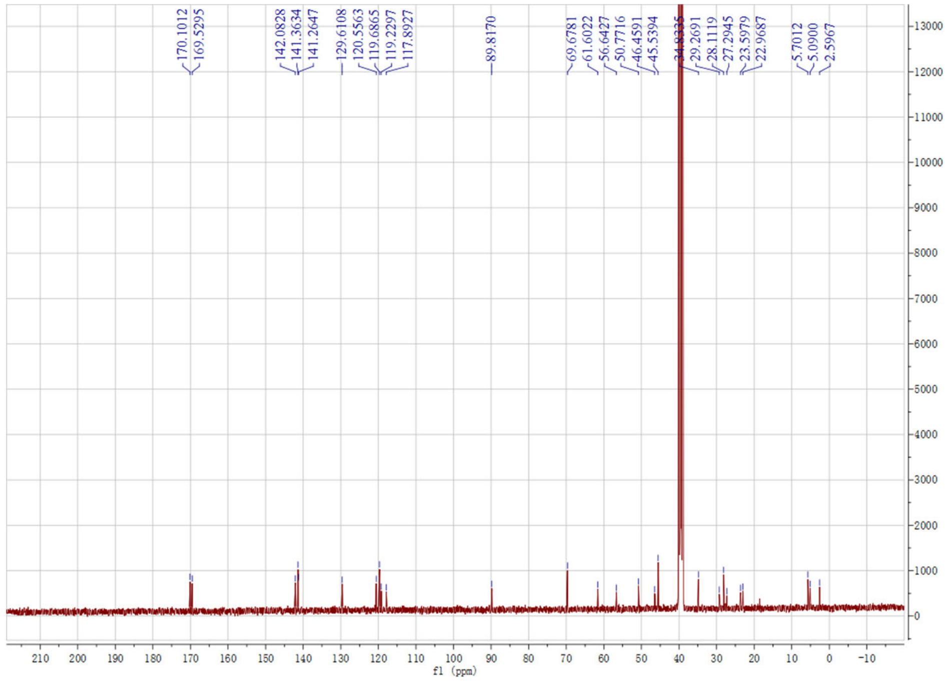
170.1012
169.5295
142.0828
141.3634
141.2647
129.6108
120.5563
119.6865
119.2297
117.8927
89.8170
69.6781
61.6022
56.6427
50.7716
46.4591
45.5394
29.2691
28.1119
27.2945
23.5979
22.9687
5.7012
5.0900
2.5967
f1 (ppm)

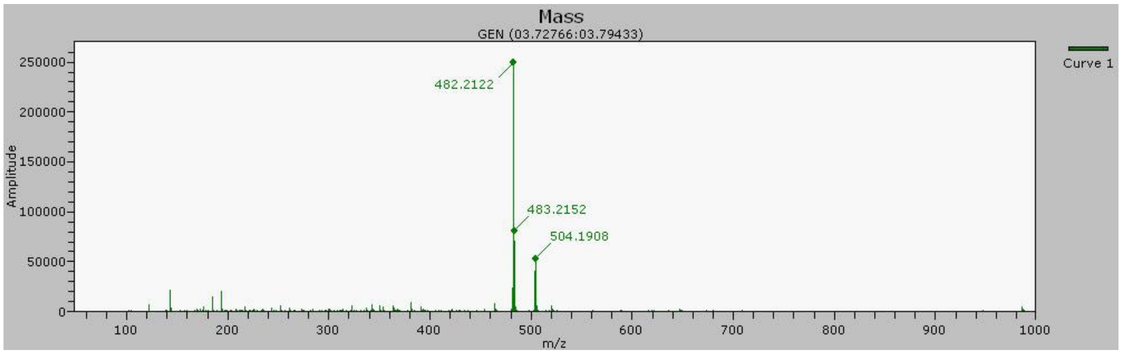
Mass
GEN (03.72766:03.79433)
Curve 1
Amplitude
482.2122
483.2152
504.1908
m/z

2. HPLC chromatograms of target compounds

Blank

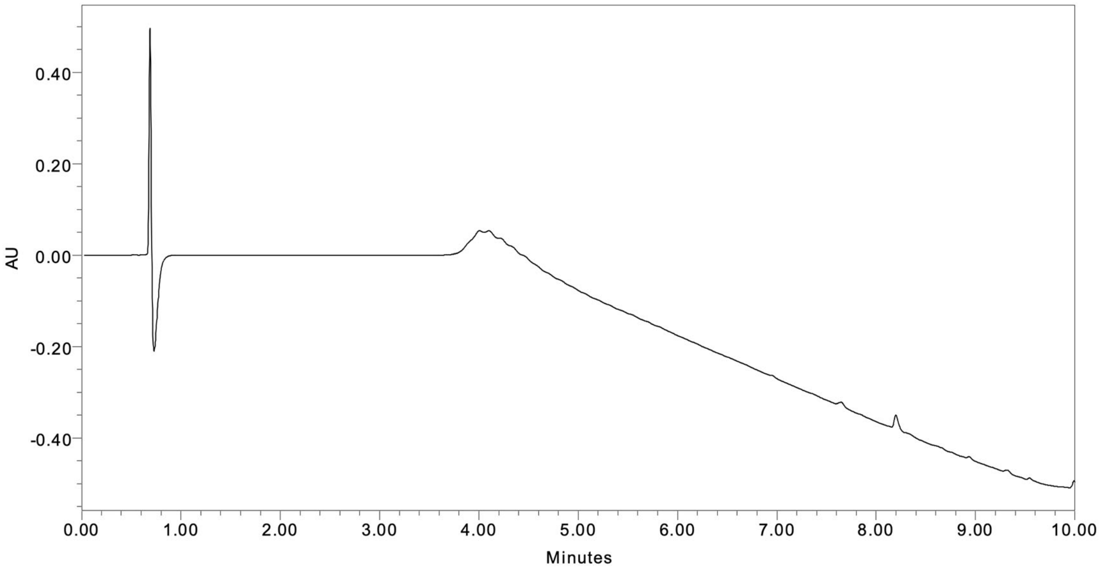

1

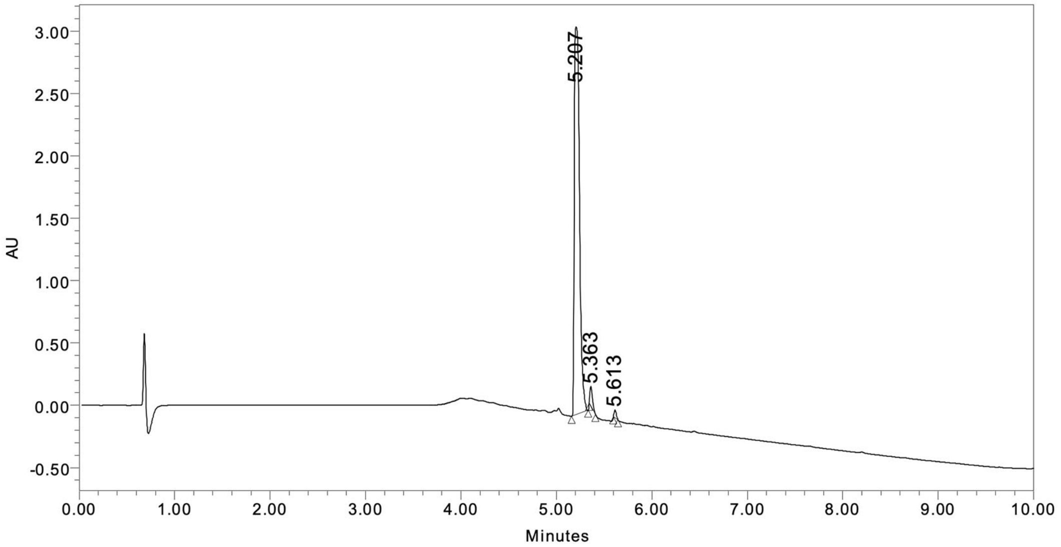

	RT	Area	% Area	Height
1	5.207	11842229	96.85	3108975
2	5.363	289935	2.37	158643
3	5.613	95781	0.78	62398

2

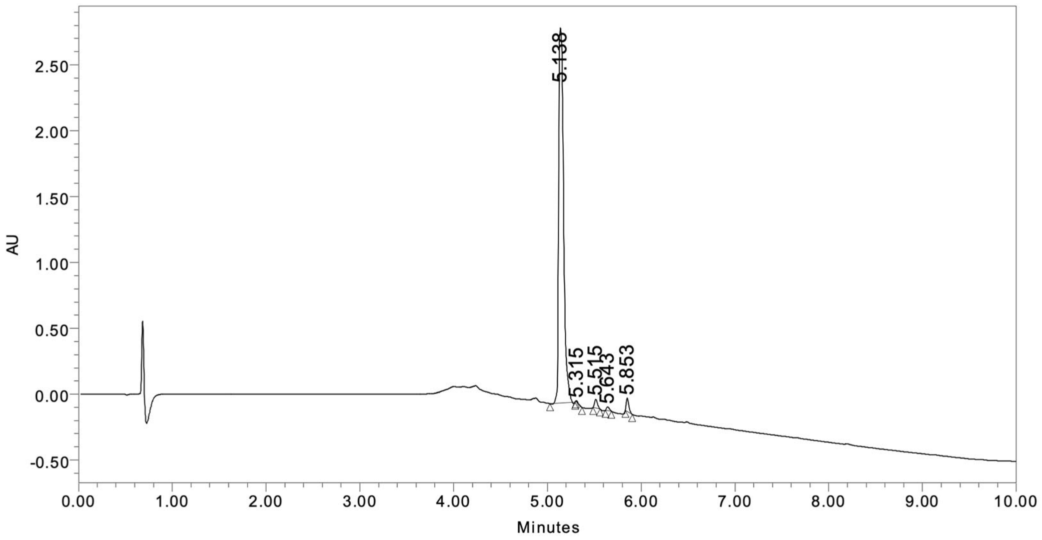

	RT	Area	% Area	Height
1	5.138	10405166	96.29	2846834
2	5.315	13876	0.13	7408
3	5.515	142681	1.32	67582
4	5.643	55823	0.52	27101
5	5.853	187992	1.74	101217

3

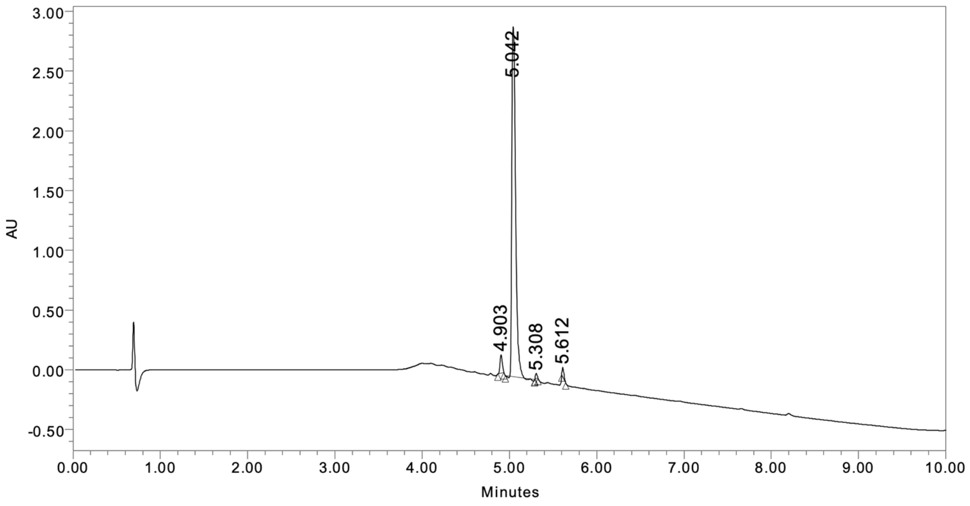

	RT	Area	% Area	Height
1	4.903	275985	2.98	154768
2	5.042	8814903	95.24	2930460
3	5.308	55943	0.60	43026
4	5.612	108562	1.17	79718

4

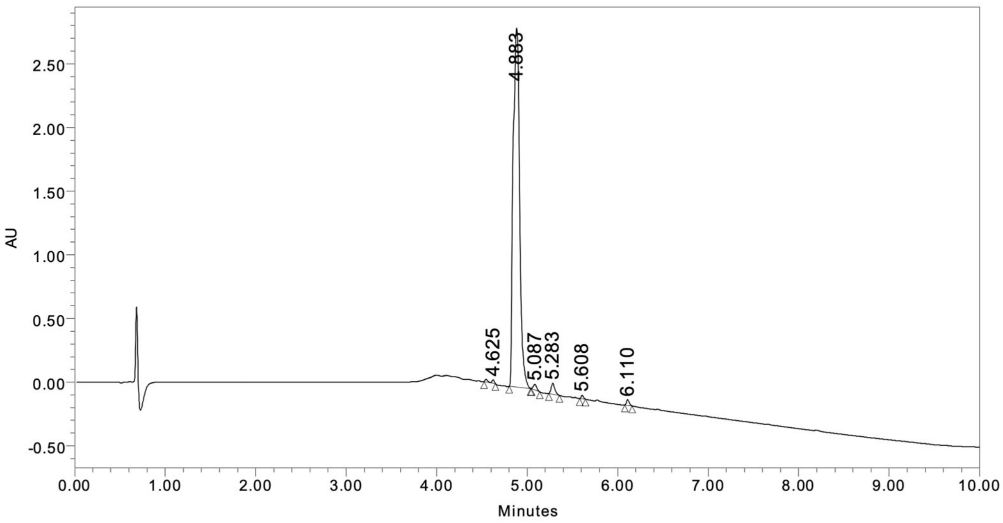

	RT	Area	% Area	Height
1	4.625	77538	0.54	25661
2	4.883	13861221	96.12	2817595
3	5.087	102987	0.71	43204
4	5.283	236234	1.64	85196
5	5.608	48964	0.34	27076
6	6.110	93978	0.65	45567

5

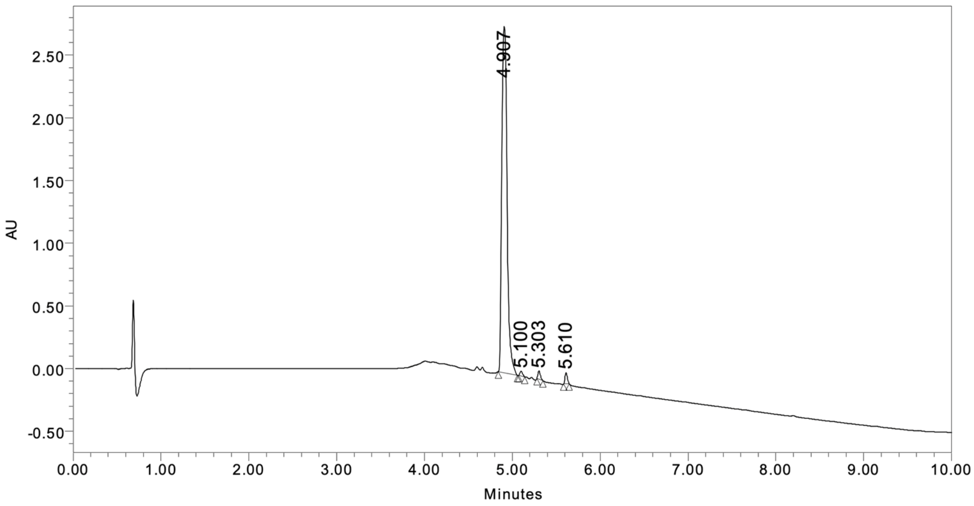

	RT	Area	% Area	Height
1	4.907	11511020	96.92	2761796
2	5.100	75929	0.64	37028
3	5.303	126732	1.07	65260
4	5.610	163526	1.38	90192

6

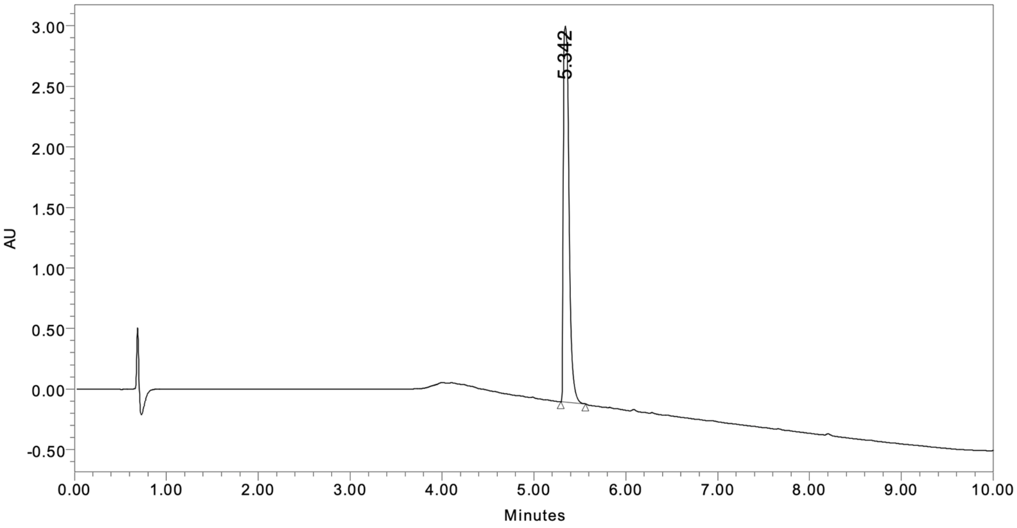

	RT	Area	% Area	Height
1	5.342	13356555	100.00	3104866

7

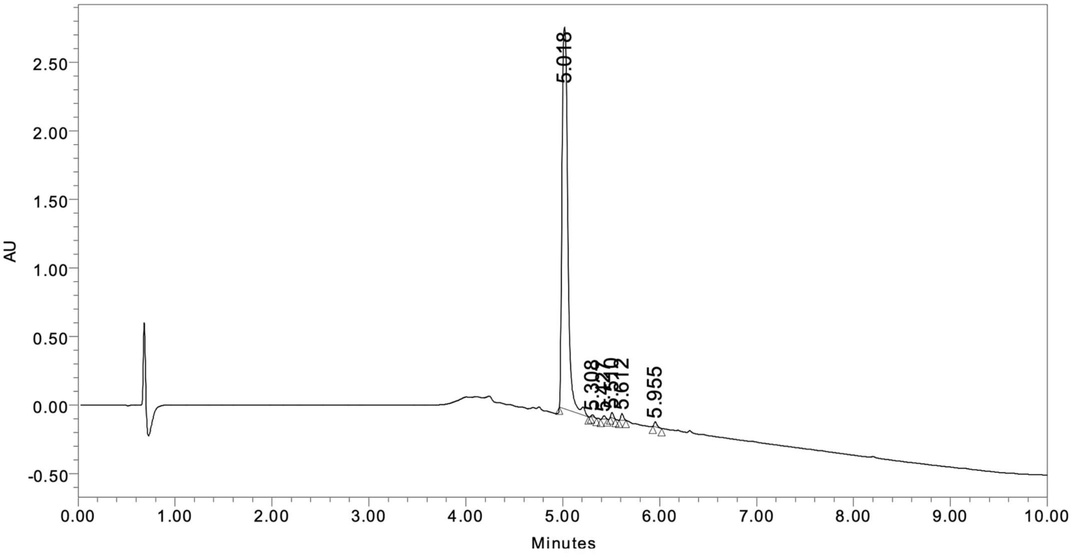

	RT	Area	% Area	Height
1	5.018	11544712	97.46	2784250
2	5.308	23221	0.20	14606
3	5.427	47675	0.40	24664
4	5.510	62056	0.52	36419
5	5.612	94457	0.80	49544
6	5.955	73847	0.62	36767

8

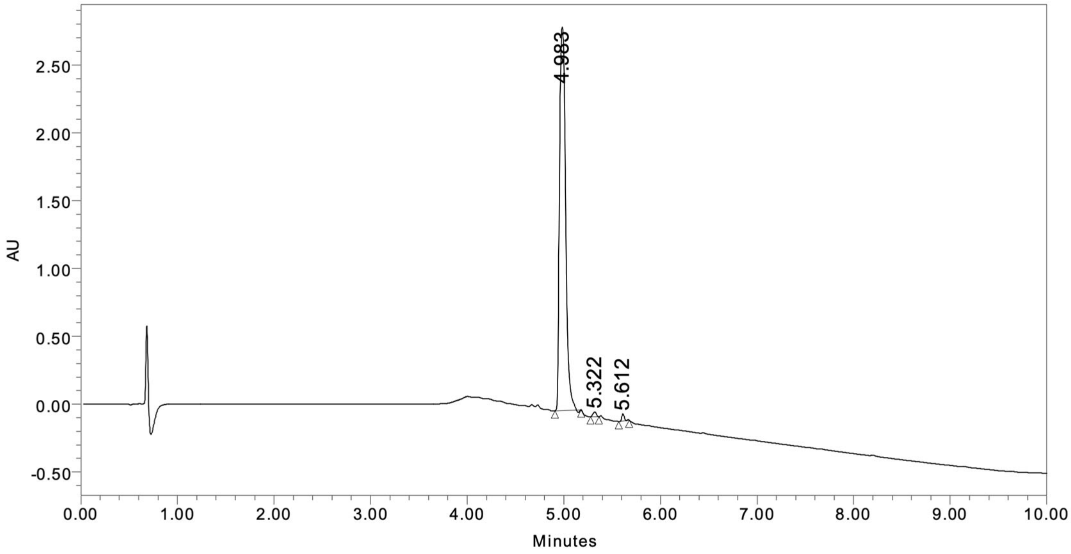

	RT	Area	% Area	Height
1	4.983	12432259	98.51	2826570
2	5.322	89345	0.71	35777
3	5.612	98417	0.78	53066

9

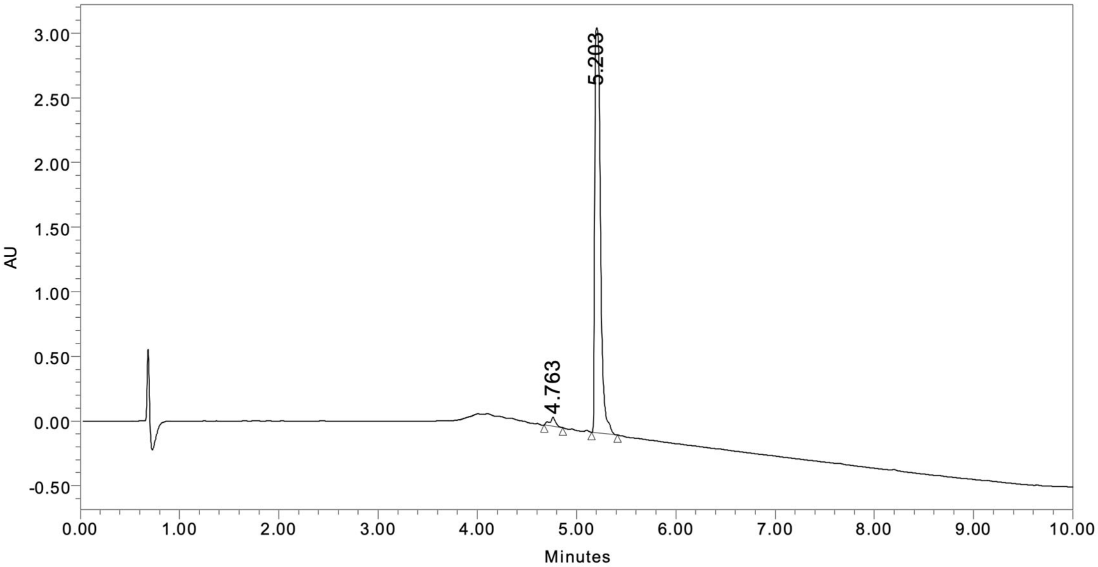

	RT	Area	% Area	Height
1	4.763	279773	2.16	68941
2	5.203	12650437	97.84	3133871

10

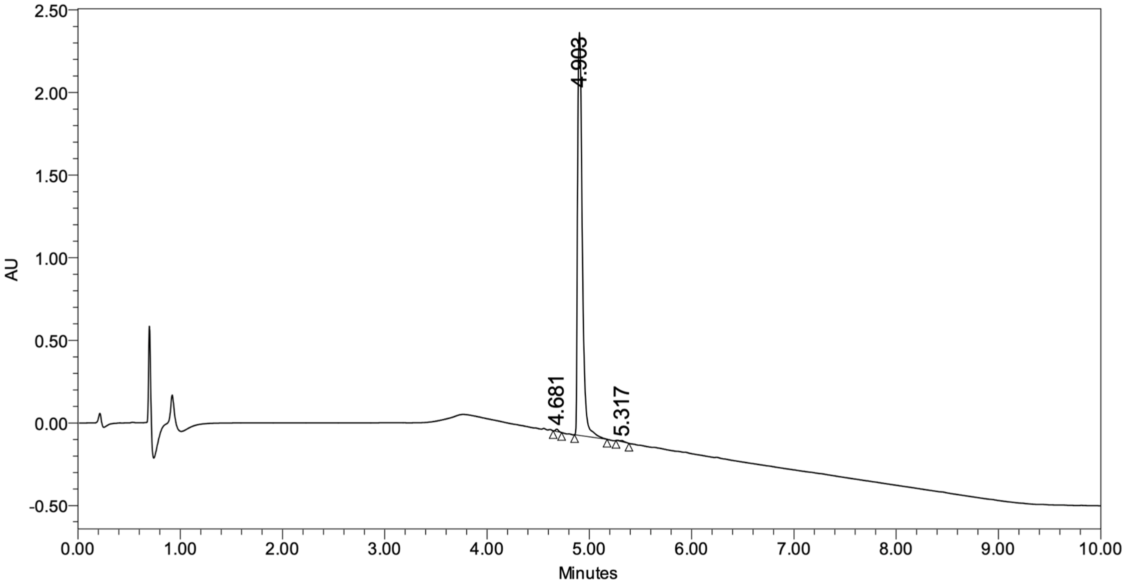

	RT	Area	% Area	Height
1	4.681	28514	0.36	14575
2	4.903	7832635	99.29	2438216
3	5.317	27432	0.35	7749

11

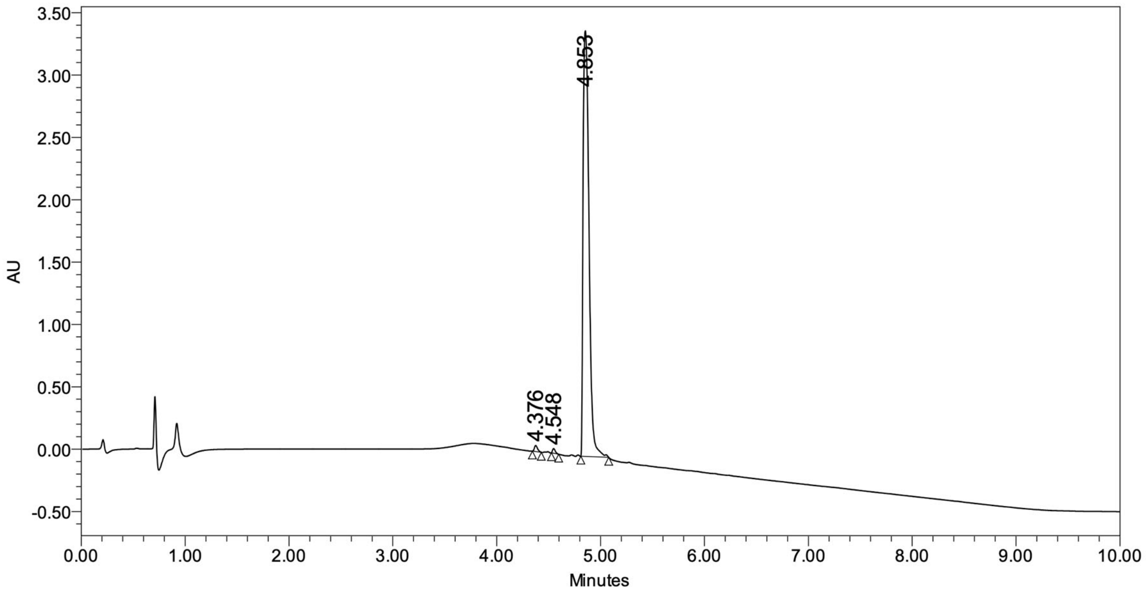

	RT	Area	% Area	Height
1	4.376	102181	0.75	48776
2	4.548	69234	0.51	37686
3	4.853	13431794	98.74	3413190

12

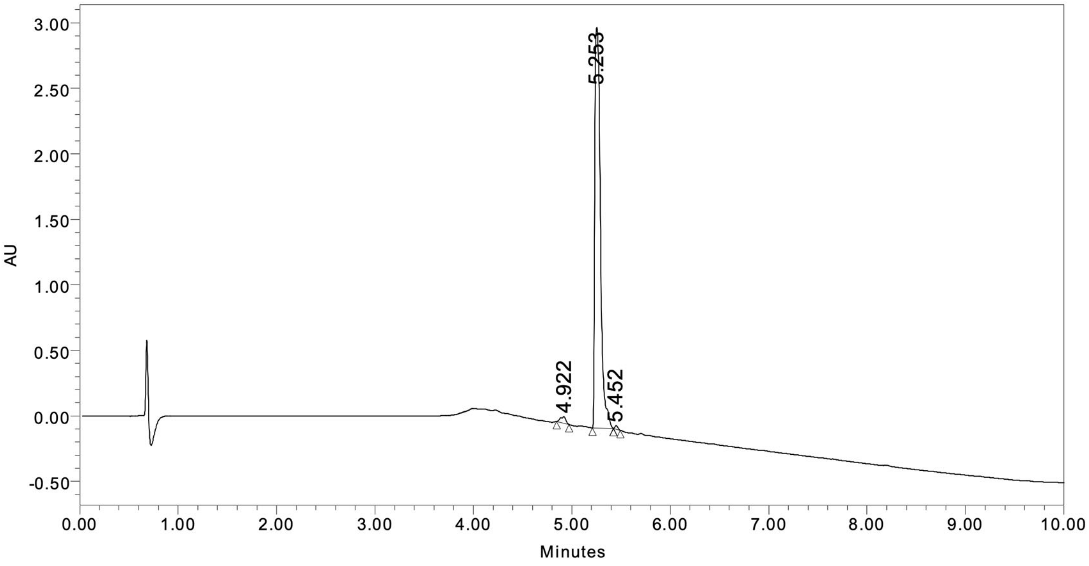

	RT	Area	% Area	Height
1	4.922	180146	1.44	49123
2	5.253	12236409	98.12	3057403
3	5.452	54881	0.44	27820

13

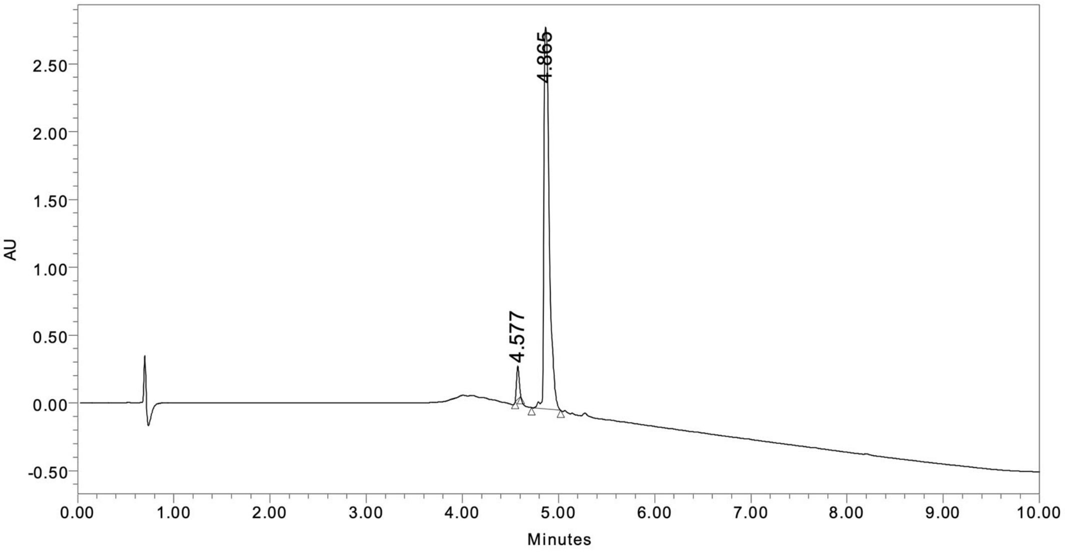

	RT	Area	% Area	Height
1	4.577	449308	3.98	244484
2	4.865	10851999	96.02	2815221

14

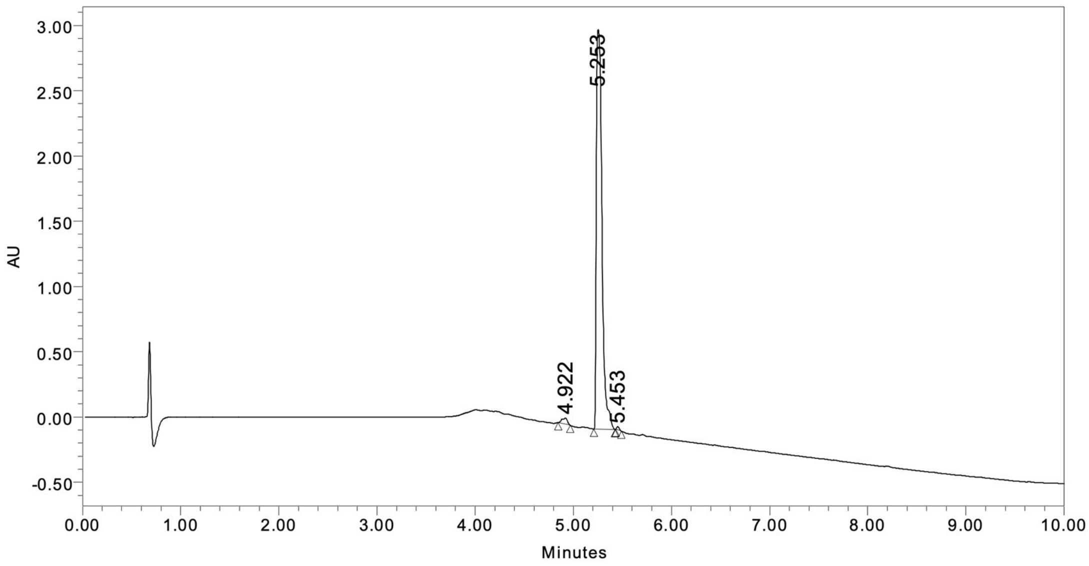

	RT	Area	% Area	Height
1	4.922	175532	1.41	48462
2	5.253	12232929	98.25	3061425
3	5.453	42377	0.34	23723

15

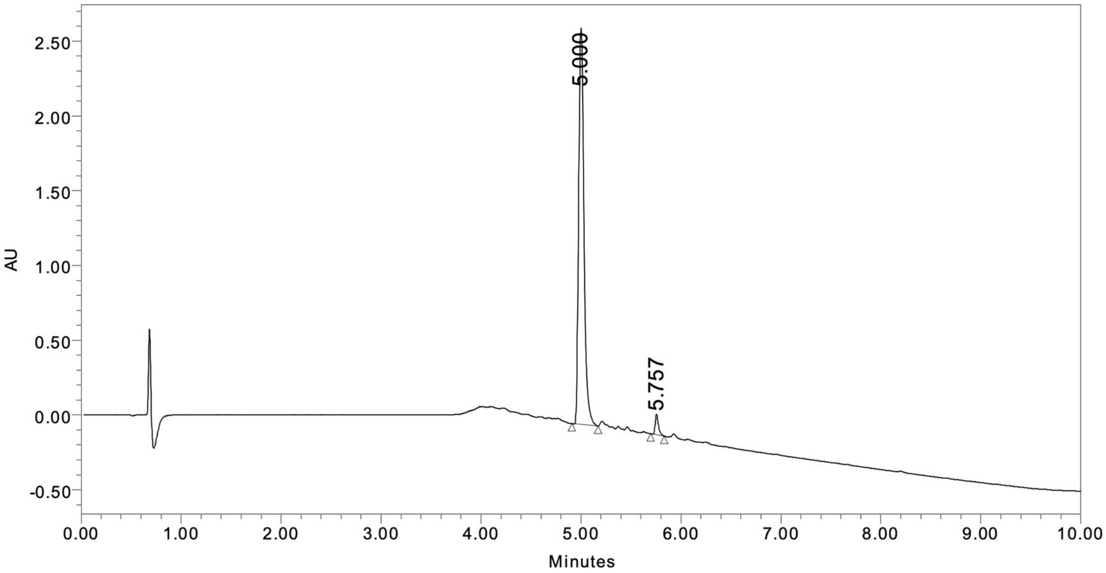

	RT	Area	% Area	Height
1	5.000	9776952	97.01	2650563
2	5.757	301123	2.99	135945

16

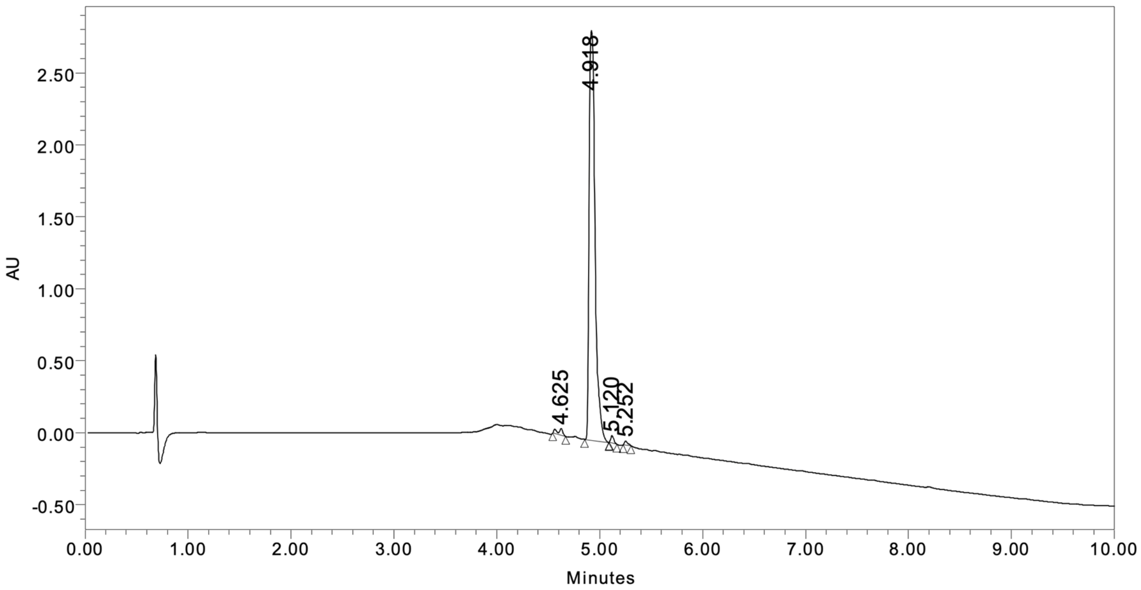

	RT	Area	% Area	Height
1	4.625	159758	1.36	47457
2	4.918	11423482	97.25	2846449
3	5.120	99226	0.84	50044
4	5.252	64609	0.55	29333

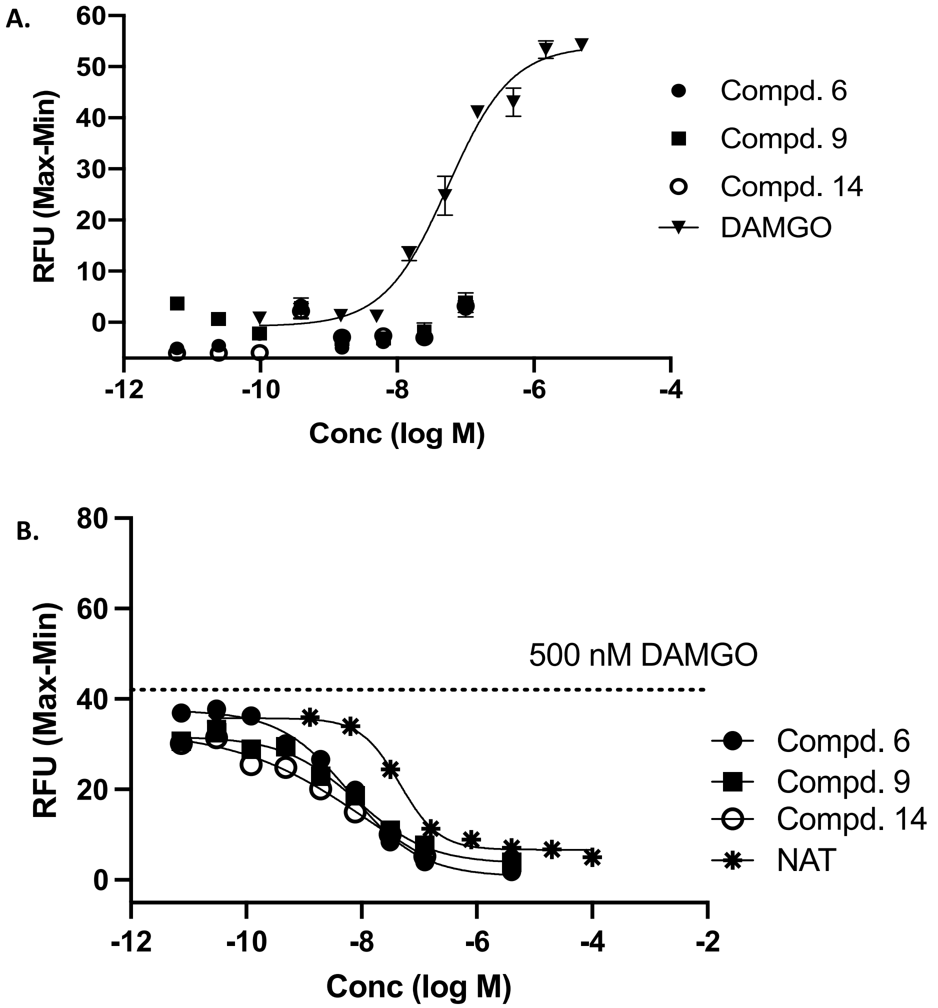

Figure S1. Calcium flux assay of compounds **6**, **9** and **14** in $G\alpha_{qi5}$-transfected mMOR-CHO cells. (A) Compound **6, 9** and **14** exhibited no apparent agonism to increase the intracellular calcium level. DAMGO was used as a control. The EC_{50} value of DAMGO was 54.09 ± 7.78 nM. (B) Compounds **6, 9** and **14** significantly antagonized DAMGO-induced intracellular calcium increase. 500 nM DAMGO in assay buffer was used in the antagonism studies and the data was compared to NAT. The IC_{50} values of NAT and compounds **6**, **9** and **14** were 42.11 ± 4.51 and 5.64 ± 0.24, 6.53 ± 2.28, and 12.37 ± 1.18 nM, respectively. All assays were repeated at least three times, and the data is shown as mean ± SEM.

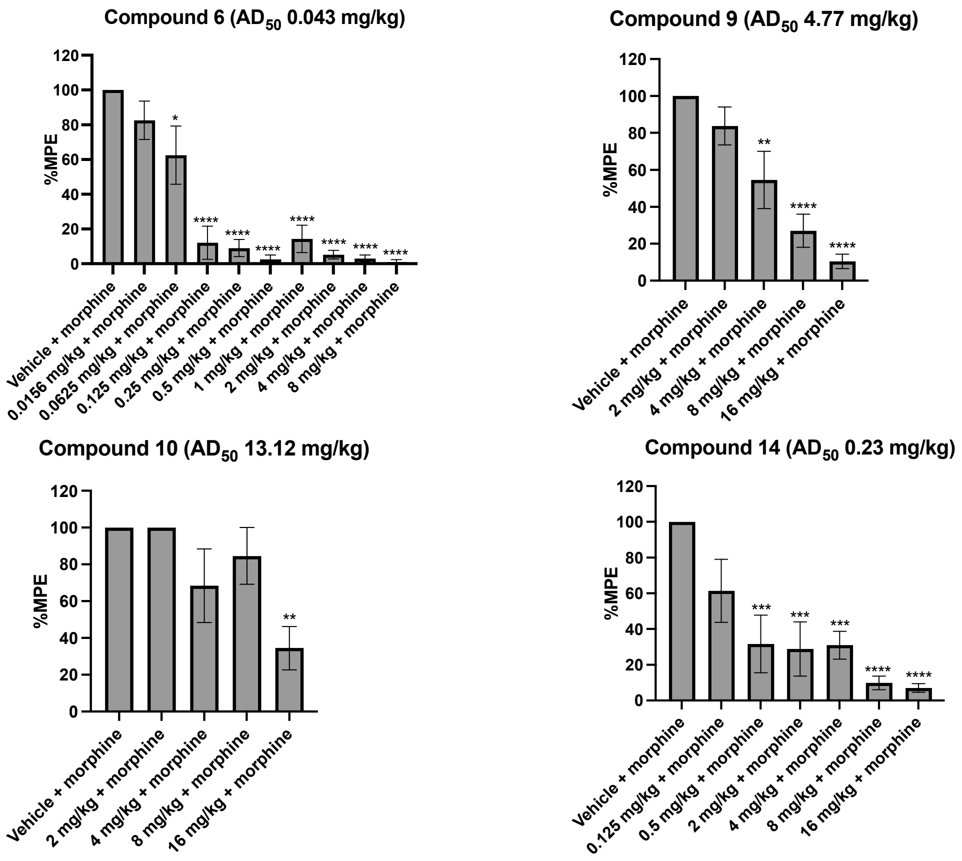

Figure S2. Dose-response study of compounds **6**, **9**, **10**, and **14** to antagonize morphine mediated antinociception.

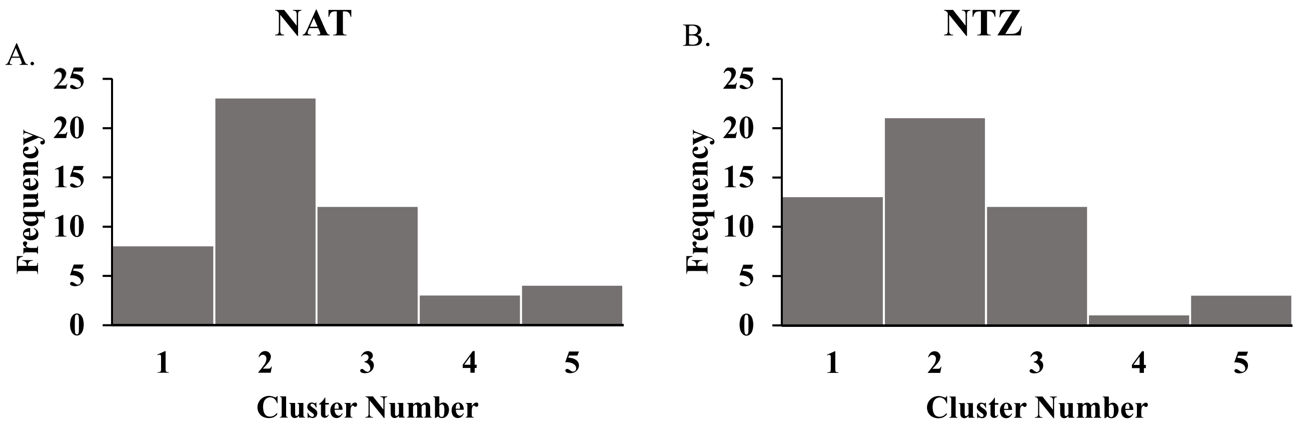

Figure S3. Clustering of the 50 docking solutions of NAT **(A)** and NTZ **(B)** in the inactive conformation of MOR

Table S1. In vitro metabolic stability study result of compound **6** (NTZ) in human liver microsomes at 1.0E-07 M concentration

Incubation Time (min)	% Compound Remaining			Half-life (min)			Clint (μL/min/mg)
	1st	2nd	Mean	1st	2nd	Mean	
0	100	100	100	>60	1120	>60	<38.5
15	97.1	104.5	101				
30	105.3	97.1	101				
45	104.6	101.0	103				
60	96.3	97.1	97				

Table S2. In vitro metabolic stability study result of compound **6** (NTZ) in Sprague-Dawley rat liver microsomes at 1.0E-07 M concentration

Incubation Time (min)	% Compound Remaining			Half-life (min)			Clint (μL/min/mg)
	1st	2nd	Mean	1st	2nd	Mean	
0	100	100	100	>60	>60	>60	<38.5
15	119.6	92.7	106				
30	117.8	111.5	115				
45	99.4	107.4	103				
60	109.1	100.3	105				

Table S3. Cytochrome P450 inhibition study results of compound **6** (NTZ)

CYP isozymes	Test concentrations	% Inhibition		
		1st	2nd	Mean
CYP1A2	1.0E-07 M	14.4	8.4	11.4
	1.0E-05 M	13.1	1.7	7.4
CYP2A6	1.0E-07 M	8.3	4.3	6.3
	1.0E-05 M	10.3	5.3	7.8
CYP2C9	1.0E-07 M	0.9	0.2	0.6
	1.0E-05 M	16.2	16.3	16.3
CYP2C19	1.0E-07 M	17.8	8.5	13.2
	1.0E-05 M	80.7	76.2	78.4
CYP3A4	1.0E-07 M	0.8	19.5	10.1
	1.0E-05 M	14.2	32.8	23.5

Table S4. hERG toxicity study result of compound **6** (NTZ)

Test concentrations	% Inhibition of Tail Current		
	1st	2nd	Mean
1.0E-08 M	2.6	2.6	2.6
1.0E-07 M	7.1	5.0	6.0
1.0E-06 M	18.2	18.8	18.5
1.0E-05 M	56.3	54.7	55.5
1.0E-04 M	88.9	84.7	86.8

Table S5. In-silico physicochemical properties prediction of target compounds and NAT and calculation of CNS MPO scores.[a]

Property / Cpds	ClogP/T0	ClogD (pH 7.4)/T0	TPSA/T0	MW/T0	HBD/T0	pKa (Base)/T0	CNS MPO
**NAT**	2.77/1	2.11/0.95	110.27/0.32	452.57/0.34	3/0.17	7.34/1	3.8
**1**	1.56/1	1.19/1	123.16/0	453.56/0.33	3/0.17	7.23/1	3.5
**2**	1.5/1	1.06/1	123.16/0	467.59/0.23	3/0.17	7.34/1	3.4
**3**	1.26/1	0.89/1	123.16/0	453.56/0.33	3/0.17	7.23/1	3.5
**4**	1.23/1	0.79/1	123.16/0	467.59/0.23	3/0.17	7.34/1	3.4
**5**	1.73/1	1.28/1	123.16/0	481.62/0.13	3/0.17	7.36/1	3.3
**6**	1.83/1	1.49/1	123.16/0	453.56/0.33	3/0.17	7.17/1	3.5
**7**	1.84/1	1.37/1	123.16/0	467.59/0.23	3/0.17	7.32/1	3.4
**8**	2.12/1	1.67/1	123.16/0	481.62/0.13	3/0.17	7.36/1	3.3
**9**	1.56/1	1.19/1	123.16/0	453.56/0.33	3/0.17	7.23/1	3.5
**10**	1.5/1	1.06/1	123.16/0	467.59/0.23	3/0.17	7.34/1	3.4
**11**	1.26/1	0.89/1	123.16/0	453.56/0.33	3/0.17	7.23/1	3.5
**12**	1.23/1	0.79/1	123.16/0	467.59/0.23	3/0.17	7.34/1	3.4
**13**	1.73/1	1.28/1	123.16/0	481.62/0.13	3/0.17	7.36/1	3.3
**14**	1.83/1	1.49/1	123.16/0	453.56/0.33	3/0.17	7.17/1	3.5
**15**	1.84/1	1.37/1	123.16/0	467.59/0.23	3/0.17	7.32/1	3.4
**16**	2.12/1	1.67/1	123.16/0	481.62/0.13	3/0.17	7.36/1	3.3

[a] Physicochemical properties were predicted by ACD/Percepta (v2020.2.0).
